# High Stromal SFRP2 Expression in Urothelial Carcinoma Confers an Unfavorable Prognosis

**DOI:** 10.3389/fonc.2022.834249

**Published:** 2022-03-16

**Authors:** Hong-Yue Lai, Chia-Chun Chiu, Yu-Hsuan Kuo, Hsin-Hwa Tsai, Li-Ching Wu, Wen-Hsin Tseng, Chien-Liang Liu, Chung-Hsi Hsing, Steven K. Huang, Chien-Feng Li

**Affiliations:** ^1^ Center for Precision Medicine, Chi Mei Medical Center, Tainan, Taiwan; ^2^ Department of Medical Research, Chi Mei Medical Center, Tainan, Taiwan; ^3^ Genetics Generation Advancement Corp., Taipei, Taiwan; ^4^ Division of Hematology and Oncology, Department of Internal Medicine, Chi Mei Medical Center, Tainan, Taiwan; ^5^ Division of Urology, Department of Surgery, Chi Mei Medical Center, Tainan, Taiwan; ^6^ Division of Uro-Oncology, Department of Surgery, Chi Mei Medical Center, Tainan, Taiwan; ^7^ Department of Anesthesiology, Chi Mei Medical Center, Tainan, Taiwan; ^8^ Department of Anesthesiology, College of Medicine, Taipei Medical University, Taipei, Taiwan; ^9^ Department of Medical Science Industries, College of Health Sciences, Chang Jung Christian University, Tainan, Taiwan; ^10^ Department of Clinical Pathology, Chi Mei Medical Center, Tainan, Taiwan; ^11^ National Institute of Cancer Research, National Health Research Institutes, Tainan, Taiwan; ^12^ Institute of Precision Medicine, National Sun Yat-Sen University, Kaohsiung, Taiwan; ^13^ Department of Pathology, School of Medicine, College of Medicine, Kaohsiung Medical University, Kaohsiung, Taiwan

**Keywords:** urothelial carcinoma, bladder cancer, upper urinary tract cancer, *SFRP2*, collagen, stroma

## Abstract

**Background:**

Urothelial carcinoma (UC) patients often bear clinical and genetic heterogeneity, which may differ in management and prognosis. Especially, patients with advanced/metastatic UC generally have a poor prognosis and survive for only few months. The Wnt/β-catenin signaling is found to be highly activated in several cancers, including UC. However, accumulated evidence has shown discordance between the Wnt/β-catenin signaling and UC carcinogenesis. Accordingly, we aim to get a better understanding of the molecular characterization of UC, focusing on the Wnt signaling, which may add value to guiding management more precisely.

**Patients and Methods:**

Clinical data and pathological features were retrospectively surveyed. The correlations of secreted Frizzled-related protein 2 (SFRP2) immunoexpression with clinicopathological features were analyzed by Pearson’s chi-square test. The Kaplan–Meier method with a log-rank test was employed to plot survival curves. All significant features from the univariate analysis were incorporated into the Cox regression model for multivariate analysis.

**Results:**

Following data mining on a transcriptome dataset (GSE31684), we identified that 8 transcripts in relation to the Wnt signaling pathway (GO: 0016055) were significantly upregulated in advanced/metastatic bladder tumors. Among these transcripts, the *SFRP2* level showed the most significant upregulation. Additionally, as *SFRP2* is a putative Wnt inhibitor and may be expressed by stroma, we were interested in examining the immunoexpression and clinical relevance of stromal and tumoral SFRP2 in our urothelial carcinoma cohorts containing 295 urinary bladder UC (UBUC) and 340 upper urinary tract UC (UTUC) patients. We observed that high SFRP2 expression in stroma but not in tumors is significantly linked to aggressive UC features, including high tumor stage and histological grade, positive nodal metastasis, the presence of vascular and perineural invasion, and high mitotic activity in UBUC and UTUC. Moreover, high stromal SFRP2 expression significantly and independently predicted worse clinical outcomes in UBUC and UTUC. Utilizing bioinformatic analysis, we further noticed that stromal SFRP2 may link epithelial–mesenchymal transition (EMT) to UC progression.

**Conclusion:**

Collectively, these results imply that stromal SFRP2 may exert oncogenic function beyond its Wnt antagonistic ability, and stromal SFRP2 expression can provide prognostic and therapeutic implications for UC patients.

## Introduction

Urothelial carcinoma (UC) is a malignancy derived from the transitional epithelium of the urinary tract, which is also known as transitional cell carcinoma. Although UC is mainly found in the urinary bladder (UBUC), the incidence of upper urinary tract UC (UTUC), including the ureter and renal pelvis, is increasing in Taiwan (1). UC patients often bear clinical and genetic heterogeneity, which may differ in management and prognosis. For UBUC, 70–80% of patients are non-muscle-invasive bladder cancer (NMIBC) at initial diagnosis, and the standard treatment is transurethral resection of bladder tumor (TURBT) with subsequent intravesical instillation (2). However, 50–70% of patients experience local recurrence, and 10–15% of patients progress to muscle-invasive or metastatic disease (3). In contrast, 20–30% of UBUC patients are initially diagnosed with muscle-invasive bladder cancer (MIBC) or metastatic disease, and radical cystectomy is indicated as the standard therapy (4). Nevertheless, recurrence and metastasis still occur in 15–50% of patients following radical surgery (4). For UTUC, curative nephroureterectomy with bladder cuff excision is recommended for most patients (5). In addition, cisplatin-based postoperative adjuvant chemotherapy is given for advanced/metastatic UBUC or UTUC patients, but the clinical outcomes remain disappointing due to chemoresistance (6). Since UBUC and UTUC are featured by clinical heterogeneity, the addition of genetic information may improve management and prognosis.

The Wingless-related integration site (Wnt)/β-catenin signaling pathway is involved in diverse physiological processes, including embryonic development, cell proliferation and differentiation, and tissue homeostasis and regeneration (7). The canonical Wnt/β-catenin signaling is triggered by the binding of Wnt ligands to the low density lipoprotein receptor-related protein 5/6 (LRP5/6) receptors and Frizzled (FZD) receptors on the cell surface. Subsequently, β-catenin is undegraded and increased in the cytosol and translocates to the nucleus and binds to T-cell factor (TCF)/lymphoid enhancer-binding factor (LEF) transcription factors, resulting in the upregulation of Wnt target genes (8). In addition to prominently described in colorectal cancer, the Wnt/β-catenin signaling is found to be highly activated in several cancers, including UC (9). This leads to the development of various Wnt/β-catenin inhibitors such as targeting Wnt ligand/receptor interface for cancer therapies (8). However, accumulated investigations have shown incompatible evidence between the Wnt/β-catenin signaling and UC carcinogenesis (10, 11). Additionally, the aberrant Wnt/β-catenin signaling is not only restricted to cancer cells but also implicated in dynamic interactions with the tumor microenvironment (TME) and immune system in UC (12). These observations further emphasize the molecular heterogeneity of UC, and a deeper understanding of the molecular characterization of UC may come up with more hints for how such pathways be therapeutically targeted.

The secreted Frizzled-related protein 2 (*SFRP2*) gene, which is located on chromosome 4q31.3 in humans, encodes a glycoprotein containing an Frizzled-like cysteine-rich domain. As this N-terminal cysteine-rich domain shares substantial sequence similarity with Wnt binding domain of FZD receptors, SFRP2 was initially considered to antagonize the Wnt signaling by preventing binding of Wnt ligands to FZD receptors, which may inhibit tumor development (13). However, recent study has indicated that SFRP2 can promote tumor angiogenesis *via* the noncanonical Wnt/Ca^2+^ signaling (14). Moreover, upon secreted to the extracellular matrix (ECM), SFRP2 has been linked to the fibronectin-integrin complex, which can promote cell adhesion and block apoptosis in canine mammary gland tumors (15). Also, high level of SFRP2 in serum has been correlated with a poor prognosis in breast cancer patients (16). A similar correlation between high SFRP2 protein expression and poor prognosis was observed in osteosarcoma, which usually develops in the osteoblast cells from bone (17). In addition, SFRP2 is also known as stromal cell-derived factor 5 (SDF5), which was identified by a cDNA screen for secreted proteins in bone marrow stromal cells (18). Impressively, it has been reported that SFRP2 expression in tumors may be conferred by stroma (19). These reflect the complicated regulation of SFRP2 in tumors and their microenvironment, which is composed of stromal cells, immune cells, and the ECM. The TME characteristics have also been comprehensively described in UC (20). Since *SFRP2* is specifically expressed in the urinary bladder and the contribution of stromal cell–tumor cell communication to UC progression remained to be determined, we were interested in exploring the role of SFRP2 in UBUC and UTUC, thereby improving prognosis and therapy.

## Patients and Methods

### Transcriptomic Data Analysis

A transcriptome dataset (GSE31684), incorporating 93 bladder cancer patients who were managed by radical cystectomy, from the GEO database (NCBI) was used for data mining. The raw data of all probe sets were analyzed without preselection or filtering. To quantify the expression levels of all transcripts, we imported the raw CEL files into the statistical software Nexus Expression 3 (BioDiscovery, El Segundo, CA, USA). According to tumor invasion and metastasis determined by clinical assessment, a comparative analysis (invasive vs. noninvasive and metastatic vs. nonmetastatic) was conducted under supervision. We underscored differentially expressed genes with special interest to the Wnt signaling pathway (GO: 0016055) and further selected those with a log2-transformed expression fold change > 0.2 and a *p*-value less than 0.01 for further analysis.

### Patient Eligibility and Enrollment

This study was approved by the Institutional Review Board of Chi Mei Medical Center (10501005). We enrolled 340 UTUC and 295 UBUC patients who had curative surgery from 1996 to 2004, and all specimens with the informed consents were procured from our biobank. Clinical data, pathological features, and clinical outcomes were retrospectively obtained from the patients’ medical records. Patients with preoperative neoadjuvant chemotherapy, other malignancies, or missing clinical data were not included. With curative purpose, 10 UTUC patients received ureterectomy and 330 UTUC patients underwent nephroureterectomy with bladder cuff excision. Cisplatin-based postoperative adjuvant chemotherapy was given for 29 out of 106 UTUC patients with pT3 or pT4 status or nodal involvement. On the other hand, for patients with superficial UBUC (pTa or pT1), TURBT with or without intravesical Bacillus Calmette–Guérin (BCG) was performed. For those with tumor recurrence, radical cystectomy was further performed. Patients with muscle-invasive UBUC were subjected to radical cystectomy with bilateral pelvic lymph node dissection. Similarly, cisplatin-based postoperative adjuvant chemotherapy was given for UBUC patients with pT3 or pT4 stage disease or nodal metastasis. The median/mean follow-up duration was 38.9/44.7 and 23.1/30.8 months for UTUC and UBUC, respectively.

### Histopathological and Immunohistochemical Assessments

Tumor stages and histological grades were appraised on hematoxylin and eosin (H&E) staining of all cases according to the 7th edition of the American Joint Committee on Cancer (AJCC) staging system (21) and the World Health Organization (WHO) classification criteria (22), respectively. Tumor location and multifocality were used to evaluate clinical outcomes in UTUC but not in UBUC. Vascular invasion and perineural invasion were determined by the presence of tumor emboli in the vascular channels and tumor nests surrounding the nerve bundles, respectively. The mitotic rate was determined by calculating mitotic figures per 10 high-power fields (HPFs; 400x light microscopic magnification). We defined the mitotic rate less than 10/10 HPFs as low mitotic activity and the mitotic rate equal to or beyond 10/10 HPFs as high mitotic activity. Immunohistochemistry was performed based on our previous study (23), and slides were stained with an anti-SFRP2 antibody. Two independent pathologists (Chien-Feng Li and Wan-Shan Li) appraised SFRP2 immunoreactivity by integrating the percentage of stained tumor cells and intensity of staining of UC cells to produce the H-score as previously described (24). The H-score was determined with the following equation: H-score = Σ*Pi* (*i* + 1), where *i* is the intensity of stained tumor cells (0 to 3+) and *Pi* is the percentage of staining for each intensity, ranging from 0% to 100%. According to the H-score, SFRP2 expression levels were divided into low (less than the median) and high (above or equal to the median) groups.

### Functional Annotation of The Cancer Genome Atlas (TCGA) Data

Utilizing the cBioPortal web platform (http://cbioportal.org), the correlations of the mRNA level of *SFRP2* with its coexpressed transcripts in the UTUC and UBUC datasets from the TCGA database were downloaded. To further realize the functional roles of SFRP2 in UC, the top 194 overlapping transcripts co-upregulated with *SFRP2* between UTUC and UBUC were examined utilizing the Gene Ontology (GO) classification system (http://geneontology.org/) according to three functional groups (biological processes, molecular functions, or cellular components) and were ranked by fold enrichment. To plot representative GO terms, an R script with ggplot2 package was used.

### Statistical Analysis

All statistical analyses were executed in SPSS software version 22.0 (IBM Corporation, Armonk, NY, USA), and two-tailed tests with a *p*-value less than 0.05 were considered statistically significant. We analyzed three endpoints: disease-specific survival (DSS), metastasis-free survival (MeFS), and local recurrence-free survival (LRFS). DSS was measured from curative surgery to the time of cancer death, and MeFS and LRFS were measured from curative surgery to the first metastasis and local recurrence, respectively. Pearson’s chi-square test was used to appraise the correlations of clinicopathological variables with SFRP2 expression. The Kaplan–Meier method with a log-rank test was employed to plot survival curves. All significant characteristics from the univariate analysis were entered into the Cox regression model for multivariate analysis to find independent prognostic factors.

## Results

### *SFRP2* Is the Most Significantly Upregulated Gene Related to the Wnt Signaling During UC Invasion and Metastasis

**Figure 1:**
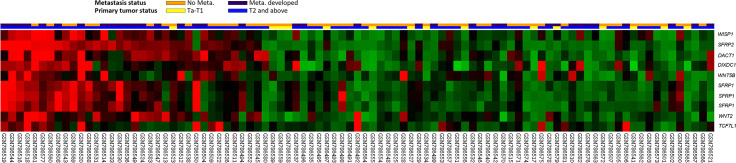
Expression profiles of the top 8 genes correlated with the Wnt signaling pathway in advanced/metastatic bladder tumors. The expression levels of upregulated and downregulated genes are marked in red and green, respectively. A comparative analysis (invasive vs. noninvasive and metastatic vs. nonmetastatic) was conducted under supervision. We identified *SFRP2* as the most considerably upregulated gene related to the Wnt signaling pathway (GO: 0016055) among bladder cancer patients with advanced/metastatic disease.

**Figure 2:**
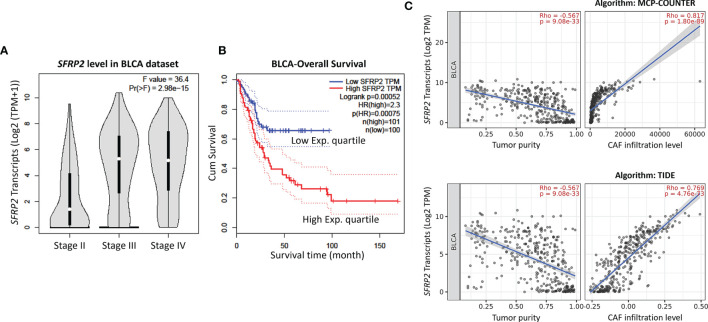
High *SFRP2* mRNA level is correlated with advanced stage disease, inferior overall survival, and CAF infiltration. **(A)** The correlations between the mRNA levels of *SFRP2* and bladder cancer progression. **(B)** The impact of *SFRP2* mRNA levels on overall survival in bladder cancer. These data were acquired from the GEPIA database. **(C)** The correlations among the mRNA levels of *SFRP2*, tumor purity, and CAF infiltration in bladder cancer. These data were estimated using the MCP-COUNTER and TIDE algorithms from the TIMER2.0 database. BLCA, bladder urothelial carcinoma.

To identify promising genes related to UC progression, a public dataset (GSE31684), incorporating 93 bladder cancer patients who were managed by radical cystectomy, was used for data mining. Among these patients, 78 were diagnosed as muscle-invasive disease (pT2–pT4), and 15 were determined as having superficial disease (pTa or pT1). We identified 10 probes covering 8 transcripts zooming in the Wnt signaling pathway (GO: 0016055) (**Table 1** and **Figure 1**). Phylogenetically, SFRP1, SFRP2, and SFRP5 belong to the same subfamily, whose members share homology in their cysteine-rich domain (25). In this study, we found that both the *SFRP1* and *SFRP2* transcripts are significantly increased in tumors with muscle invasiveness and distal metastasis, but the *SFRP2* level showed the most prominent upregulation among all identified transcripts. Nevertheless, using the Gene Expression Profiling Interactive Analysis (GEPIA) database (http://gepia.cancer-pku.cn/detail.php?gene=SFRP1) (http://gepia.cancer-pku.cn/detail.php?gene=SFRP2), which incorporates the TCGA data, we noticed that both the *SFRP1* and *SFRP2* transcripts significantly decrease in bladder urothelial carcinoma (*n* = 404) compared to their paired normal tissue (*n* = 28). Intriguingly, the violin plots showed that both the mRNA levels of *SFRP1* and *SFRP2* significantly increase with the progression of bladder cancer (from stage II to stage IV) (**Supplementary Figure 1A** and **Figure 2A**). However, only bladder tumors with high mRNA level of *SFRP2* (*n* = 101) significantly conferred inferior overall survival compared with those with low *SFRP2* mRNA level (*n* = 100) (*p* = 0.00052) (**Supplementary Figure 1B** and **Figure 2B**).

**Table 1:** Summary of 8 significantly altered genes associated with the Wnt signaling pathway (GO: 0016055) and UC invasion and metastasis (GSE31684).

Probe	MIBC vs. NMIBC		Distal Meta.^&^ vs. No Meta.		Gene Symbol	Gene Title	Biological Process
	Log2 ratio	*p*-value	Log2 ratio	*p*-value			
202035_s_at	0.3856	0.0023	0.398	<0.0001	*SFRP1*	secreted frizzled-related protein 1	Wnt receptor signaling pathway, anatomical structure morphogenesis, anti-apoptosis, cell differentiation, multicellular organismal development, signal transduction
202036_s_at	0.668	0.0019	0.6812	<0.0001	*SFRP1*	secreted frizzled-related protein 1	Wnt receptor signaling pathway, anatomical structure morphogenesis, anti-apoptosis, cell differentiation, multicellular organismal development, signal transduction
202037_s_at	0.7204	0.0005	0.7285	<0.0001	*SFRP1*	secreted frizzled-related protein 1	Wnt receptor signaling pathway, anatomical structure morphogenesis, anti-apoptosis, cell differentiation, multicellular organismal development, signal transduction
205648_at	0.6169	0.0014	0.4056	0.0061	*WNT2*	wingless-type MMTV integration site family member 2	Wnt receptor signaling pathway, Wnt receptor signaling pathway; calcium modulating pathway, multicellular organismal development
206796_at	0.5217	<0.0001	0.2914	0.0025	*WISP1*	WNT1 inducible signaling pathway protein 1	Wnt receptor signaling pathway, cell adhesion, cell-cell signaling, regulation of cell growth, signal transduction
214724_at	0.8864	<0.0001	0.4693	0.0005	*DIXDC1*	DIX domain containing 1	Wnt receptor signaling pathway, multicellular organismal development
219179_at	0.8579	<0.0001	0.3414	0.0061	*DACT1*	dapper; antagonist of beta-catenin; homolog 1 (Xenopus laevis)	Wnt receptor signaling pathway, multicellular organismal development
221016_s_at	0.4905	0.0005	0.4551	<0.0001	*TCF7L1*	transcription factor 7-like 1 (T-cell specific; HMG-box)	Wnt receptor signaling pathway, axial mesoderm morphogenesis, determination of anterior/posterior axis; embryo, establishment and/or maintenance of chromatin architecture, positive regulation of transcription from RNA polymerase II promoter; mitotic, regulation of Wnt receptor signaling pathway, regulation of transcription; DNA-dependent, transcription
221029_s_at	0.3783	0.0034	0.277	0.0056	*WNT5B*	wingless-type MMTV integration site family; member 5B	Wnt receptor signaling pathway, Wnt receptor signaling pathway; calcium modulating pathway, multicellular organismal development
223121_s_at	2.1375	<0.0001	1.0374	0.0019	*SFRP2*	secreted frizzled-related protein 2	Wnt receptor signaling pathway, anterior/posterior pattern formation, cell differentiation, multicellular organismal development, somitogenesis

^&^Development of subsequent metastasis.

In the bladder, stromal cells have been reported to be crucial for urothelial proliferation (26), and some MIBC patients are also featured by stroma-rich (smooth muscle cells and fibroblasts) subtype (27). However, little attention was paid to the crosstalk between stromal cells and tumors in UC. Using the Tumor Immune Estimation Resource (TIMER) database version 2.0 (28), which integrates the TCGA data, we observed that both the *SFRP1* and *SFRP2* transcripts are significantly negatively correlated with tumor purity (the percentage of cancer cells in a sample) and positively correlated with cancer-associated fibroblast (CAF) infiltration in bladder urothelial carcinoma, but the *SFRP2* gene showed a more distinguished correlation (**Supplementary Figure 2** and **Figure 2C**). These observations encouraged us to further survey the immunoexpression and clinical relevance of stromal and tumoral SFRP2 in our UTUC and UBUC cohorts.

### Clinicopathological Characteristics of the UC Patients

We recruited 340 UTUC and 295 UBUC patients who had curative surgery from 1996 to 2004, and all samples were procured from our biobank (**Table 2**). In terms of UTUC, 62 (18.2%) patients showed multifocal tumors, and 49 (14.4%) patients had coexistent ureteral/renal pelvic tumors. There were 28 (8.2%) patients with lymph node metastasis, 159 (46.8%) patients with advanced stage disease, and 284 (83.5%) patients with high histological grade tumors. Perineural invasion was detected in 19 (5.6%) patients, and vascular invasion was found in 106 (31.2%) patients. Additionally, 167 (49.1%) samples were defined as high mitotic activity. In the context of UBUC, there were 29 (9.8%) patients with metastatic lymph nodes, 123 (41.7%) patients with muscle invasiveness, and 239 (81%) patients with high histological grade tumors. Vascular and perineural invasion were detected in 49 (16.6%) and 20 (6.8%) patients, respectively. Furthermore, 156 (52.9%) specimens were determined as having high mitotic activity.

**Table 2:** Correlations between stromal SFRP2 expression and other important clinicopathological parameters in urothelial carcinomas.

Parameter	Category	Upper Urinary Tract Urothelial Carcinoma				Urinary Bladder Urothelial Carcinoma			
		Case No.	Stromal SFRP2 Exp.		*p*-value	Case No.	Stromal SFRP2 Exp.		*p*-value
			Low	High			Low	High	
Gender	Male	158	76	82	0.514	216	103	113	0.223
	Female	182	94	88		79	44	35	
Age (years)	< 65	138	75	63	0.185	121	60	61	0.944
	≥ 65	202	95	107		174	87	87	
Tumor location	Renal pelvis	141	60	81	0.069	–	–	–	–
	Ureter	150	83	67		–	–	–	–
	Renal pelvis & ureter	49	27	22		–	–	–	–
Multifocality	Single	278	136	142	0.399	–	–	–	–
	Multifocal	62	34	28		–	–	–	–
Primary tumor (T)	Ta	89	64	25	<0.001*	84	60	24	<0.001*
	T1	92	55	37		88	39	49	
	T2-T4	159	51	108		123	48	75	
Nodal metastasis	Negative (N0)	312	165	147	<0.001*	266	139	127	0.012*
	Positive (N1-N2)	28	5	23		29	8	21	
Histological grade	Low grade	56	43	13	<0.001*	56	39	17	0.001*
	High grade	284	127	157		239	108	131	
Vascular invasion	Absent	234	144	90	<0.001*	246	132	114	0.003*
	Present	106	26	80		49	15	34	
Perineural invasion	Absent	321	165	156	0.034*	275	143	132	0.006*
	Present	19	5	14		20	4	16	
Mitotic rate (per 10 high power fields)	< 10	173	115	58	<0.001*	139	89	50	<0.001*
	>= 10	167	55	112		156	58	98	
Tumoral SFRP2 expression	High	170	115	55	<0.001*	148	104	44	<0.001*
	Low	170	55	115		147	43	104	

*Statistically significant.

The bold values were considered statistically significant.

### Correlations Among Stromal and Tumoral SFRP2 Immunoexpression and Clinicopathological Parameters

**Figure 3:**
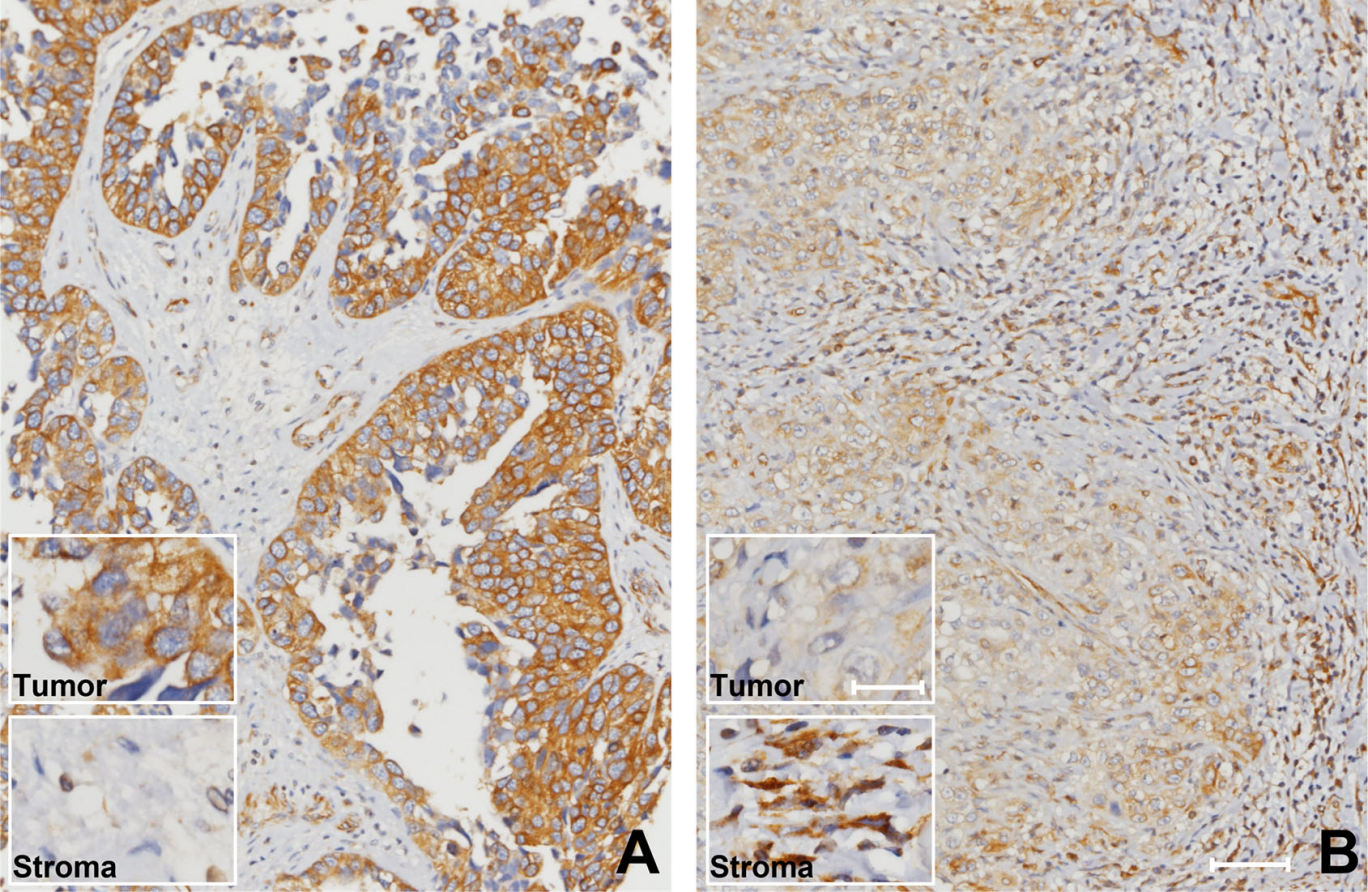
High stromal SFRP2 immunoexpression was observed among bladder cancer patients with advanced stage disease. Immunohistochemistry was performed with an anti-SFRP2 antibody. SFRP2 immunoreactivity in stroma was gradually increased from **(A)** non-muscle-invasive to **(B)** muscle-invasive bladder cancer (200x, scale bar = 100 μm). Insect: 200x, scale bar = 25 μm.

Immunohistochemical staining revealed that SFRP2 immunoreactivity in stroma was progressively increased from early stage to advanced stage UC (**Figures 3A, B**). **Table 2** displays stromal SFRP2 immunoexpression and its clinical significance in UTUC and UBUC. In the UTUC group, high stromal SFRP2 expression was remarkably linked to high tumor stage and histological grade (both *p* < 0.001), positive nodal metastasis (*p* < 0.001), the presence of vascular and perineural invasion (*p* < 0.001 and *p* = 0.034), high mitotic activity (*p* < 0.001), and low tumoral SFRP2 expression (*p* < 0.001). Likewise, in the UBUC group, we observed notable correlations between high stromal SFRP2 expression and high tumor stage and histological grade (*p* < 0.001 and *p* = 0.001), positive nodal metastasis (*p* = 0.012), the presence of vascular and perineural invasion (*p* = 0.003 and *p* = 0.006), high mitotic activity (*p* < 0.001), and low tumoral SFRP2 expression (*p* < 0.001).

In contrast, **Table 3** exhibits tumoral SFRP2 immunoexpression and its clinical significance in UTUC and UBUC. Interestingly, in the UTUC group, low tumoral SFRP2 expression was remarkably linked to high histological grade (*p* = 0.041) and positive nodal metastasis (*p* = 0.006). In the UBUC group, we detected remarkable correlations between low tumoral SFRP2 expression and high tumor stage (*p* = 0.004), the presence of vascular invasion (*p* = 0.039), and high mitotic activity (*p* = 0.002). Accordingly, the disparate roles of SFRP2 seem to be dependent on cell type-specific contexts.

**Table 3:** Correlations between tumoral SFRP2 expression and other important clinicopathological parameters in urothelial carcinomas.

Parameter	Category	Upper Urinary Tract Urothelial Carcinoma				Urinary Bladder Urothelial Carcinoma			
		Case No.	Tumoral SFRP2 Exp.		*p*-value	Case No.	Tumoral SFRP2 Exp.		*p*-value
			Low	High			Low	High	
Gender	Male	158	74	84	0.277	216	112	104	0.251
	Female	182	96	86		79	35	44	
Age (years)	< 65	138	63	75	0.185	121	68	53	0.068
	≥ 65	202	107	95		174	79	95	
Tumor location	Renal pelvis	141	72	69	0.784	–	–	–	–
	Ureter	150	72	78		–	–	–	–
	Renal pelvis & ureter	49	26	23		–	–	–	–
Multifocality	Single	278	138	140	0.779	–	–	–	–
	Multifocal	62	32	30		–	–	–	–
Primary tumor (T)	Ta	89	37	52	0.083	84	31	53	0.004*
	T1	92	44	48		88	42	46	
	T2-T4	159	89	70		123	74	49	
Nodal metastasis	Negative (N0)	312	149	163	0.006*	266	130	136	0.319
	Positive (N1-N2)	28	21	7		29	17	12	
Histological grade	Low grade	56	21	35	0.041*	56	23	33	0.145
	High grade	284	149	135		239	124	115	
Vascular invasion	Absent	234	113	121	0.349	246	116	130	0.039*
	Present	106	57	49		49	31	18	
Perineural invasion	Absent	321	161	160	0.813	275	133	142	0.062
	Present	19	9	10		20	14	6	
Mitotic rate (per 10 high power fields)	< 10	173	81	92	0.233	139	56	83	0.002*
	>= 10	167	89	78		156	91	65	
Stromal SFRP2 expression	Low	170	55	115	<0.001*	147	43	104	<0.001*
	High	170	115	55		148	104	44	

*Statistically significant.

The bold values were considered statistically significant.

### Survival Analysis and Prognostic Impact of SFRP2 Expression in Stroma and Tumors

**Figure 4:**
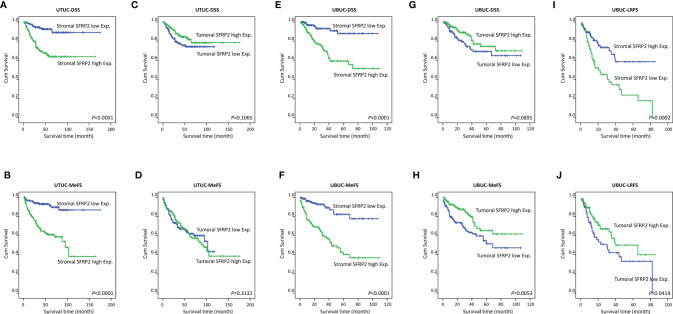
Survival analysis. Kaplan–Meier curves show that high stromal SFRP2 immunoexpression conferred unfavorable prognostic effects on disease-specific survival and metastasis-free survival in **(A–D)** UTUC and **(E, F)** UBUC patients. Low tumoral SFRP2 expression was adversely prognostic only for metastasis-free survival in **(G, H)** UBUC patients. Also, high SFRP2 immunoexpression in stroma and low SFRP2 immunoexpression in tumors were significantly correlated with poor local recurrence-free survival in **(I, J)** UBUC patients.

There were 113 deaths owing to UC, including 61 patients with UTUC and 52 patients with UBUC, in our cohorts. Additionally, a total of 146 patients, including 70 with UTUC and 76 with UBUC, had following distal metastasis. To appraise the prognostic implications of SFRP2 expression in patient death and distal metastasis in UC, univariate and multivariate analyses were utilized. In respect of UTUC, high SFRP2 expression in stroma but not in tumors was unfavorably prognostic for both disease-specific survival (DSS) and metastasis-free survival (MeFS) (both *p* < 0.0001) in the univariate analysis (**Table 4** and **Figures 4A–D**). Also, multifocal tumors, high tumor stage and histological grade, positive nodal metastasis, and vascular and perineural invasion significantly conferred poor outcomes in both DSS and MeFS (all *p* < 0.0215). Following multivariate analysis, stroma with high SFRP2 expression and multifocality, positive nodal metastasis, and perineural invasion remained independently prognostic for poor DSS and MeFS (all *p* < 0.035). These results imply that high SFRP2 expression in stroma instead of tumors can act as an adverse prognostic indicator for UTUC patients.

**Table 4:** Univariate log-rank and multivariate analyses for disease-specific and metastasis-free survivals in upper urinary tract urothelial carcinoma.

Parameter	Category	Case No.	Disease-specific Survival					Metastasis-free Survival				
			Univariate analysis		Multivariate analysis			Univariate analysis		Multivariate analysis		
			No. of event	*p*-value	R.R.	95% C.I.	*p*-value	No. of event	*p*-value	R.R.	95% C.I.	*p*-value
**Gender**	Male	158	28	0.8286	–	–	–	32	0.7904	–	–	–
	Female	182	33		–	–	–	38		–	–	–
**Age (years)**	< 65	138	26	0.9943	–	–	–	30	0.8470	–	–	–
	≥ 65	202	35		–	–	–	40		–	–	–
**Tumor side**	Right	177	34	0.7366	–	–	–	38	0.3074	–	–	–
	Left	154	26		–	–	–	32		–	–	–
	Bilateral	9	1		–	–	–	0		–	–	–
**Tumor location**	Renal pelvis	141	24	0.0079*	1	–	0.997	31	0.0659	–	–	–
	Ureter	150	22		0.888	0.480-1.645		25		–	–	–
	Renal pelvis & ureter	49	15		1.348	0.375-4.841		14		–	–	–
**Multifocality**	Single	273	48	0.0026*	1	–	0.011*	52	0.0127*	1	–	0.004*
	Multifocal	62	18		2.239	0.665-7.535		18		2.260	1.298-3.933	
**Primary tumor (T)**	Ta	89	2	<0.0001*	1	–	0.051	4	<0.0001*	1	–	0.110
	T1	92	9		3.667	0.786-17.104		15		3.295	1.078-10.075	
	T2-T4	159	50		4.768	1.062-21.399		51		2.497	0.790-7.892	
**Nodal metastasis**	Negative (N0)	312	42	<0.0001*	1	–	<0.001*	**55**	<0.0001*	1	–	0.002*
	Positive (N1-N2)	28	19		4.937	2.662-9.156		**15**		2.627	1.411-4.891	
**Histological grade**	Low grade	56	4	0.0215*	1	–	0.009*	3	0.0027*	1	–	0.080
	High grade	284	57		3.661	1.343-9.9775		67		2.023	0.919-4.454	
**Vascular invasion**	Absent	234	24	<0.0001*	1	–	0.362	26	<0.0001*	1	–	0.012*
	Present	106	37		1.315	0.710-2.435		44		2.192	1.191-4.035	
**Perineural invasion**	Absent	321	50	<0.0001*	1	–	<0.001*	61	<0.0001*	1	–	0.011*
	Present	19	11		4.109	1.949-8.665		9		2.647	1.245-5.630	
**Mitotic rate (per 10 high power fields)**	< 10	173	27	0.167	–	–		30	0.0823	–	–	
	>= 10	167	34		–	–		40		–	–	
**Stromal SFRP2 expression**	Low	170	12	<0.0001*	1	–	0.035*	13	<0.0001*	1	–	0.001*
	High	170	49		2.361	1.205-4.627		57		3.039	1.597-5.785	
**Tumoral SFRP2 expression**	Low	170	36	0.1065	–	–		38	0.3132	–	–	
	High	170	25		–	–		32		–	–	

*Statistically significant.

The bold values were considered statistically significant.

In terms of UBUC, high SFRP2 expression in stroma was unfavorably prognostic for both DSS and MeFS (both *p* < 0.0001) following univariate analysis (**Table 5** and **Figures 4E, F**). In contrast, high SFRP2 expression in tumors was a favorable prognostic indicator and its prognostic effect was only on MeFS (*p* = 0.0053) (**Figures 4G, H**). In addition, high tumor stage and histological grade, positive nodal metastasis, vascular and perineural invasion, and high mitotic activity also significantly conferred worse outcomes in both DSS and MeFS (all *p* < 0.0024). In the multivariate analysis, stroma with high SFRP2 expression and high tumor stage were significantly prognostic for inferior DSS and MeFS (all *p* < 0.001). If NMIBC patients receiving TURBT develop local recurrence, radical cystectomy will be further performed, which may reduce quality of life. Accordingly, we also found that high stromal SFRP2 expression, low tumoral SFRP2 expression, and high tumor stage and histological grade were significantly correlated with poor local recurrence-free survival (LRFS) (all *p* < 0.0414) in the univariate analysis (**Table 6** and **Figures 4I, J**). Furthermore, only stroma with high SFRP2 expression remained independently prognostic for inferior LRFS (*p* = 0.012) following multivariate analysis. Taken together, these results suggest that the prognostic effects of stromal and tumoral SFRP2 expression are distinct, and incorporation of these variables can more accurately guide management for UBUC patients.

**Table 5:** Univariate log-rank and multivariate analyses for disease-specific and metastasis-free survivals in urinary bladder urothelial carcinoma.

Parameter	Category	Case No.	Disease-specific Survival					Metastasis-free Survival				
			Univariate analysis		Multivariate analysis			Univariate analysis		Multivariate analysis		
			No. of event	*p*-value	R.R.	95% C.I.	*p*-value	No. of event	*p*-value	R.R.	95% C.I.	*p*-value
**Gender**	Male	216	41	0.4446	–	**-**	**-**	60	0.2720	–	–	–
	Female	79	11		–	**-**	**-**	16		–	–	–
**Age (years)**	< 65	121	17	0.1136	–	**-**	**-**	31	0.6875	–	–	–
	≥ 65	174	35		–	**-**	**-**	45		–	–	–
**Primary tumor (T)**	Ta	84	1	<0.0001*	1	–	<0.001*	4	<0.0001*	1	–	<0.001*
	T1	88	9		7.007	0.711-69.038		23		1.201	1.201-14.358	
	T2-T4	123	42		35.904	3.718-346.745		49		14.358	2.102-24.092	
**Nodal metastasis**	Negative (N0)	266	41	0.0002*	1	–	0.743	61	<0.0001*	1	–	0.177
	Positive (N1-N2)	29	11		1.124	0.559-2.260		15		1.521	0.827-2.796	
**Histological grade**	Low grade	56	2	0.0013*	1	–	0.739	5	0.0007*	1	–	1,000
	High grade	239	50		0.765	0.159-3.688		71		1.000	0.337-2.967	
**Vascular invasion**	Absent	246	37	0.0024*	1	–	0.060	54	0.0001*	1	–	0.543
	Present	49	15		0.513	0.26-1.029		22		0.832	0.459-1.507	
**Perineural invasion**	Absent	275	44	0.0001*	1	–	0.173	66	0.0007*	1	–	0.508
	Present	20	8		1.792	0.773-4.151		10		1.286	0.611-2.704	
**Mitotic rate (per 10 high power fields)**	< 10	139	12	<0.0001*	1	–	0.066	23	<0.0001*	1	–	0.106
	>= 10	156	40		1.888	0.959-3.719		53		1.539	0.912-2.598	
**Stromal SFRP2 expression**	Low	147	9	<0.0001*	1	–	0.001*	15	<0.0001*	1	–	<0.001*
	High	148	43		3.849	1.791-8.269		61		3.788	2.066-6.946	
**Tumoral SFRP2 expression**	Low	147	31	0.0895	–	–	**-**	48	**0.0053**	1	–	0.418
	High	148	21		–	–	**-**	28		0.819	0.505-1.328	

*Statistically significant.

The bold values were considered statistically significant.

**Table 6:** Univariate log-rank and multivariate analyses for local recurrence-free survivals in NMIBC post TURBT.

Parameter	Category	Case No.	Local Recurrence-free Survival				
			Univariate analysis		Multivariate analysis		
			No. of event	*p*-value	R.R.	95% C.I.	*p*-value
**Gender**	Male	125	46	0.3370	–	**-**	**-**
	Female	47	19		–	**-**	**-**
**Age (years)**	< 65	70	30	0.3857	–	**-**	**-**
	≥ 65	102	35		–	**-**	**-**
**Primary tumor (T)**	Ta	84	27	0.0193*	1	–	0.623
	T1	88	38		1.107	0.629-2.169	
**Nodal metastasis**	Negative (N0)	172	65	n.a.	–	–	–
	Positive (N1-N2)	0	0		–	–	–
**Histological grade**	Low grade	54	15	0.0101*	1	–	0.165
	High grade	118	50		1.650	0.808-3.494	
**Vascular invasion**	Absent	171	65	0.6639	–	–	–
	Present	1	0		–	–	–
**Perineural invasion**	Absent	169	64	0.4725	–	–	–
	Present	3	1		–	–	–
**Mitotic rate (per 10 high power fields)**	< 10	94	35	0.1853	–	–	–
	>= 10	78	30		–	–	–
**Stromal SFRP2 expression**	Low	99	24	0.0002*	1	–	0.012*
	High	73	41		2.083	1.179-3.681	
**Tumoral SFRP2 expression**	Low	73	34	0.0414*	1	–	0.558
	High	99	31		0.805	0.493-1.464	

*Statistically significant; n.a., not applicable.

The bold values were considered statistically significant.

### SFRP2 Function Prediction and Its Link to UC Progression

**Figure 5:**
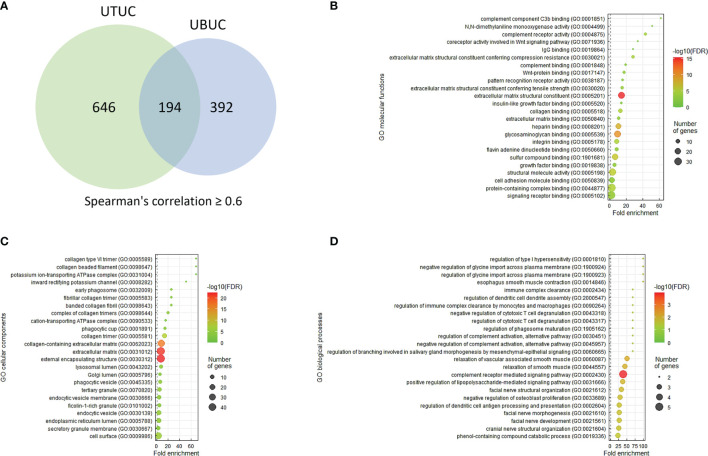
The prominent GO terms enriched in *SFRP2* upregulation. **(A)** The top 194 overlapping genes (Spearman’s correlation ≥ 0.6) co-upregulated with *SFRP2* between UTUC and UBUC were examined utilizing the GO classification system according to **(B)** molecular functions, **(C)** cellular components, or **(D)** biological processes and were ranked by fold enrichment for functional annotation. To plot representative GO terms, an R script with ggplot2 package was used.

**Figure 6:**
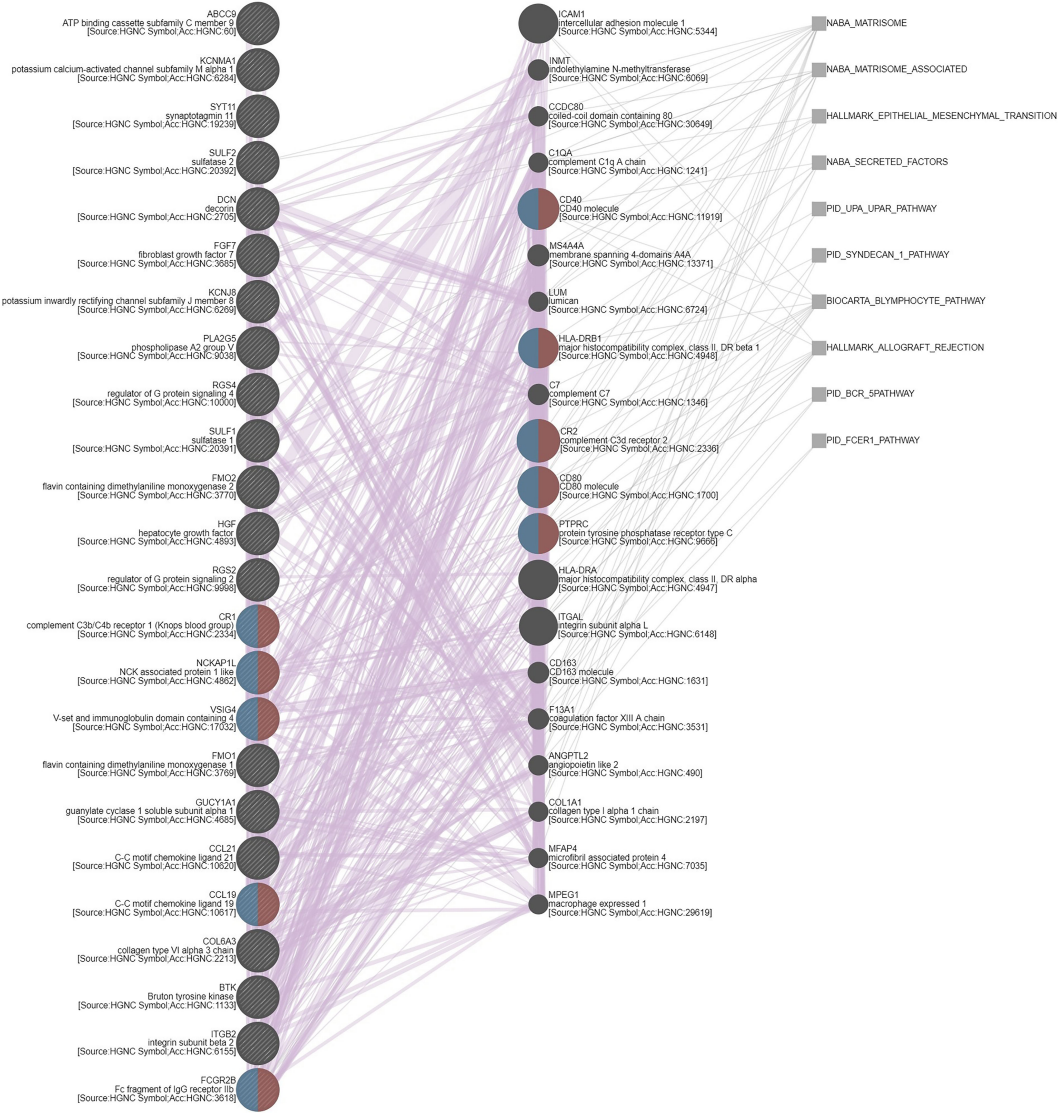
Gene coexpression network. A weighted network that connects key genes to each other was built using the GeneMANIA prediction server. Red and blue semicircles represent genes that belong to mononuclear cell proliferation and lymphocyte proliferation, respectively. Purple and grey lines indicate coregulation and pathways, correspondingly.

UTUC and UBUC are featured by etiological and clinical heterogeneity, while we identified SFRP2 linked to similar prognosis among UTUC and UBUC patients. To understand the functional roles of SFRP2 in UC, a gene coexpression network was examined. Employing the UTUC (*n* = 47) and UBUC (*n* = 411) datasets in the TCGA database, we appraised the top 194 overlapping transcripts (**Figure 5A**) that show positive correlations with *SFRP2* between UTUC (**Supplementary Table 1**) and UBUC (**Supplementary Table 2**). Subsequently, we utilized the GO classification system for functional annotation. With regard to molecular functions, we found that these overlapping genes are mainly implicated in the composition of the ECM (**Figure 5B**). In respect of cellular components, we observed that these overlapping genes are mostly involved in the collagen trimer assembly (**Figure 5C**). Moreover, the fibulin 2 (*FBLN2*) gene, one of the top 194 overlapping genes co-upregulated with *SFRP2* (**Supplementary Figure 3A**), has been identified as an unfavorable prognostic factor for UC in our previous study (29). Interestingly, the collagen family genes are also significantly positively correlated with *FBLN2* (29), further supporting the important role of collagen in UC development. As to biological processes, we detected that these overlapping genes are largely implicated in the immune cell regulation (**Figure 5D**). Furthermore, expressed by macrophages, the cathepsin K (*CTSK*) gene, one of the top 194 overlapping genes co-upregulated with *SFRP2* (**Supplementary Figure 3B**), has been suggested to be a promising therapeutic target for patients with high-risk MIBC (30). To connect key genes that were involved in the distinguished GO terms (fold enrichment > 50) of all three functional groups to each other, a weighted network was built using the GeneMANIA prediction server (31). The data showed that the top 2 predicted functions are mononuclear cell proliferation (false discovery rate: 1.85 x 10^−7^) and lymphocyte proliferation (false discovery rate: 1.85 x 10^−7^) (**Figure 6**). Additionally, annotated by the Molecular Signatures Database (MSigDB), several prominent pathways, including matrisome, epithelial–mesenchymal transition (EMT), and B lymphocyte, were identified. These observations further support the role of SFRP2 in the crosstalk among tumor cells, stromal cells, immune cells, and the ECM.

Furthermore, *SFRP4* was also identified as one of the overlapping genes considerably co-upregulated with *SFRP2* (**Supplementary Figure 3C**). It has been reported that *SFRP2* and *SFRP4* levels are tightly correlated with each other, which shares a common gene program across multiple cancers (19). Using the Human Protein Atlas database, in terms of single cell type specificity, we found that *SFRP2* is specifically expressed in the fibroblasts (https://www.proteinatlas.org/ENSG00000145423-SFRP2/celltype), but *SFRP4* is specifically expressed in the peritubular cells (https://www.proteinatlas.org/ENSG00000106483-SFRP4/celltype), suggesting that the contribution of tumor stroma to UC development is more likely to be mediated by SFRP2. We also identified fibroblast growth factor 7 (*FGF7*) and complement C1s (*C1S*) as the overlapping transcripts that are significantly positively correlated with *SFRP2* (**Supplementary Figures 3D, E**). In our previous investigations, high FGF7 (32) and C1S (33) expression have been correlated with worse clinical outcomes in UC patients. Altogether, although UTUC and UBUC are clinically managed as distinct entities, they share common genomic landscape with similar actionable drivers and prognostic factors to improve precision therapy.

## Discussion

Composed of five secreted glycoproteins (SFRP1–5), the SFRP family is approximately 300 amino acids in length, which fold into two distinguishable domains: an N-terminal cysteine-rich domain and a C-terminal netrin domain (34). Because of their ability to antagonize the Wnt signaling and their frequent silencing by promoter methylation in many cancers, the SFRP proteins were initially described as tumor suppressors (35). Actually, one study found that the methylation level of *SFRP2* in gastric cancer is higher than that in adjacent normal tissue (36), whereas a recent report showed that the mRNA level of *SFRP2* is among the highest in advanced stages of gastric cancer and correlated with worse survival (37). Likewise, using the UALCAN platform (http://ualcan.path.uab.edu/cgi-bin/TCGA-methyl-Result.pl?genenam=SFRP1&ctype=BLCA) (http://ualcan.path.uab.edu/cgi-bin/TCGA-methyl-Result.pl?genenam=SFRP2&ctype=BLCA), we speculate that, compared to adjacent normal tissue, both the decreased *SFRP1* and *SFRP2* transcripts in bladder cancer may ascribe to their increased methylation levels, especially in patients with stage I disease. Interestingly, the violin plots showed that both the mRNA levels of *SFRP1* and *SFRP2* significantly increase with the progression of bladder cancer (from stage II to stage IV) (**Supplementary Figure 1A** and **Figure 2A**). These observed inconsistencies in the level of *SFRP2* might be owing to differences in the progressive stage. However, we observed that both the *SFRP1* and *SFRP2* mRNA levels are not inversely correlated with their methylation status using the TCGA bladder cancer database (*n* = 413) (**Supplementary Figures 4A, B**). This implies that alternative mechanisms may contribute to increased *SFRP1* and *SFRP2* transcripts in UC patients with advanced disease.

The dynamic interplays between tumors and their microenvironment comprising immune cells (macrophages and lymphocytes), stromal cells (fibroblasts), and the ECM, supporting or limiting tumor growth. To further dissect the molecular characterization of tumor–immune interactions, the TIMER2.0 database was used. The data revealed that high CAF infiltration was significantly correlated with poor cumulative survival in bladder urothelial carcinoma (**Supplementary Figures 5A, B**). Moreover, we observed that both the *SFRP1* and *SFRP2* transcripts are significantly negatively correlated with tumor purity and positively correlated with CAF infiltration in bladder urothelial carcinoma, but the *SFRP2* gene showed a more distinguished correlation (**Supplementary Figure 2** and **Figure 2C**). These observations suggest that stromal cells may at least in part result in the increased *SFRP2* transcripts in urothelial carcinoma with advanced stage. We further validated that high stromal SFRP2 expression evaluated by immunohistochemistry is significantly linked to an aggressive clinical course and inferior survival in our urothelial carcinoma cohorts containing 340 UTUC and 295 UBUC patients, highlighting the promising prognostic utility of stromal SFRP2 expression.

Initially regarded as transcriptional noise, non-coding RNAs (ncRNAs) have attracted wide attention for their involvement in epigenetic regulation and multiple biological functions, especially in cancer (38). Based on the length, ncRNAs can majorly be classified into microRNAs (miRNAs, about 22 nucleotides long) and long non-coding RNAs (lncRNAs, more than 200 nucleotides long) (39). miRNAs function by annealing to the three prime untranslated region (3′-UTR) of messenger RNA (mRNA) targets to negatively regulate gene expression. As competitive endogenous RNAs (ceRNAs) or miRNA sponges, lncRNAs with miRNA-complementary sites, which are also presented on mRNA targets, can compete with mRNA targets to bind to miRNAs, thereby reducing the availability of miRNAs. Accordingly, we aligned GSE31684 probe sequences with human lncRNA sequences from the LNCipedia database (https://lncipedia.org/) and created a lncRNA list. Utilizing the miRTarBase database (40), the regulatory networks among miRNAs, upstream regulators (lncRNA list), and downstream targets (gene list from **Table 1**) were analyzed, and the ZNF585B-6:1/miRNAs/SFRP2 network was identified (**Supplementary Figure 6**). Of these miRNAs, miR-218 has been reported to be downregulated in bladder cancer (41), which suggests that increased *SFRP2* transcripts may attribute to miR-218 downregulation in UC. Consequently, we do not rule out the possibility that ncRNAs may contribute to increased *SFRP2* transcripts in UC, and further analysis is needed.

A gene coexpression analysis was used to predict the functional roles of SFRP2 in UC, and we found that the collagen family genes, including collagen type I alpha 2 chain (*COL1A2*), *COL3A1*, *COL6A3*, *COL8A1*, *COL10A1*, and *COL14A1*, are significantly co-upregulated with *SFRP2* (**Supplementary Figures 7A–F**). These collagen genes, such as *COL1A2* and *COL6A3*, have been demonstrated to promote NMIBC progression to MIBC through EMT (42). We also found that EMT markers twist family basic helix-loop-helix (bHLH) transcription factor 1 (*TWIST1*) and zinc finger E-box binding homeobox 2 (*ZEB2*) are significantly co-upregulated with *SFRP2* (**Supplementary Figures 8A, B**). However, whether SFRP2 promotes UC progression through EMT regulated by the above-mentioned collagen genes needs further confirmation. On the other hand, although the use of immunotherapy has improved outcomes in the management of UC, only approximately 20% of patients benefit from it (43), which warrants a better understanding of the mechanisms underlying immunotherapy resistance. It has been reported that the transforming growth factor beta (TGFβ) signaling from fibroblasts and collagen enriched in peritumoral stroma promote cytotoxic T cell exclusion and confer immunotherapy resistance among patients with metastatic urothelial cancer (44). Additionally, we also observed that *TGFBI* and CAF marker fibroblast activation protein (*FAP*) are significantly co-upregulated with *SFRP2* (**Supplementary Figures 9A, B**). Accordingly, these observations suggest that SFRP2, a putative Wnt inhibitor, may function at the interactions between tumor cells and fibroblasts in UC development. Nevertheless, the Wnt/β-catenin signaling has also been suggested to drive cytotoxic T cell exclusion by modulating the crosstalk between tumor cells and tumor-associated macrophages (TAMs) and lead to immunotherapy resistance in UC treatment (12). These further highlight the complexity of the TME that creates a favorable niche to reduce treatment efficacy. Accordingly, SFRP2 may trigger immunotherapy resistance beyond its Wnt antagonistic ability, providing therapeutic implications for precisely selecting patients who can benefit from immunotherapy.

Increased deposition of fibrous collagen is the most common feature of ECM remodeling in the primary tumor (45). Despite the fact that the collagen family genes are significantly positively correlated with *SFRP2*, it is unclear how SFRP2 regulates collagen homeostasis. Using structure prediction analysis, it has been showed that the C-terminal netrin domains of SFRP proteins and procollagen C-endopeptidase enhancer 1 (PCPE1 or PCOLCE) share sequence similarity with the N-terminal netrin domains of tissue inhibitor of metalloproteinases (TIMPs) (46). PCPE1 is suggested to be highly specific to collagen synthesis and fibrosis, and its upregulation is observed at sites of high collagen deposition (47). Moreover, TIMPs are considered to inhibit matrix metalloproteinases (MMPs)-induced collagen degradation (48). However, whether SFRP2 can promote collagen synthesis and prevent collagen degradation through its C-terminal netrin domain requires further analysis. Otherwise, due to its sensitivity to inflammatory perturbations, variation of collagen stainability may be of little utility in respect of the prognosis of UC (49). Consequently, SFRP2, a supposed upstream regulator of collagen homeostasis, may act as a reliable prognostic factor for UC.

Metastatic UC is an aggressive disease and generally has a poor prognosis with a median overall survival of 12–14 months (4). Following lymph nodes (69%), bone (47%) has been reported to be the second most common site of metastatic bladder cancer (50). Bone metastasis may be correlated with pain, bone loss, and functional impairment. Accordingly, recent study has reported that UC patients with bone as the only metastatic site are less likely to receive systemic chemotherapy owing to their lower Eastern Cooperative Oncology Group (ECOG) performance status (51). The current European Association of Urology (EAU) guideline recommends anti-osteoporotic drugs (zoledronic acid or denosumab) for supportive treatment in case of bone metastasis, but patients should be aware of potential side effects, such as osteonecrosis of the jaw and hypocalcaemia (4). As a result, these patients deserve a valuable prognostic factor and a specific therapeutic target. Using gene coexpression analysis, we found that *CTSK* is significantly co-upregulated with *SFRP2* (**Supplementary Figure 3B**). Generally, CTSK, a lysosomal cysteine protease, is implicated in osteoclast-mediated bone degradation (52). Moreover, CTSK has also been described to be expressed by breast cancer (53) and prostate cancer (54) that metastasize to bone, where it functions in osteolysis that contributes to tumor invasiveness. Nevertheless, the correlations among the expression of SFRP2 and CTSK and metastatic bone disease in UC need further examination.

In this study, we identified that high stromal SFRP2 expression has a more significant impact on UC patient survival compared with that of low tumoral SFRP2 expression. This suggests that the distinct roles of SFRP2 seem to be dependent on cell type-specific contexts, and incorporation of these variables can more accurately guide management for UC patients. The current study has some limitations. First, further experiments are needed to validate the role of fibroblast SFRP2 in UC development and immunotherapy resistance. Second, in this study, UC patients who had curative surgery were analyzed retrospectively at a single institution; accordingly, the value of stromal SFRP2 expression should be prospectively verified by multi-center studies.

## Conclusion

Because of their Wnt antagonistic ability and their frequent epigenetic silencing in many cancers, the SFRP proteins were initially described as tumor suppressors. In the current study, we validated that high stromal SFRP2 expression is considerably correlated with an aggressive clinical course and serves as an independent prognostic factor for worse survival in our well-characterized UBUC and UTUC cohorts. Utilizing bioinformatic analysis, we further observed that stromal SFRP2 may link EMT to UC progression. Collectively, stromal SFRP2 may exert oncogenic function beyond its Wnt antagonistic ability, and stromal SFRP2 expression evaluation can add value to guiding management more precisely for UC patients.

## Data Availability Statement

The datasets presented in this study can be found in online repositories. The names of the repository/repositories and accession number(s) can be found in the article/**Supplementary Material**.

## Ethics Statement

The studies involving human participants were reviewed and approved by Institutional Review Board of Chi Mei Medical Center (10501005). The patients/participants provided their written informed consent to participate in this study.

## Author Contributions

Conceptualization: H-YL and C-FL. Methodology: C-CC, Y-HK, H-HT, L-CW, W-HT, C-LL, and C-HH. Investigation: H-YL, C-CC, SH, and C-FL. Formal analysis: H-YL, C-CC, Y-HK, H-HT, and L-CW. Resources: W-HT, C-LL, and C-HH. Validation: Y-HK, H-HT, L-CW, W-HT, C-LL, and C-HH. Visualization: H-YL and C-CC. Writing - original draft: H-YL. Writing - review and editing: H-YL. Funding acquisition: SH and C-FL. Supervision: SH and C-FL. All authors contributed to the article and approved the submitted version.

## Conflict of Interest

Author C-CC is employed by Genetics Generation Advancement Corp.

The remaining authors declare that the research was conducted in the absence of any commercial or financial relationships that could be construed as a potential conflict of interest.

## Publisher’s Note

All claims expressed in this article are solely those of the authors and do not necessarily represent those of their affiliated organizations, or those of the publisher, the editors and the reviewers. Any product that may be evaluated in this article, or claim that may be made by its manufacturer, is not guaranteed or endorsed by the publisher.

## Supplementary Material

Supplementary Figure 1 High *SFRP1* mRNA level is correlated with advanced stage disease but not with inferior overall survival. **(A)** The correlations between the mRNA levels of *SFRP1* and bladder cancer progression. **(B)** The impact of *SFRP1* mRNA levels on overall survival in bladder cancer. These data were acquired from the GEPIA database. BLCA: bladder urothelial carcinoma.

Supplementary Figure 2
*SFRP1* transcripts are moderately negatively correlated with tumor purity and positively correlated with CAF infiltration. The correlations among the mRNA levels of *SFRP1*, tumor purity, and CAF infiltration in bladder cancer. These data were estimated using the MCP-COUNTER and TIDE algorithms from the TIMER2.0 database. BLCA, bladder urothelial carcinoma.

Supplementary Figure 3 Correlations between the expression levels of *SFRP2* and its co-upregulated genes. **(A–E)** Utilizing the cBioPortal web platform, these data were obtained from the TCGA database (*n* = 411).

Supplementary Figure 4 Both the mRNA levels of *SFRP1* and *SFRP2* are not negatively correlated with their methylation status. **(A)** The correlations between the mRNA levels of *SFRP1* and their methylation status. **(B)** The correlations between the mRNA levels of *SFRP2* and their methylation status. Utilizing the cBioPortal web platform, these data were obtained from the TCGA database (*n* = 413).

Supplementary Figure 5 High CAF infiltration is significantly correlated with poor cumulative survival. **(A, B)** The impact of CAF infiltration on cumulative survival in bladder cancer. These data were estimated using the MCP-COUNTER and TIDE algorithms from the TIMER2.0 database.

Supplementary Figure 6 Regulatory networks among *ZNF585B-6:1*, miRNAs, and *SFRP2*. These data were obtained from the miRTarBase database.

Supplementary Figure 7 Correlations between the expression levels of *SFRP2* and collagen family genes. **(A–F)** Utilizing the cBioPortal web platform, these data were obtained from the TCGA database (*n* = 411).

Supplementary Figure 8 Correlations between the expression levels of *SFRP2* and EMT markers. **(A, B)** Utilizing the cBioPortal web platform, these data were obtained from the TCGA database (*n* = 411).

Supplementary Figure 9 Correlations between the expression levels of *SFRP2* and *TGFB1* and CAF marker. **(A, B)** Utilizing the cBioPortal web platform, these data were obtained from the TCGA database (*n* = 411).
