# Supplementary material for: High Stromal SFRP2 Expression in Urothelial Carcinoma Confers an Unfavorable Prognosis
Source: Front Oncol. 2022 Mar 16;12:834249. doi: 10.3389/fonc.2022.834249 (PMC8965759; doi:10.3389/fonc.2022.834249)
Supplement: Supplementary file 1 [file DataSheet_1.docx]

**Suppl. Table 1. The top 840 genes positively correlated with SFRP2 (Spearman's correlation ≥ 0.6) in UTUC.**

| **Correlated Gene** | **Cytoband** | **Spearman's Correlation** | **p-Value** | **q-Value** |
| --- | --- | --- | --- | --- |
| **CD79A** | 19q13.2 | 0.855 | 4.88E-10 | **8.86E-06** |
| **MZB1** | 5q31.2 | 0.838 | 2.17E-09 | **1.97E-05** |
| **CCDC80** | 3q13.2 | 0.827 | 5.54E-09 | **2.74E-05** |
| **GREM1** | 15q13.3 | 0.826 | 6.03E-09 | **2.74E-05** |
| **KCNMA1** | 10q22.3 | 0.804 | 3.01E-08 | **1.09E-04** |
| **FGF7** | 15q21.2 | 0.787 | 9.07E-08 | **1.91E-04** |
| **AOX1** | 2q33.1 | 0.787 | 9.30E-08 | **1.91E-04** |
| **IGLL5** | 22q11.22 | 0.786 | 9.73E-08 | **1.91E-04** |
| **PDLIM3** | 4q35.1 | 0.783 | 1.19E-07 | **1.91E-04** |
| **PI16** | 6p21.2 | 0.781 | 1.30E-07 | **1.91E-04** |
| **TNFAIP8L3** | 15q21.2 | 0.781 | 1.32E-07 | **1.91E-04** |
| **JCHAIN** | 4q13.3 | 0.781 | 1.32E-07 | **1.91E-04** |
| **POSTN** | 13q13.3 | 0.781 | 1.37E-07 | **1.91E-04** |
| **CCN4** | 8q24.22 | 0.776 | 1.77E-07 | **2.30E-04** |
| **SFRP4** | 7p14.1 | 0.772 | 2.29E-07 | **2.77E-04** |
| **MGP** | 12p12.3 | 0.769 | 2.77E-07 | **2.90E-04** |
| **PRRX1** | 1q24.2 | 0.768 | 2.83E-07 | **2.90E-04** |
| **VCAM1** | 1p21.2 | 0.767 | 3.11E-07 | **2.90E-04** |
| **BEND6** | 6p12.1 | 0.767 | 3.12E-07 | **2.90E-04** |
| **EFEMP1** | 2p16.1 | 0.766 | 3.23E-07 | **2.90E-04** |
| **CD180** | 5q12.3 | 0.765 | 3.48E-07 | **2.90E-04** |
| **LYVE1** | 11p15.4 | 0.764 | 3.53E-07 | **2.90E-04** |
| **DCLK1** | 13q13.3 | 0.764 | 3.71E-07 | **2.90E-04** |
| **CD84** | 1q23.3 | 0.763 | 3.83E-07 | **2.90E-04** |
| **CCL2** | 17q12 | 0.762 | 4.14E-07 | **3.01E-04** |
| **CPXM2** | 10q26.13 | 0.76 | 4.46E-07 | **3.12E-04** |
| **GEM** | 8q22.1 | 0.76 | 4.65E-07 | **3.13E-04** |
| **DCN** | 12q21.33 | 0.759 | 4.89E-07 | **3.17E-04** |
| **FBN1** | 15q21.1 | 0.755 | 5.97E-07 | **3.66E-04** |
| **S1PR3** | 9q22.1 | 0.754 | 6.33E-07 | **3.66E-04** |
| **LAX1** | 1q32.1 | 0.753 | 6.47E-07 | **3.66E-04** |
| **PLCL1** | 2q33.1 | 0.753 | 6.62E-07 | **3.66E-04** |
| **CD38** | 4p15.32 | 0.753 | 6.73E-07 | **3.66E-04** |
| **LUM** | 12q21.33 | 0.752 | 6.84E-07 | **3.66E-04** |
| **SERPINA3** | 14q32.13 | 0.75 | 7.56E-07 | **3.87E-04** |
| **CLEC4E** | 12p13.31 | 0.75 | 7.67E-07 | **3.87E-04** |
| **IL6** | 7p15.3 | 0.748 | 8.39E-07 | **4.10E-04** |
| **TMEM156** | 4p14 | 0.748 | 8.57E-07 | **4.10E-04** |
| **BCAT1** | 12p12.1 | 0.747 | 8.94E-07 | **4.17E-04** |
| **TNFSF8** | 9q32-q33.1 | 0.745 | 1.02E-06 | **4.59E-04** |
| **CLIC4** | 1p36.11 | 0.744 | 1.08E-06 | **4.59E-04** |
| **PHF20** | 20q11.22-q11.23 | 0.743 | 1.10E-06 | **4.59E-04** |
| **FCRL1** | 1q23.1 | 0.743 | 1.11E-06 | **4.59E-04** |
| **MS4A1** | 11q12.2 | 0.742 | 1.17E-06 | **4.59E-04** |
| **MOXD1** | 6q23.2 | 0.742 | 1.17E-06 | **4.59E-04** |
| **SULF1** | 8q13.2-q13.3 | 0.742 | 1.20E-06 | **4.59E-04** |
| **DOCK11** | Xq24 | 0.741 | 1.20E-06 | **4.59E-04** |
| **IRF4** | 6p25.3 | 0.741 | 1.21E-06 | **4.59E-04** |
| **RGS4** | 1q23.3 | 0.739 | 1.34E-06 | **4.96E-04** |
| **ALDH1L2** | 12q23.3 | 0.738 | 1.41E-06 | **5.11E-04** |
| **FYB1** | 5p13.1 | 0.738 | 1.44E-06 | **5.12E-04** |
| **KLHL6** | 3q27.1 | 0.736 | 1.56E-06 | **5.41E-04** |
| **NNMT** | 11q23.2 | 0.736 | 1.59E-06 | **5.41E-04** |
| **CHRDL1** | Xq23 | 0.735 | 1.63E-06 | **5.41E-04** |
| **EBF1** | 5q33.3 | 0.735 | 1.65E-06 | **5.41E-04** |
| **SFRP1** | 8p11.21 | 0.734 | 1.73E-06 | **5.41E-04** |
| **CXCL2** | 4q13.3 | 0.734 | 1.73E-06 | **5.41E-04** |
| **CELF2** | 10p14 | 0.734 | 1.75E-06 | **5.41E-04** |
| **ADH1B** | 4q23 | 0.734 | 1.78E-06 | **5.41E-04** |
| **SLC47A1** | 17p11.2 | 0.733 | 1.81E-06 | **5.41E-04** |
| **NTM** | 11q25 | 0.733 | 1.84E-06 | **5.41E-04** |
| **PLXNA4** | 7q32.3 | 0.733 | 1.86E-06 | **5.41E-04** |
| **CHSY1** | 15q26.3 | 0.733 | 1.87E-06 | **5.41E-04** |
| **DENND5B** | 12p11.21 | 0.732 | 1.96E-06 | **5.54E-04** |
| **COLEC12** | 18p11.32 | 0.731 | 1.98E-06 | **5.54E-04** |
| **THUMPD3** | 3p25.3 | 0.731 | 2.01E-06 | **5.54E-04** |
| **PAPPA** | 9q33.1 | 0.73 | 2.15E-06 | **5.80E-04** |
| **SAMSN1** | 21q11.2 | 0.729 | 2.18E-06 | **5.80E-04** |
| **ADAMTS5** | 21q21.3 | 0.729 | 2.20E-06 | **5.80E-04** |
| **CSF2RB** | 22q12.3 | 0.729 | 2.27E-06 | **5.89E-04** |
| **CDH11** | 16q21 | 0.728 | 2.33E-06 | **5.91E-04** |
| **SLAMF7** | 1q23.3 | 0.728 | 2.34E-06 | **5.91E-04** |
| **FCRL5** | 1q23.1 | 0.725 | 2.73E-06 | **6.65E-04** |
| **ANGPT1** | 8q23.1 | 0.725 | 2.75E-06 | **6.65E-04** |
| **CD36** | 7q21.11 | 0.724 | 2.77E-06 | **6.65E-04** |
| **CXORF21** | Xp21.2 | 0.724 | 2.82E-06 | **6.65E-04** |
| **CCL19** | 9p13.3 | 0.724 | 2.87E-06 | **6.65E-04** |
| **EVI2A** | 17q11.2 | 0.724 | 2.88E-06 | **6.65E-04** |
| **SYT11** | 1q22 | 0.723 | 2.89E-06 | **6.65E-04** |
| **MYLK** | 3q21.1 | 0.723 | 2.95E-06 | **6.71E-04** |
| **CD28** | 2q33.2 | 0.723 | 3.00E-06 | **6.73E-04** |
| **GFPT2** | 5q35.3 | 0.722 | 3.08E-06 | **6.73E-04** |
| **IRF8** | 16q24.1 | 0.722 | 3.11E-06 | **6.73E-04** |
| **ABCD2** | 12q12 | 0.722 | 3.13E-06 | **6.73E-04** |
| **BCL2A1** | 15q25.1 | 0.721 | 3.21E-06 | **6.73E-04** |
| **CLIC2** | Xq28 | 0.721 | 3.26E-06 | **6.73E-04** |
| **TENT5C** | 1p12 | 0.721 | 3.31E-06 | **6.73E-04** |
| **C9ORF47** | 9q22.1 | 0.72 | 3.36E-06 | **6.73E-04** |
| **TCTEX1D1** | 1p31.3 | 0.72 | 3.36E-06 | **6.73E-04** |
| **RAD1** | 5p13.2 | 0.72 | 3.38E-06 | **6.73E-04** |
| **BTLA** | 3q13.2 | 0.72 | 3.40E-06 | **6.73E-04** |
| **GLIPR2** | 9p13.3 | 0.72 | 3.46E-06 | **6.73E-04** |
| **RIPOR2** | 6p22.3 | 0.719 | 3.48E-06 | **6.73E-04** |
| **IKZF1** | 7p12.2 | 0.719 | 3.48E-06 | **6.73E-04** |
| **CALHM6** | 6q22.1 | 0.719 | 3.56E-06 | **6.81E-04** |
| **C5AR1** | 19q13.32 | 0.719 | 3.62E-06 | **6.86E-04** |
| **KLHL5** | 4p14 | 0.718 | 3.68E-06 | **6.89E-04** |
| **GAS1** | 9q21.33 | 0.716 | 4.00E-06 | **7.42E-04** |
| **SH2D1A** | Xq25 | 0.716 | 4.05E-06 | **7.42E-04** |
| **FIBIN** | 11p14.2 | 0.716 | 4.13E-06 | **7.42E-04** |
| **IQGAP2** | 5q13.3 | 0.716 | 4.16E-06 | **7.42E-04** |
| **MS4A4A** | 11q12.2 | 0.715 | 4.18E-06 | **7.42E-04** |
| **TLR8** | Xp22.2 | 0.715 | 4.20E-06 | **7.42E-04** |
| **CXCR4** | 2q22.1 | 0.715 | 4.27E-06 | **7.47E-04** |
| **CENPL** | 1q25.1 | 0.714 | 4.49E-06 | **7.77E-04** |
| **PTPRC** | 1q31.3-q32.1 | 0.713 | 4.59E-06 | **7.87E-04** |
| **CHRDL2** | 11q13.4 | 0.712 | 4.84E-06 | **8.16E-04** |
| **SLA** | 8q24.22 | 0.712 | 4.85E-06 | **8.16E-04** |
| **IL7R** | 5p13.2 | 0.711 | 5.02E-06 | **8.38E-04** |
| **TGFBI** | 5q31.1 | 0.711 | 5.12E-06 | **8.46E-04** |
| **FOXD1** | 5q13.2 | 0.71 | 5.22E-06 | **8.55E-04** |
| **MTHFD2** | 2p13.1 | 0.709 | 5.46E-06 | **8.87E-04** |
| **AMMECR1** | Xq23 | 0.709 | 5.55E-06 | **8.93E-04** |
| **SDS** | 12q24.13 | 0.709 | 5.64E-06 | **8.97E-04** |
| **PDE4B** | 1p31.3 | 0.709 | 5.69E-06 | **8.97E-04** |
| **GPR171** | 3q25.1 | 0.708 | 5.74E-06 | **8.97E-04** |
| **MCOLN2** | 1p22.3 | 0.708 | 5.77E-06 | **8.97E-04** |
| **ADAMTS12** | 5p13.3-p13.2 | 0.707 | 6.10E-06 | **9.38E-04** |
| **PDCD1LG2** | 9p24.1 | 0.707 | 6.14E-06 | **9.38E-04** |
| **CYTIP** | 2q24.1 | 0.706 | 6.34E-06 | **9.55E-04** |
| **RGS1** | 1q31.2 | 0.706 | 6.36E-06 | **9.55E-04** |
| **FCGR2B** | 1q23.3 | 0.706 | 6.41E-06 | **9.55E-04** |
| **KIFAP3** | 1q24.2 | 0.705 | 6.52E-06 | **9.63E-04** |
| **EPGN** | 4q13.3 | 0.705 | 6.76E-06 | **9.91E-04** |
| **SRGN** | 10q22.1 | 0.704 | 6.84E-06 | **9.94E-04** |
| **SLC2A3** | 12p13.31 | 0.704 | 6.89E-06 | **9.94E-04** |
| **SLC39A8** | 4q24 | 0.703 | 7.24E-06 | **1.04E-03** |
| **APBB1IP** | 10p12.1 | 0.702 | 7.49E-06 | **1.04E-03** |
| **SNAP25** | 20p12.2 | 0.702 | 7.50E-06 | **1.04E-03** |
| **NFATC2** | 20q13.2 | 0.702 | 7.51E-06 | **1.04E-03** |
| **TIMP2** | 17q25.3 | 0.702 | 7.57E-06 | **1.04E-03** |
| **MS4A7** | 11q12.2 | 0.702 | 7.57E-06 | **1.04E-03** |
| **PTP4A1** | 6q12 | 0.702 | 7.61E-06 | **1.04E-03** |
| **FBLN2** | 3p25.1 | 0.699 | 8.49E-06 | **1.15E-03** |
| **CCR2** | 3p21.31 | 0.699 | 8.70E-06 | **1.16E-03** |
| **CD53** | 1p13.3 | 0.699 | 8.71E-06 | **1.16E-03** |
| **TLR10** | 4p14 | 0.698 | 8.77E-06 | **1.16E-03** |
| **PNOC** | 8p21.1 | 0.698 | 8.79E-06 | **1.16E-03** |
| **FGFR1** | 8p11.23 | 0.698 | 8.85E-06 | **1.16E-03** |
| **PRELP** | 1q32.1 | 0.697 | 9.36E-06 | **1.22E-03** |
| **PLEK** | 2p14 | 0.697 | 9.50E-06 | **1.23E-03** |
| **COPA** | 1q23.2 | 0.696 | 9.63E-06 | **1.23E-03** |
| **GPR183** | 13q32.3 | 0.696 | 9.90E-06 | **1.25E-03** |
| **TNIP3** | 4q27 | 0.695 | 9.98E-06 | **1.25E-03** |
| **INSIG1** | 7q36.3 | 0.695 | 1.00E-05 | **1.25E-03** |
| **PTGER2** | 14q22.1 | 0.695 | 1.01E-05 | **1.25E-03** |
| **RECK** | 9p13.3 | 0.695 | 1.03E-05 | **1.27E-03** |
| **TXLNB** | 6q24.1 | 0.695 | 1.03E-05 | **1.27E-03** |
| **UBXN2A** | 2p23.3 | 0.694 | 1.05E-05 | **1.28E-03** |
| **CD163** | 12p13.31 | 0.694 | 1.07E-05 | **1.29E-03** |
| **LAMP5** | 20p12.2 | 0.693 | 1.09E-05 | **1.31E-03** |
| **MAP3K13** | 3q27.2 | 0.693 | 1.10E-05 | **1.31E-03** |
| **DNAJC5B** | 8q13.1 | 0.693 | 1.10E-05 | **1.31E-03** |
| **PPP2R5E** | 14q23.2 | 0.693 | 1.12E-05 | **1.31E-03** |
| **PLS3** | Xq23 | 0.693 | 1.12E-05 | **1.31E-03** |
| **CALU** | 7q32.1 | 0.692 | 1.13E-05 | **1.31E-03** |
| **ICOS** | 2q33.2 | 0.692 | 1.14E-05 | **1.31E-03** |
| **SCIMP** | 17p13.2 | 0.692 | 1.14E-05 | **1.31E-03** |
| **DES** | 2q35 | 0.692 | 1.15E-05 | **1.31E-03** |
| **FCRL3** | 1q23.1 | 0.692 | 1.15E-05 | **1.31E-03** |
| **HEPH** | Xq12 | 0.692 | 1.17E-05 | **1.32E-03** |
| **RBM24** | 6p22.3 | 0.691 | 1.18E-05 | **1.32E-03** |
| **MSR1** | 8p22 | 0.691 | 1.21E-05 | **1.34E-03** |
| **GNB4** | 3q26.33 | 0.691 | 1.22E-05 | **1.34E-03** |
| **ST6GAL1** | 3q27.3 | 0.691 | 1.22E-05 | **1.34E-03** |
| **SPN** | 16p11.2 | 0.691 | 1.22E-05 | **1.34E-03** |
| **CLEC17A** | 19p13.12 | 0.69 | 1.23E-05 | **1.34E-03** |
| **USP13** | 3q26.33 | 0.689 | 1.28E-05 | **1.37E-03** |
| **F2R** | 5q13.3 | 0.689 | 1.28E-05 | **1.37E-03** |
| **LRRC58** | 3q13.33 | 0.689 | 1.28E-05 | **1.37E-03** |
| **LBP** | 20q11.23 | 0.689 | 1.29E-05 | **1.38E-03** |
| **MSRB3** | 12q14.3 | 0.689 | 1.30E-05 | **1.38E-03** |
| **CC2D2A** | 4p15.32 | 0.688 | 1.33E-05 | **1.39E-03** |
| **PIK3AP1** | 10q24.1 | 0.688 | 1.34E-05 | **1.40E-03** |
| **HSD11B1** | 1q32.2 | 0.688 | 1.35E-05 | **1.40E-03** |
| **HSPA13** | 21q11.2 | 0.688 | 1.35E-05 | **1.40E-03** |
| **C7** | 5p13.1 | 0.687 | 1.39E-05 | **1.41E-03** |
| **FILIP1L** | 3q12.1 | 0.687 | 1.39E-05 | **1.41E-03** |
| **AKAP5** | 14q23.3 | 0.687 | 1.40E-05 | **1.41E-03** |
| **CXCL13** | 4q21.1 | 0.687 | 1.40E-05 | **1.41E-03** |
| **FCRL2** | 1q23.1 | 0.687 | 1.41E-05 | **1.41E-03** |
| **SLC16A1** | 1p13.2 | 0.687 | 1.42E-05 | **1.42E-03** |
| **HRH2** | 5q35.2 | 0.686 | 1.47E-05 | **1.45E-03** |
| **BTK** | Xq22.1 | 0.686 | 1.47E-05 | **1.45E-03** |
| **GZMK** | 5q11.2 | 0.686 | 1.48E-05 | **1.45E-03** |
| **SAV1** | 14q22.1 | 0.686 | 1.49E-05 | **1.46E-03** |
| **EIF2AK2** | 2p22.2 | 0.685 | 1.50E-05 | **1.46E-03** |
| **WIPF1** | 2q31.1 | 0.685 | 1.52E-05 | **1.47E-03** |
| **SCRG1** | 4q34.1 | 0.684 | 1.56E-05 | **1.50E-03** |
| **COL6A3** | 2q37.3 | 0.684 | 1.59E-05 | **1.52E-03** |
| **ACTC1** | 15q14 | 0.684 | 1.59E-05 | **1.52E-03** |
| **GPNMB** | 7p15.3 | 0.684 | 1.60E-05 | **1.52E-03** |
| **CHODL** | 21q21.1 | 0.683 | 1.64E-05 | **1.53E-03** |
| **TFEC** | 7q31.2 | 0.683 | 1.65E-05 | **1.53E-03** |
| **C1S** | 12p13.31 | 0.683 | 1.66E-05 | **1.53E-03** |
| **SGCE** | 7q21.3 | 0.683 | 1.67E-05 | **1.53E-03** |
| **TAF13** | 1p13.3 | 0.683 | 1.67E-05 | **1.53E-03** |
| **AMPD1** | 1p13.2 | 0.683 | 1.67E-05 | **1.53E-03** |
| **PAK2** | 3q29 | 0.682 | 1.69E-05 | **1.54E-03** |
| **ZNF426** | 19p13.2 | 0.681 | 1.77E-05 | **1.61E-03** |
| **STAP1** | 4q13.2 | 0.681 | 1.79E-05 | **1.61E-03** |
| **ALPK2** | 18q21.31-q21.32 | 0.681 | 1.79E-05 | **1.61E-03** |
| **TNFRSF17** | 16p13.13 | 0.681 | 1.80E-05 | **1.61E-03** |
| **CPT1A** | 11q13.3 | 0.679 | 1.91E-05 | **1.69E-03** |
| **IL2RA** | 10p15.1 | 0.679 | 1.91E-05 | **1.69E-03** |
| **FAM177B** | 1q41 | 0.679 | 1.93E-05 | **1.71E-03** |
| **SERPINB9** | 6p25.2 | 0.679 | 1.96E-05 | **1.72E-03** |
| **TGM2** | 20q11.23 | 0.678 | 1.99E-05 | **1.73E-03** |
| **IL21R** | 16p12.1 | 0.678 | 2.01E-05 | **1.73E-03** |
| **CD22** | 19q13.12 | 0.678 | 2.01E-05 | **1.73E-03** |
| **ISLR** | 15q24.1 | 0.678 | 2.02E-05 | **1.73E-03** |
| **DYRK3** | 1q32.1 | 0.678 | 2.02E-05 | **1.73E-03** |
| **CNTN1** | 12q12 | 0.677 | 2.08E-05 | **1.78E-03** |
| **ZNF850** | 19q13.12 | 0.677 | 2.10E-05 | **1.79E-03** |
| **TAGAP** | 6q25.3 | 0.676 | 2.17E-05 | **1.84E-03** |
| **CR1** | 1q32.2 | 0.676 | 2.18E-05 | **1.84E-03** |
| **TNFRSF9** | 1p36.23 | 0.676 | 2.21E-05 | **1.85E-03** |
| **MTMR12** | 5p13.3 | 0.676 | 2.21E-05 | **1.85E-03** |
| **MMD** | 17q22 | 0.675 | 2.23E-05 | **1.85E-03** |
| **PIGM** | 1q23.2 | 0.675 | 2.26E-05 | **1.86E-03** |
| **CMSS1** | 3q12.1 | 0.675 | 2.26E-05 | **1.86E-03** |
| **CD48** | 1q23.3 | 0.675 | 2.27E-05 | **1.86E-03** |
| **GNL3L** | Xp11.22 | 0.674 | 2.31E-05 | **1.87E-03** |
| **CCDC117** | 22q12.1 | 0.674 | 2.31E-05 | **1.87E-03** |
| **LY96** | 8q21.11 | 0.674 | 2.32E-05 | **1.87E-03** |
| **FBXO45** | 3q29 | 0.674 | 2.33E-05 | **1.87E-03** |
| **RAB33A** | Xq26.1 | 0.674 | 2.33E-05 | **1.87E-03** |
| **VIM** | 10p13 | 0.674 | 2.36E-05 | **1.87E-03** |
| **COL3A1** | 2q32.2 | 0.674 | 2.36E-05 | **1.87E-03** |
| **CR2** | 1q32.2 | 0.673 | 2.43E-05 | **1.91E-03** |
| **ITK** | 5q33.3 | 0.673 | 2.43E-05 | **1.91E-03** |
| **ST8SIA1** | 12p12.1 | 0.673 | 2.44E-05 | **1.91E-03** |
| **C16ORF89** | 16p13.3 | 0.673 | 2.46E-05 | **1.91E-03** |
| **PTPN22** | 1p13.2 | 0.673 | 2.47E-05 | **1.91E-03** |
| **ATL3** | 11q13.1 | 0.672 | 2.53E-05 | **1.96E-03** |
| **POLR2K** | 8q22.2 | 0.672 | 2.55E-05 | **1.96E-03** |
| **MYADM** | 19q13.42 | 0.672 | 2.58E-05 | **1.98E-03** |
| **TNFAIP6** | 2q23.3 | 0.671 | 2.59E-05 | **1.98E-03** |
| **CD69** | 12p13.31 | 0.671 | 2.61E-05 | **1.98E-03** |
| **ZNF831** | 20q13.32 | 0.671 | 2.61E-05 | **1.98E-03** |
| **LATS2** | 13q12.11 | 0.671 | 2.62E-05 | **1.98E-03** |
| **P2RY10** | Xq21.1 | 0.671 | 2.65E-05 | **1.99E-03** |
| **SNX10** | 7p15.2 | 0.67 | 2.69E-05 | **2.00E-03** |
| **RP2** | Xp11.3 | 0.67 | 2.69E-05 | **2.00E-03** |
| **CALD1** | 7q33 | 0.67 | 2.69E-05 | **2.00E-03** |
| **PDZRN3** | 3p13 | 0.67 | 2.76E-05 | **2.04E-03** |
| **CCNI** | 4q21.1 | 0.669 | 2.79E-05 | **2.04E-03** |
| **RRAGC** | 1p34.3 | 0.669 | 2.79E-05 | **2.04E-03** |
| **IL10RA** | 11q23.3 | 0.669 | 2.79E-05 | **2.04E-03** |
| **HDAC9** | 7p21.1 | 0.669 | 2.85E-05 | **2.07E-03** |
| **JAKMIP1** | 4p16.1 | 0.669 | 2.87E-05 | **2.07E-03** |
| **HLA-DRA** | 6p21.32 | 0.669 | 2.89E-05 | **2.07E-03** |
| **CD37** | 19q13.33 | 0.669 | 2.89E-05 | **2.07E-03** |
| **CD109** | 6q13 | 0.668 | 2.91E-05 | **2.08E-03** |
| **PDE1A** | 2q32.1 | 0.668 | 2.92E-05 | **2.08E-03** |
| **HLA-DOA** | 6p21.32 | 0.668 | 2.95E-05 | **2.09E-03** |
| **RUBCNL** | 13q14.13 | 0.668 | 2.99E-05 | **2.12E-03** |
| **ITGA4** | 2q31.3 | 0.667 | 3.04E-05 | **2.12E-03** |
| **ATP8B2** | 1q21.3 | 0.667 | 3.04E-05 | **2.12E-03** |
| **TIMM8A** | Xq22.1 | 0.667 | 3.05E-05 | **2.12E-03** |
| **PIGX** | 3q29 | 0.667 | 3.05E-05 | **2.12E-03** |
| **STK17B** | 2q32.3 | 0.667 | 3.05E-05 | **2.12E-03** |
| **SLIT2** | 4p15.31 | 0.667 | 3.07E-05 | **2.12E-03** |
| **GLT8D2** | 12q23.3 | 0.667 | 3.09E-05 | **2.13E-03** |
| **HLA-DMB** | 6p21.32 | 0.666 | 3.18E-05 | **2.18E-03** |
| **NCKAP1L** | 12q13.13-q13.2 | 0.666 | 3.20E-05 | **2.18E-03** |
| **NUGGC** | 8p21.1 | 0.665 | 3.24E-05 | **2.21E-03** |
| **CD8A** | 2p11.2 | 0.665 | 3.27E-05 | **2.21E-03** |
| **GUCY1A1** | 4q32.1 | 0.665 | 3.28E-05 | **2.21E-03** |
| **FAP** | 2q24.2 | 0.665 | 3.30E-05 | **2.22E-03** |
| **MT1G** | 16q13 | 0.665 | 3.30E-05 | **2.22E-03** |
| **ITM2A** | Xq21.1 | 0.665 | 3.34E-05 | **2.23E-03** |
| **SPON1** | 11p15.2 | 0.664 | 3.36E-05 | **2.24E-03** |
| **MMP16** | 8q21.3 | 0.664 | 3.37E-05 | **2.24E-03** |
| **RASGRP1** | 15q14 | 0.664 | 3.48E-05 | **2.30E-03** |
| **DOCK10** | 2q36.2 | 0.663 | 3.49E-05 | **2.30E-03** |
| **FMOD** | 1q32.1 | 0.663 | 3.50E-05 | **2.30E-03** |
| **ADAP2** | 17q11.2 | 0.663 | 3.51E-05 | **2.30E-03** |
| **FCRLA** | 1q23.3 | 0.663 | 3.57E-05 | **2.32E-03** |
| **CRTAM** | 11q24.1 | 0.663 | 3.58E-05 | **2.32E-03** |
| **ZMAT3** | 3q26.32 | 0.663 | 3.59E-05 | **2.32E-03** |
| **MAOB** | Xp11.3 | 0.662 | 3.66E-05 | **2.35E-03** |
| **TNFRSF13B** | 17p11.2 | 0.662 | 3.66E-05 | **2.35E-03** |
| **COL4A3** | 2q36.3 | 0.662 | 3.69E-05 | **2.36E-03** |
| **PURB** | 7p13 | 0.662 | 3.71E-05 | **2.37E-03** |
| **RBPJ** | 4p15.2 | 0.662 | 3.74E-05 | **2.37E-03** |
| **SPIB** | 19q13.33 | 0.662 | 3.75E-05 | **2.37E-03** |
| **GIMAP4** | 7q36.1 | 0.661 | 3.77E-05 | **2.38E-03** |
| **PLA2G7** | 6p12.3 | 0.661 | 3.79E-05 | **2.39E-03** |
| **OGFRL1** | 6q13 | 0.661 | 3.87E-05 | **2.43E-03** |
| **COL8A1** | 3q12.1 | 0.661 | 3.88E-05 | **2.43E-03** |
| **FPR2** | 19q13.41 | 0.66 | 3.92E-05 | **2.44E-03** |
| **SLC12A3** | 16q13 | 0.66 | 3.92E-05 | **2.44E-03** |
| **DSTYK** | 1q32.1 | 0.66 | 3.97E-05 | **2.46E-03** |
| **CNR2** | 1p36.11 | 0.66 | 4.00E-05 | **2.46E-03** |
| **MIS18BP1** | 14q21.2 | 0.66 | 4.02E-05 | **2.47E-03** |
| **MLF1** | 3q25.32 | 0.659 | 4.05E-05 | **2.47E-03** |
| **PBRM1** | 3p21.1 | 0.659 | 4.08E-05 | **2.47E-03** |
| **MNDA** | 1q23.1 | 0.659 | 4.09E-05 | **2.47E-03** |
| **SLC1A3** | 5p13.2 | 0.659 | 4.09E-05 | **2.47E-03** |
| **ELOVL4** | 6q14.1 | 0.659 | 4.09E-05 | **2.47E-03** |
| **TMEM41A** | 3q27.2 | 0.659 | 4.16E-05 | **2.50E-03** |
| **DCP1A** | 3p21.1 | 0.659 | 4.18E-05 | **2.50E-03** |
| **NUP155** | 5p13.2 | 0.658 | 4.19E-05 | **2.50E-03** |
| **PLN** | 6q22.31 | 0.658 | 4.23E-05 | **2.51E-03** |
| **PPP1R16B** | 20q11.23 | 0.658 | 4.23E-05 | **2.51E-03** |
| **CPE** | 4q32.3 | 0.658 | 4.23E-05 | **2.51E-03** |
| **ADAMTS3** | 4q13.3 | 0.658 | 4.26E-05 | **2.51E-03** |
| **GHR** | 5p13.1-p12 | 0.658 | 4.26E-05 | **2.51E-03** |
| **SEC24A** | 5q31.1 | 0.658 | 4.32E-05 | **2.53E-03** |
| **ZNF106** | 15q15.1 | 0.658 | 4.33E-05 | **2.53E-03** |
| **KCNA3** | 1p13.3 | 0.657 | 4.35E-05 | **2.54E-03** |
| **VNN1** | 6q23.2 | 0.657 | 4.40E-05 | **2.56E-03** |
| **MPEG1** | 11q12.1 | 0.657 | 4.42E-05 | **2.56E-03** |
| **RNF24** | 20p13 | 0.657 | 4.43E-05 | **2.56E-03** |
| **RESF1** | 12p11.21 | 0.657 | 4.46E-05 | **2.57E-03** |
| **CPED1** | 7q31.31 | 0.656 | 4.50E-05 | **2.58E-03** |
| **LOX** | 5q23.1 | 0.656 | 4.51E-05 | **2.58E-03** |
| **KLRG1** | 12p13.31 | 0.656 | 4.53E-05 | **2.58E-03** |
| **YWHAB** | 20q13.12 | 0.656 | 4.53E-05 | **2.58E-03** |
| **OSBPL11** | 3q21.2 | 0.656 | 4.56E-05 | **2.58E-03** |
| **ANK2** | 4q25-q26 | 0.656 | 4.57E-05 | **2.58E-03** |
| **WDR70** | 5p13.2 | 0.656 | 4.59E-05 | **2.59E-03** |
| **HLTF** | 3q24 | 0.656 | 4.62E-05 | **2.59E-03** |
| **CD86** | 3q13.33 | 0.656 | 4.63E-05 | **2.59E-03** |
| **KPNA4** | 3q25.33 | 0.656 | 4.65E-05 | **2.59E-03** |
| **MTDH** | 8q22.1 | 0.656 | 4.65E-05 | **2.59E-03** |
| **DHX36** | 3q25.2 | 0.655 | 4.78E-05 | **2.64E-03** |
| **CTHRC1** | 8q22.3 | 0.655 | 4.79E-05 | **2.64E-03** |
| **F13A1** | 6p25.1 | 0.655 | 4.80E-05 | **2.64E-03** |
| **CNRIP1** | 2p14 | 0.655 | 4.81E-05 | **2.64E-03** |
| **CD1D** | 1q23.1 | 0.655 | 4.83E-05 | **2.64E-03** |
| **COL14A1** | 8q24.12 | 0.654 | 4.85E-05 | **2.65E-03** |
| **TNFSF13B** | 13q33.3 | 0.654 | 4.87E-05 | **2.65E-03** |
| **HGF** | 7q21.11 | 0.654 | 4.89E-05 | **2.65E-03** |
| **RGL1** | 1q25.3 | 0.654 | 4.93E-05 | **2.66E-03** |
| **CLEC5A** | 7q34 | 0.654 | 4.93E-05 | **2.66E-03** |
| **IREB2** | 15q25.1 | 0.654 | 4.97E-05 | **2.67E-03** |
| **PYGO1** | 15q21.3 | 0.654 | 4.99E-05 | **2.67E-03** |
| **UBA2** | 19q13.11 | 0.653 | 5.07E-05 | **2.71E-03** |
| **GRAP2** | 22q13.1 | 0.653 | 5.13E-05 | **2.72E-03** |
| **FCN1** | 9q34.3 | 0.653 | 5.13E-05 | **2.72E-03** |
| **BLK** | 8p23.1 | 0.653 | 5.13E-05 | **2.72E-03** |
| **NRIP1** | 21q11.2-q21.1 | 0.653 | 5.17E-05 | **2.72E-03** |
| **TIGIT** | 3q13.31 | 0.653 | 5.17E-05 | **2.72E-03** |
| **CD3G** | 11q23.3 | 0.652 | 5.25E-05 | **2.75E-03** |
| **NANP** | 20p11.21 | 0.652 | 5.26E-05 | **2.75E-03** |
| **GLIS3** | 9p24.2 | 0.652 | 5.28E-05 | **2.76E-03** |
| **MFAP4** | 17p11.2 | 0.652 | 5.30E-05 | **2.76E-03** |
| **CRISPLD2** | 16q24.1 | 0.652 | 5.34E-05 | **2.76E-03** |
| **PCDH9** | 13q21.32 | 0.652 | 5.34E-05 | **2.76E-03** |
| **JCAD** | 10p11.23 | 0.652 | 5.35E-05 | **2.76E-03** |
| **ALDH1A1** | 9q21.13 | 0.652 | 5.36E-05 | **2.76E-03** |
| **RAB42** | 1p35.3 | 0.651 | 5.39E-05 | **2.76E-03** |
| **SEPTIN7** | 7p14.2 | 0.651 | 5.41E-05 | **2.76E-03** |
| **P2RX1** | 17p13.2 | 0.651 | 5.41E-05 | **2.76E-03** |
| **TMEM45A** | 3q12.2 | 0.651 | 5.43E-05 | **2.76E-03** |
| **ME1** | 6q14.2 | 0.651 | 5.43E-05 | **2.76E-03** |
| **SLC16A6** | 17q24.2 | 0.651 | 5.47E-05 | **2.77E-03** |
| **STK3** | 8q22.2 | 0.65 | 5.58E-05 | **2.81E-03** |
| **PHEX** | Xp22.11 | 0.65 | 5.61E-05 | **2.81E-03** |
| **ZEB2** | 2q22.3 | 0.65 | 5.62E-05 | **2.81E-03** |
| **EVI2B** | 17q11.2 | 0.65 | 5.62E-05 | **2.81E-03** |
| **LY9** | 1q23.3 | 0.65 | 5.62E-05 | **2.81E-03** |
| **RASGEF1B** | 4q21.21 | 0.65 | 5.67E-05 | **2.82E-03** |
| **RAB22A** | 20q13.32 | 0.649 | 5.77E-05 | **2.86E-03** |
| **ATXN3** | 14q32.12 | 0.649 | 5.79E-05 | **2.87E-03** |
| **CAMK2D** | 4q26 | 0.649 | 5.84E-05 | **2.88E-03** |
| **FOLR2** | 11q13.4 | 0.649 | 5.87E-05 | **2.88E-03** |
| **DCK** | 4q13.3 | 0.649 | 5.87E-05 | **2.88E-03** |
| **GAS7** | 17p13.1 | 0.649 | 5.92E-05 | **2.89E-03** |
| **PYHIN1** | 1q23.1 | 0.649 | 5.94E-05 | **2.89E-03** |
| **CYBB** | Xp21.1-p11.4 | 0.649 | 5.95E-05 | **2.89E-03** |
| **ITGA1** | 5q11.2 | 0.649 | 5.95E-05 | **2.89E-03** |
| **GNG2** | 14q22.1 | 0.648 | 5.98E-05 | **2.90E-03** |
| **RSPO3** | 6q22.33 | 0.648 | 6.01E-05 | **2.90E-03** |
| **NIBAN1** | 1q25.3 | 0.648 | 6.01E-05 | **2.90E-03** |
| **RASSF3** | 12q14.2 | 0.648 | 6.07E-05 | **2.91E-03** |
| **PIM2** | Xp11.23 | 0.648 | 6.07E-05 | **2.91E-03** |
| **DIAPH2** | Xq21.33 | 0.648 | 6.11E-05 | **2.92E-03** |
| **USP14** | 18p11.32 | 0.648 | 6.15E-05 | **2.93E-03** |
| **HAVCR2** | 5q33.3 | 0.648 | 6.15E-05 | **2.93E-03** |
| **ARL4C** | 2q37.1 | 0.648 | 6.17E-05 | **2.93E-03** |
| **ADAMDEC1** | 8p21.2 | 0.647 | 6.22E-05 | **2.94E-03** |
| **MAFB** | 20q12 | 0.647 | 6.27E-05 | **2.96E-03** |
| **SRP72** | 4q12 | 0.647 | 6.31E-05 | **2.97E-03** |
| **SPATA5** | 4q28.1 | 0.647 | 6.39E-05 | **3.00E-03** |
| **ZBTB21** | 21q22.3 | 0.646 | 6.46E-05 | **3.02E-03** |
| **FAM83D** | 20q11.23 | 0.646 | 6.46E-05 | **3.02E-03** |
| **TWIST1** | 7p21.1 | 0.646 | 6.47E-05 | **3.02E-03** |
| **ACAP2** | 3q29 | 0.646 | 6.51E-05 | **3.02E-03** |
| **SSTR2** | 17q25.1 | 0.646 | 6.53E-05 | **3.02E-03** |
| **FAM126A** | 7p15.3 | 0.646 | 6.54E-05 | **3.02E-03** |
| **APOD** | 3q29 | 0.646 | 6.55E-05 | **3.02E-03** |
| **PPP4R2** | 3p13 | 0.646 | 6.59E-05 | **3.03E-03** |
| **CREM** | 10p11.21 | 0.646 | 6.60E-05 | **3.03E-03** |
| **TEX11** | Xq13.1 | 0.646 | 6.62E-05 | **3.03E-03** |
| **RAB23** | 6p12.1-p11.2 | 0.646 | 6.62E-05 | **3.03E-03** |
| **OSBPL8** | 12q21.2 | 0.645 | 6.68E-05 | **3.04E-03** |
| **GPC6** | 13q31.3-q32.1 | 0.645 | 6.73E-05 | **3.05E-03** |
| **ARHGAP29** | 1p22.1 | 0.645 | 6.75E-05 | **3.05E-03** |
| **CD19** | 16p11.2 | 0.645 | 6.75E-05 | **3.05E-03** |
| **COL1A2** | 7q21.3 | 0.645 | 6.76E-05 | **3.05E-03** |
| **RNF115** | 1q21.1 | 0.645 | 6.85E-05 | **3.08E-03** |
| **KDELR3** | 22q13.1 | 0.644 | 6.89E-05 | **3.09E-03** |
| **ZC3H12D** | 6q25.1 | 0.644 | 6.92E-05 | **3.10E-03** |
| **ADGRL2** | 1p31.1 | 0.644 | 6.94E-05 | **3.10E-03** |
| **TRAT1** | 3q13.13 | 0.644 | 6.96E-05 | **3.10E-03** |
| **THEMIS** | 6q22.33 | 0.644 | 7.03E-05 | **3.12E-03** |
| **HTR1F** | 3p11.2-p11.1 | 0.644 | 7.06E-05 | **3.13E-03** |
| **CLEC7A** | 12p13.2 | 0.643 | 7.11E-05 | **3.15E-03** |
| **ZNF148** | 3q21.2 | 0.643 | 7.14E-05 | **3.15E-03** |
| **FPR3** | 19q13.41 | 0.643 | 7.17E-05 | **3.15E-03** |
| **GIMAP1** | 7q36.1 | 0.643 | 7.18E-05 | **3.15E-03** |
| **YIPF4** | 2p22.3 | 0.643 | 7.19E-05 | **3.15E-03** |
| **CCL13** | 17q12 | 0.643 | 7.20E-05 | **3.15E-03** |
| **SLC30A4** | 15q21.1\|15q21.1 | 0.643 | 7.28E-05 | **3.17E-03** |
| **GOLPH3** | 5p13.3 | 0.643 | 7.29E-05 | **3.17E-03** |
| **NPR3** | 5p13.3 | 0.643 | 7.31E-05 | **3.17E-03** |
| **TCEAL7** | Xq22.2 | 0.643 | 7.34E-05 | **3.18E-03** |
| **BCHE** | 3q26.1 | 0.642 | 7.37E-05 | **3.18E-03** |
| **COL10A1** | 6q22.1 | 0.642 | 7.46E-05 | **3.21E-03** |
| **RPS6KA5** | 14q32.11 | 0.642 | 7.46E-05 | **3.21E-03** |
| **TNFSF4** | 1q25.1 | 0.642 | 7.54E-05 | **3.23E-03** |
| **ALOX5AP** | 13q12.3 | 0.642 | 7.57E-05 | **3.23E-03** |
| **SBNO1** | 12q24.31 | 0.642 | 7.57E-05 | **3.23E-03** |
| **MCUB** | 4q25 | 0.641 | 7.63E-05 | **3.25E-03** |
| **PAPSS2** | 10q23.2-q23.31 | 0.641 | 7.68E-05 | **3.26E-03** |
| **SCML4** | 6q21 | 0.641 | 7.73E-05 | **3.28E-03** |
| **OLFML2B** | 1q23.3 | 0.641 | 7.77E-05 | **3.29E-03** |
| **PADI2** | 1p36.13 | 0.641 | 7.80E-05 | **3.29E-03** |
| **ASPN** | 9q22.31 | 0.641 | 7.81E-05 | **3.29E-03** |
| **GPATCH11** | 2p22.2 | 0.641 | 7.85E-05 | **3.29E-03** |
| **IDI1** | 10p15.3 | 0.641 | 7.86E-05 | **3.29E-03** |
| **CYP2S1** | 19q13.2 | 0.641 | 7.86E-05 | **3.29E-03** |
| **ATP6V1A** | 3q13.31 | 0.64 | 7.96E-05 | **3.31E-03** |
| **ANKRD61** | 7p22.1 | 0.64 | 7.96E-05 | **3.31E-03** |
| **FYN** | 6q21 | 0.64 | 8.01E-05 | **3.31E-03** |
| **FPR1** | 19q13.41 | 0.64 | 8.02E-05 | **3.31E-03** |
| **EGR2** | 10q21.3 | 0.64 | 8.03E-05 | **3.31E-03** |
| **RFC1** | 4p14 | 0.64 | 8.06E-05 | **3.31E-03** |
| **TCF4** | 18q21.2 | 0.64 | 8.06E-05 | **3.31E-03** |
| **PRPF40A** | 2q23.3 | 0.64 | 8.11E-05 | **3.32E-03** |
| **MRAS** | 3q22.3 | 0.64 | 8.11E-05 | **3.32E-03** |
| **ATF6** | 1q23.3 | 0.639 | 8.16E-05 | **3.33E-03** |
| **CCL21** | 9p13.3 | 0.639 | 8.26E-05 | **3.36E-03** |
| **TAF1B** | 2p25.1 | 0.639 | 8.26E-05 | **3.36E-03** |
| **CMKLR1** | 12q23.3 | 0.639 | 8.34E-05 | **3.39E-03** |
| **GPX8** | 5q11.2 | 0.639 | 8.36E-05 | **3.39E-03** |
| **ANXA6** | 5q33.1 | 0.638 | 8.47E-05 | **3.41E-03** |
| **IL1R1** | 2q11.2-q12.1 | 0.638 | 8.47E-05 | **3.41E-03** |
| **BAG2** | 6p12.1 | 0.638 | 8.57E-05 | **3.42E-03** |
| **TOP1** | 20q12 | 0.638 | 8.57E-05 | **3.42E-03** |
| **EPRS** | 1q41 | 0.638 | 8.57E-05 | **3.42E-03** |
| **COMMD2** | 3q25.1 | 0.638 | 8.57E-05 | **3.42E-03** |
| **CPA3** | 3q24 | 0.638 | 8.58E-05 | **3.42E-03** |
| **RAP2A** | 13q32.1 | 0.638 | 8.61E-05 | **3.42E-03** |
| **LIN52** | 14q24.3 | 0.638 | 8.65E-05 | **3.43E-03** |
| **CYP1B1** | 2p22.2 | 0.638 | 8.65E-05 | **3.43E-03** |
| **ANKRD55** | 5q11.2 | 0.637 | 8.78E-05 | **3.45E-03** |
| **EIF2S2** | 20q11.22 | 0.637 | 8.79E-05 | **3.45E-03** |
| **FOXN3** | 14q31.3-q32.11 | 0.637 | 8.79E-05 | **3.45E-03** |
| **MYBL1** | 8q13.1 | 0.637 | 8.79E-05 | **3.45E-03** |
| **RASSF9** | 12q21.31 | 0.637 | 8.82E-05 | **3.45E-03** |
| **SMIM10** | Xq26.3 | 0.637 | 8.83E-05 | **3.45E-03** |
| **CD70** | 19p13.3 | 0.637 | 8.88E-05 | **3.46E-03** |
| **MOB1A** | 2p13.1 | 0.637 | 8.90E-05 | **3.46E-03** |
| **MAF** | 16q23.2 | 0.637 | 8.92E-05 | **3.46E-03** |
| **CCDC110** | 4q35.1 | 0.637 | 8.92E-05 | **3.46E-03** |
| **CSE1L** | 20q13.13 | 0.637 | 8.95E-05 | **3.46E-03** |
| **HBS1L** | 6q23.3 | 0.636 | 9.04E-05 | **3.49E-03** |
| **C12ORF75** | 12q23.3 | 0.636 | 9.07E-05 | **3.49E-03** |
| **PPP2R3A** | 3q22.2-q22.3 | 0.636 | 9.10E-05 | **3.50E-03** |
| **KPNA3** | 13q14.2 | 0.636 | 9.12E-05 | **3.50E-03** |
| **OXTR** | 3p25.3 | 0.636 | 9.14E-05 | **3.50E-03** |
| **SEC63** | 6q21 | 0.636 | 9.17E-05 | **3.50E-03** |
| **CALM2** | 2p21 | 0.636 | 9.23E-05 | **3.51E-03** |
| **SULF2** | 20q13.12 | 0.636 | 9.23E-05 | **3.51E-03** |
| **STOM** | 9q33.2 | 0.636 | 9.29E-05 | **3.52E-03** |
| **VSIG4** | Xq12 | 0.635 | 9.31E-05 | **3.52E-03** |
| **WDFY4** | 10q11.23 | 0.635 | 9.31E-05 | **3.52E-03** |
| **DEGS1** | 1q42.11 | 0.635 | 9.33E-05 | **3.52E-03** |
| **NAV3** | 12q21.2 | 0.635 | 9.40E-05 | **3.53E-03** |
| **CD3D** | 11q23.3 | 0.635 | 9.41E-05 | **3.53E-03** |
| **NCF2** | 1q25.3 | 0.635 | 9.46E-05 | **3.55E-03** |
| **ITGB2** | 21q22.3 | 0.634 | 9.63E-05 | **3.60E-03** |
| **PROS1** | 3q11.1 | 0.634 | 9.63E-05 | **3.60E-03** |
| **YIPF6** | Xq12-q13.1 | 0.634 | 9.69E-05 | **3.60E-03** |
| **CNN3** | 1p21.3 | 0.634 | 9.69E-05 | **3.60E-03** |
| **TFRC** | 3q29 | 0.634 | 9.69E-05 | **3.60E-03** |
| **KCNE1** | 21q22.12 | 0.634 | 9.77E-05 | **3.62E-03** |
| **ARHGAP25** | 2p13.3 | 0.634 | 9.79E-05 | **3.62E-03** |
| **TNFSF14** | 19p13.3 | 0.634 | 9.83E-05 | **3.62E-03** |
| **STK4** | 20q13.12 | 0.634 | 9.87E-05 | **3.62E-03** |
| **GPR155** | 2q31.1 | 0.634 | 9.87E-05 | **3.62E-03** |
| **PPAT** | 4q12 | 0.633 | 9.96E-05 | **3.65E-03** |
| **CHST7** | Xp11.3 | 0.633 | 1.01E-04 | **3.68E-03** |
| **GPR34** | Xp11.4 | 0.633 | 1.01E-04 | **3.68E-03** |
| **GPR176** | 15q14-q15.1 | 0.633 | 1.01E-04 | **3.68E-03** |
| **CCN1** | 1p22.3 | 0.633 | 1.01E-04 | **3.68E-03** |
| **RGS2** | 1q31.2 | 0.633 | 1.02E-04 | **3.71E-03** |
| **LTF** | 3p21.31 | 0.632 | 1.03E-04 | **3.72E-03** |
| **NMD3** | 3q26.1 | 0.632 | 1.03E-04 | **3.72E-03** |
| **TOX** | 8q12.1 | 0.632 | 1.03E-04 | **3.73E-03** |
| **KLF9** | 9q21.12 | 0.632 | 1.04E-04 | **3.73E-03** |
| **CYSLTR1** | Xq21.1 | 0.632 | 1.04E-04 | **3.73E-03** |
| **KCNE4** | 2q36.1 | 0.632 | 1.04E-04 | **3.73E-03** |
| **RUNX1T1** | 8q21.3 | 0.632 | 1.05E-04 | **3.76E-03** |
| **PSAT1** | 9q21.2 | 0.632 | 1.06E-04 | **3.76E-03** |
| **SMAP1** | 6q13 | 0.632 | 1.06E-04 | **3.76E-03** |
| **RNASE6** | 14q11.2 | 0.632 | 1.06E-04 | **3.77E-03** |
| **SENP2** | 3q27.2 | 0.632 | 1.06E-04 | **3.77E-03** |
| **SUSD3** | 9q22.31 | 0.631 | 1.06E-04 | **3.77E-03** |
| **BRCA2** | 13q13.1 | 0.631 | 1.07E-04 | **3.78E-03** |
| **IGSF6** | 16p12.2 | 0.631 | 1.07E-04 | **3.78E-03** |
| **CD83** | 6p23 | 0.631 | 1.07E-04 | **3.78E-03** |
| **STYX** | 14q22.1 | 0.631 | 1.08E-04 | **3.79E-03** |
| **KCTD20** | 6p21.31 | 0.631 | 1.08E-04 | **3.79E-03** |
| **NPTX1** | 17q25.3 | 0.631 | 1.08E-04 | **3.79E-03** |
| **MS4A6A** | 11q12.2 | 0.631 | 1.09E-04 | **3.79E-03** |
| **AKT3** | 1q43-q44 | 0.631 | 1.09E-04 | **3.79E-03** |
| **ROR2** | 9q22.31 | 0.631 | 1.09E-04 | **3.81E-03** |
| **CPPED1** | 16p13.12 | 0.63 | 1.10E-04 | **3.83E-03** |
| **CDC40** | 6q21 | 0.63 | 1.11E-04 | **3.86E-03** |
| **FGL2** | 7q11.23 | 0.63 | 1.12E-04 | **3.89E-03** |
| **SLAIN2** | 4p11 | 0.63 | 1.13E-04 | **3.90E-03** |
| **ADGRL4** | 1p31.1 | 0.629 | 1.14E-04 | **3.91E-03** |
| **RAB3GAP2** | 1q41 | 0.629 | 1.14E-04 | **3.91E-03** |
| **PMP22** | 17p12 | 0.629 | 1.14E-04 | **3.91E-03** |
| **EPHA3** | 3p11.1 | 0.629 | 1.14E-04 | **3.91E-03** |
| **PIK3R5** | 17p13.1 | 0.629 | 1.14E-04 | **3.91E-03** |
| **ASH1L** | 1q22 | 0.629 | 1.15E-04 | **3.92E-03** |
| **EGR3** | 8p21.3 | 0.629 | 1.15E-04 | **3.93E-03** |
| **FNIP2** | 4q32.1 | 0.629 | 1.16E-04 | **3.95E-03** |
| **GIMAP7** | 7q36.1 | 0.629 | 1.17E-04 | **3.96E-03** |
| **CD200R1** | 3q13.2 | 0.629 | 1.17E-04 | **3.96E-03** |
| **MS4A6E** | 11q12.2 | 0.629 | 1.17E-04 | **3.96E-03** |
| **PTGFR** | 1p31.1 | 0.629 | 1.17E-04 | **3.96E-03** |
| **CHST11** | 12q23.3 | 0.628 | 1.18E-04 | **3.96E-03** |
| **TXNDC16** | 14q22.1 | 0.628 | 1.18E-04 | **3.96E-03** |
| **NPY1R** | 4q32.2 | 0.628 | 1.18E-04 | **3.97E-03** |
| **AGO2** | 8q24.3 | 0.628 | 1.19E-04 | **3.99E-03** |
| **BACH1** | 21q21.3 | 0.628 | 1.19E-04 | **3.99E-03** |
| **TIPRL** | 1q24.2 | 0.628 | 1.20E-04 | **3.99E-03** |
| **GJC1** | 17q21.31 | 0.628 | 1.20E-04 | **3.99E-03** |
| **TMEM65** | 8q24.13 | 0.628 | 1.20E-04 | **4.00E-03** |
| **ABL2** | 1q25.2 | 0.628 | 1.20E-04 | **4.00E-03** |
| **MORF4L1** | 15q25.1 | 0.628 | 1.21E-04 | **4.00E-03** |
| **FSD1L** | 9q31.2 | 0.628 | 1.21E-04 | **4.00E-03** |
| **CD40LG** | Xq26.3 | 0.627 | 1.22E-04 | **4.02E-03** |
| **PDP2** | 16q22.1 | 0.627 | 1.22E-04 | **4.03E-03** |
| **FAM49A** | 2p24.2 | 0.627 | 1.22E-04 | **4.03E-03** |
| **CPVL** | 7p14.3 | 0.627 | 1.23E-04 | **4.03E-03** |
| **NRP2** | 2q33.3 | 0.627 | 1.24E-04 | **4.07E-03** |
| **EDIL3** | 5q14.3 | 0.627 | 1.24E-04 | **4.07E-03** |
| **RCSD1** | 1q24.2 | 0.627 | 1.25E-04 | **4.09E-03** |
| **ADCY7** | 16q12.1 | 0.626 | 1.25E-04 | **4.09E-03** |
| **SAMHD1** | 20q11.23 | 0.626 | 1.26E-04 | **4.10E-03** |
| **ARHGAP15** | 2q22.2-q22.3 | 0.626 | 1.27E-04 | **4.12E-03** |
| **PRICKLE1** | 12q12 | 0.626 | 1.27E-04 | **4.12E-03** |
| **TGFBR1** | 9q22.33 | 0.626 | 1.28E-04 | **4.12E-03** |
| **SLC25A43** | Xq24 | 0.626 | 1.28E-04 | **4.12E-03** |
| **CD52** | 1p36.11 | 0.626 | 1.28E-04 | **4.12E-03** |
| **SIGLEC10** | 19q13.41 | 0.626 | 1.28E-04 | **4.12E-03** |
| **SPIN4** | Xq11.1 | 0.626 | 1.28E-04 | **4.12E-03** |
| **KIAA1143** | 3p21.31 | 0.626 | 1.29E-04 | **4.12E-03** |
| **MTF1** | 1p34.3 | 0.626 | 1.29E-04 | **4.12E-03** |
| **PLXNC1** | 12q22 | 0.625 | 1.29E-04 | **4.12E-03** |
| **CDCA7** | 2q31.1 | 0.625 | 1.29E-04 | **4.12E-03** |
| **TAGLN** | 11q23.3 | 0.625 | 1.29E-04 | **4.12E-03** |
| **GCNT1** | 9q21.13 | 0.625 | 1.30E-04 | **4.13E-03** |
| **SYNM** | 15q26.3 | 0.625 | 1.30E-04 | **4.14E-03** |
| **CDC42EP3** | 2p22.2 | 0.625 | 1.31E-04 | **4.15E-03** |
| **PLA2G5** | 1p36.13 | 0.625 | 1.32E-04 | **4.15E-03** |
| **CMPK1** | 1p33 | 0.625 | 1.32E-04 | **4.15E-03** |
| **YME1L1** | 10p12.1 | 0.625 | 1.32E-04 | **4.15E-03** |
| **SSTR3** | 22q13.1 | 0.625 | 1.32E-04 | **4.16E-03** |
| **CLEC4G** | 19p13.2 | 0.624 | 1.34E-04 | **4.21E-03** |
| **FLI1** | 11q24.3 | 0.624 | 1.35E-04 | **4.23E-03** |
| **LHFPL6** | 13q13.3-q14.11 | 0.624 | 1.35E-04 | **4.23E-03** |
| **SEPTIN11** | 4q21.1 | 0.624 | 1.35E-04 | **4.23E-03** |
| **CHSY3** | 5q23.3 | 0.624 | 1.35E-04 | **4.23E-03** |
| **ZNF131** | 5p12 | 0.624 | 1.36E-04 | **4.24E-03** |
| **MEDAG** | 13q12.3 | 0.624 | 1.36E-04 | **4.24E-03** |
| **TMOD1** | 9q22.33 | 0.624 | 1.36E-04 | **4.24E-03** |
| **USP42** | 7p22.1 | 0.624 | 1.37E-04 | **4.24E-03** |
| **L2HGDH** | 14q21.3 | 0.624 | 1.38E-04 | **4.26E-03** |
| **DKK2** | 4q25 | 0.623 | 1.38E-04 | **4.26E-03** |
| **CAP2** | 6p22.3 | 0.623 | 1.38E-04 | **4.26E-03** |
| **NLRC3** | 16p13.3 | 0.623 | 1.38E-04 | **4.26E-03** |
| **MCL1** | 1q21.2 | 0.623 | 1.39E-04 | **4.26E-03** |
| **SLC31A1** | 9q32 | 0.623 | 1.39E-04 | **4.26E-03** |
| **CYBRD1** | 2q31.1 | 0.623 | 1.39E-04 | **4.26E-03** |
| **CCN5** | 20q13.12 | 0.623 | 1.40E-04 | **4.28E-03** |
| **PPM1A** | 14q23.1 | 0.623 | 1.40E-04 | **4.29E-03** |
| **ARL5A** | 2q23.3 | 0.623 | 1.41E-04 | **4.29E-03** |
| **MAFG** | 17q25.3 | 0.623 | 1.41E-04 | **4.29E-03** |
| **PANK3** | 5q34 | 0.623 | 1.41E-04 | **4.29E-03** |
| **PIGK** | 1p31.1 | 0.623 | 1.42E-04 | **4.29E-03** |
| **LCP2** | 5q35.1 | 0.623 | 1.42E-04 | **4.29E-03** |
| **DCUN1D3** | 16p12.3 | 0.623 | 1.42E-04 | **4.29E-03** |
| **IPP** | 1p34.1 | 0.622 | 1.43E-04 | **4.32E-03** |
| **ARMCX3** | Xq22.1 | 0.622 | 1.44E-04 | **4.32E-03** |
| **RLF** | 1p34.2 | 0.622 | 1.44E-04 | **4.32E-03** |
| **ATP11C** | Xq27.1 | 0.622 | 1.44E-04 | **4.32E-03** |
| **OGN** | 9q22.31 | 0.622 | 1.46E-04 | **4.38E-03** |
| **FAM180A** | 7q33 | 0.622 | 1.47E-04 | **4.38E-03** |
| **DERL1** | 8q24.13 | 0.621 | 1.47E-04 | **4.38E-03** |
| **MAPRE1** | 20q11.21 | 0.621 | 1.47E-04 | **4.38E-03** |
| **C1D** | 2p14 | 0.621 | 1.47E-04 | **4.38E-03** |
| **PRUNE2** | 9q21.2 | 0.621 | 1.47E-04 | **4.38E-03** |
| **NOD2** | 16q12.1 | 0.621 | 1.49E-04 | **4.44E-03** |
| **ZNRF3** | 22q12.1 | 0.621 | 1.50E-04 | **4.44E-03** |
| **GBP5** | 1p22.2 | 0.621 | 1.51E-04 | **4.47E-03** |
| **PMEPA1** | 20q13.31 | 0.621 | 1.51E-04 | **4.47E-03** |
| **IL12RB2** | 1p31.3 | 0.62 | 1.52E-04 | **4.49E-03** |
| **SIRPA** | 20p13 | 0.62 | 1.53E-04 | **4.50E-03** |
| **MPP1** | Xq28 | 0.62 | 1.53E-04 | **4.50E-03** |
| **GPR174** | Xq21.1 | 0.62 | 1.54E-04 | **4.50E-03** |
| **KCNJ8** | 12p12.1 | 0.62 | 1.54E-04 | **4.50E-03** |
| **SNRNP27** | 2p13.3 | 0.62 | 1.54E-04 | **4.50E-03** |
| **USP1** | 1p31.3 | 0.62 | 1.54E-04 | **4.50E-03** |
| **UBE2QL1** | 5p15.31 | 0.62 | 1.55E-04 | **4.51E-03** |
| **PNMA1** | 14q24.3 | 0.62 | 1.56E-04 | **4.53E-03** |
| **RAB30** | 11q14.1 | 0.62 | 1.56E-04 | **4.53E-03** |
| **WDHD1** | 14q22.2-q22.3 | 0.619 | 1.57E-04 | **4.53E-03** |
| **NOX4** | 11q14.3 | 0.619 | 1.57E-04 | **4.53E-03** |
| **GUCY1B1** | 4q32.1 | 0.619 | 1.57E-04 | **4.53E-03** |
| **CTLA4** | 2q33.2 | 0.619 | 1.57E-04 | **4.53E-03** |
| **SLC25A53** | Xq22.2 | 0.619 | 1.58E-04 | **4.53E-03** |
| **PLSCR4** | 3q24 | 0.619 | 1.58E-04 | **4.53E-03** |
| **RUNX3** | 1p36.11 | 0.619 | 1.58E-04 | **4.53E-03** |
| **NUCKS1** | 1q32.1 | 0.619 | 1.58E-04 | **4.53E-03** |
| **LIFR** | 5p13.1 | 0.619 | 1.58E-04 | **4.53E-03** |
| **LY86** | 6p25.1 | 0.619 | 1.59E-04 | **4.54E-03** |
| **TMCC3** | 12q22 | 0.619 | 1.59E-04 | **4.54E-03** |
| **SUZ12** | 17q11.2 | 0.619 | 1.60E-04 | **4.57E-03** |
| **ZNF704** | 8q21.13 | 0.619 | 1.60E-04 | **4.57E-03** |
| **RBL1** | 20q11.23 | 0.619 | 1.61E-04 | **4.57E-03** |
| **DIO2** | 14q31.1 | 0.619 | 1.61E-04 | **4.57E-03** |
| **NBN** | 8q21.3 | 0.619 | 1.61E-04 | **4.57E-03** |
| **SEC62** | 3q26.2 | 0.618 | 1.62E-04 | **4.57E-03** |
| **FAM172A** | 5q15 | 0.618 | 1.62E-04 | **4.57E-03** |
| **FAM91A1** | 8q24.13 | 0.618 | 1.62E-04 | **4.57E-03** |
| **VSTM2L** | 20q11.23 | 0.618 | 1.62E-04 | **4.57E-03** |
| **SLAMF6** | 1q23.2-q23.3 | 0.618 | 1.63E-04 | **4.57E-03** |
| **UBXN7** | 3q29 | 0.618 | 1.63E-04 | **4.58E-03** |
| **HMGXB4** | 22q12.3 | 0.618 | 1.63E-04 | **4.58E-03** |
| **CD93** | 20p11.21 | 0.618 | 1.64E-04 | **4.59E-03** |
| **TADA1** | 1q24.1 | 0.618 | 1.66E-04 | **4.65E-03** |
| **USP16** | 21q21.3 | 0.617 | 1.68E-04 | **4.68E-03** |
| **TENT5A** | 6q14.1 | 0.617 | 1.68E-04 | **4.68E-03** |
| **S1PR1** | 1p21.2 | 0.617 | 1.69E-04 | **4.69E-03** |
| **THBD** | 20p11.21 | 0.617 | 1.69E-04 | **4.69E-03** |
| **WWTR1** | 3q25.1 | 0.617 | 1.69E-04 | **4.69E-03** |
| **SERINC3** | 20q13.12 | 0.617 | 1.70E-04 | **4.70E-03** |
| **F8** | Xq28 | 0.617 | 1.71E-04 | **4.70E-03** |
| **ENOX2** | Xq26.1 | 0.617 | 1.71E-04 | **4.70E-03** |
| **CTSK** | 1q21.3 | 0.617 | 1.71E-04 | **4.70E-03** |
| **SNRPB2** | 20p12.1 | 0.617 | 1.71E-04 | **4.70E-03** |
| **GGH** | 8q12.3 | 0.617 | 1.71E-04 | **4.70E-03** |
| **SPEF2** | 5p13.2 | 0.617 | 1.71E-04 | **4.70E-03** |
| **AQP9** | 15q21.3 | 0.616 | 1.72E-04 | **4.72E-03** |
| **AIF1** | 6p21.33 | 0.616 | 1.73E-04 | **4.73E-03** |
| **FYTTD1** | 3q29 | 0.616 | 1.73E-04 | **4.73E-03** |
| **SDAD1** | 4q21.1 | 0.616 | 1.74E-04 | **4.75E-03** |
| **GFPT1** | 2p13.3 | 0.616 | 1.75E-04 | **4.76E-03** |
| **GINS1** | 20p11.21 | 0.616 | 1.75E-04 | **4.76E-03** |
| **SLC8A1** | 2p22.1 | 0.616 | 1.76E-04 | **4.79E-03** |
| **OSGIN2** | 8q21.3 | 0.616 | 1.77E-04 | **4.79E-03** |
| **DEPDC7** | 11p13 | 0.616 | 1.77E-04 | **4.80E-03** |
| **ZNF724** | 19p12 | 0.615 | 1.78E-04 | **4.80E-03** |
| **NNT** | 5p12 | 0.615 | 1.78E-04 | **4.80E-03** |
| **STX2** | 12q24.33 | 0.615 | 1.78E-04 | **4.80E-03** |
| **SPTSSA** | 14q13.1 | 0.615 | 1.79E-04 | **4.82E-03** |
| **UBE2V2** | 8q11.21 | 0.615 | 1.80E-04 | **4.83E-03** |
| **MMP9** | 20q13.12 | 0.615 | 1.80E-04 | **4.83E-03** |
| **STEAP4** | 7q21.12 | 0.615 | 1.80E-04 | **4.84E-03** |
| **TMEM192** | 4q32.3 | 0.615 | 1.82E-04 | **4.86E-03** |
| **TXNRD1** | 12q23.3 | 0.614 | 1.83E-04 | **4.89E-03** |
| **RORA** | 15q22.2 | 0.614 | 1.83E-04 | **4.89E-03** |
| **RAB31** | 18p11.22 | 0.614 | 1.84E-04 | **4.90E-03** |
| **MCTP1** | 5q15 | 0.614 | 1.84E-04 | **4.91E-03** |
| **MN1** | 22q12.1 | 0.614 | 1.86E-04 | **4.94E-03** |
| **PRKD3** | 2p22.2 | 0.614 | 1.86E-04 | **4.94E-03** |
| **SIRPG** | 20p13 | 0.614 | 1.87E-04 | **4.95E-03** |
| **PRXL2C** | 9q22.33 | 0.614 | 1.87E-04 | **4.95E-03** |
| **NR4A3** | 9q31.1 | 0.614 | 1.88E-04 | **4.96E-03** |
| **FAM92B** | 16q24.1 | 0.613 | 1.89E-04 | **4.98E-03** |
| **RAB37** | 17q25.1 | 0.613 | 1.90E-04 | **5.00E-03** |
| **RBM15** | 1p13.3 | 0.613 | 1.90E-04 | **5.00E-03** |
| **C1R** | 12p13.31 | 0.613 | 1.90E-04 | **5.00E-03** |
| **TSC22D3** | Xq22.3 | 0.613 | 1.90E-04 | **5.00E-03** |
| **SLC16A4** | 1p13.3 | 0.613 | 1.91E-04 | **5.00E-03** |
| **HEATR5A** | 14q12 | 0.613 | 1.91E-04 | **5.00E-03** |
| **JARID2** | 6p22.3 | 0.613 | 1.92E-04 | **5.00E-03** |
| **PCBP3** | 21q22.3 | 0.613 | 1.92E-04 | **5.01E-03** |
| **TLDC2** | 20q11.23 | 0.613 | 1.92E-04 | **5.01E-03** |
| **COL4A4** | 2q36.3 | 0.613 | 1.93E-04 | **5.03E-03** |
| **SELL** | 1q24.2 | 0.613 | 1.94E-04 | **5.03E-03** |
| **SRSF12** | 6q15 | 0.613 | 1.94E-04 | **5.03E-03** |
| **RO60** | 1q31.2 | 0.613 | 1.94E-04 | **5.03E-03** |
| **LGI2** | 4p15.2 | 0.612 | 1.95E-04 | **5.03E-03** |
| **SGTB** | 5q12.3 | 0.612 | 1.95E-04 | **5.03E-03** |
| **TRIM36** | 5q22.3 | 0.612 | 1.95E-04 | **5.03E-03** |
| **TMEM38B** | 9q31.2 | 0.612 | 1.96E-04 | **5.03E-03** |
| **GCLM** | 1p22.1 | 0.612 | 1.96E-04 | **5.03E-03** |
| **BNC2** | 9p22.3-p22.2 | 0.612 | 1.96E-04 | **5.03E-03** |
| **DACT3** | 19q13.32 | 0.612 | 1.96E-04 | **5.03E-03** |
| **CACYBP** | 1q25.1 | 0.612 | 1.97E-04 | **5.03E-03** |
| **SLCO2B1** | 11q13.4 | 0.612 | 1.97E-04 | **5.03E-03** |
| **SLF1** | 5q15 | 0.612 | 1.97E-04 | **5.03E-03** |
| **GPR137C** | 14q22.1 | 0.612 | 1.97E-04 | **5.03E-03** |
| **FAM217B** | 20q13.33 | 0.612 | 1.98E-04 | **5.03E-03** |
| **ARF4** | 3p14.3 | 0.612 | 1.98E-04 | **5.03E-03** |
| **CLCN4** | Xp22.2 | 0.612 | 1.98E-04 | **5.03E-03** |
| **FANCM** | 14q21.2 | 0.612 | 1.99E-04 | **5.04E-03** |
| **UCHL1** | 4p13 | 0.612 | 1.99E-04 | **5.04E-03** |
| **SEC23A** | 14q21.1 | 0.612 | 2.00E-04 | **5.05E-03** |
| **PKP2** | 12p11.21 | 0.612 | 2.00E-04 | **5.05E-03** |
| **QKI** | 6q26 | 0.611 | 2.01E-04 | **5.05E-03** |
| **TRMT6** | 20p12.3 | 0.611 | 2.01E-04 | **5.05E-03** |
| **PDS5B** | 13q13.1 | 0.611 | 2.01E-04 | **5.05E-03** |
| **SNX4** | 3q21.2 | 0.611 | 2.02E-04 | **5.06E-03** |
| **RUNDC3B** | 7q21.12 | 0.611 | 2.03E-04 | **5.07E-03** |
| **NSRP1** | 17q11.2 | 0.611 | 2.03E-04 | **5.07E-03** |
| **RSRC1** | 3q25.32 | 0.611 | 2.03E-04 | **5.08E-03** |
| **SERPINE2** | 2q36.1 | 0.611 | 2.04E-04 | **5.08E-03** |
| **RENBP** | Xq28 | 0.611 | 2.04E-04 | **5.08E-03** |
| **NFIL3** | 9q22.31 | 0.611 | 2.04E-04 | **5.08E-03** |
| **SLFN11** | 17q12 | 0.611 | 2.05E-04 | **5.09E-03** |
| **CD2** | 1p13.1 | 0.611 | 2.06E-04 | **5.12E-03** |
| **FCGR1B** | 1p11.2 | 0.611 | 2.07E-04 | **5.12E-03** |
| **OPA1** | 3q29 | 0.61 | 2.07E-04 | **5.13E-03** |
| **ADGRE3** | 19p13.12 | 0.61 | 2.08E-04 | **5.15E-03** |
| **LTBP1** | 2p22.3 | 0.61 | 2.10E-04 | **5.18E-03** |
| **ARPC5** | 1q25.3 | 0.61 | 2.11E-04 | **5.20E-03** |
| **IL13RA1** | Xq24 | 0.61 | 2.13E-04 | **5.24E-03** |
| **WAC** | 10p12.1\|10p12.1-p11.2 | 0.61 | 2.13E-04 | **5.24E-03** |
| **ABCC9** | 12p12.1 | 0.609 | 2.15E-04 | **5.27E-03** |
| **RGS18** | 1q31.2 | 0.609 | 2.15E-04 | **5.27E-03** |
| **ACTR2** | 2p14 | 0.609 | 2.16E-04 | **5.27E-03** |
| **NKIRAS1** | 3p24.2 | 0.609 | 2.16E-04 | **5.27E-03** |
| **MAD2L1** | 4q27 | 0.609 | 2.19E-04 | **5.34E-03** |
| **PRKAR2B** | 7q22.3 | 0.609 | 2.19E-04 | **5.34E-03** |
| **HERC5** | 4q22.1 | 0.609 | 2.19E-04 | **5.34E-03** |
| **GNPNAT1** | 14q22.1 | 0.608 | 2.20E-04 | **5.36E-03** |
| **TWISTNB** | 7p21.1 | 0.608 | 2.20E-04 | **5.36E-03** |
| **PRKX** | Xp22.33 | 0.608 | 2.22E-04 | **5.36E-03** |
| **RNASEH1** | 2p25.3 | 0.608 | 2.22E-04 | **5.36E-03** |
| **SPP1** | 4q22.1 | 0.608 | 2.22E-04 | **5.36E-03** |
| **AKAP12** | 6q25.1 | 0.608 | 2.22E-04 | **5.36E-03** |
| **ETV1** | 7p21.2 | 0.608 | 2.23E-04 | **5.39E-03** |
| **SYNPO2** | 4q26 | 0.608 | 2.24E-04 | **5.39E-03** |
| **C16ORF72** | 16p13.2 | 0.608 | 2.24E-04 | **5.40E-03** |
| **ENTHD1** | 22q13.1 | 0.608 | 2.25E-04 | **5.41E-03** |
| **DPT** | 1q24.2 | 0.608 | 2.25E-04 | **5.41E-03** |
| **FN1** | 2q35 | 0.607 | 2.27E-04 | **5.43E-03** |
| **ZCCHC24** | 10q22.3 | 0.607 | 2.28E-04 | **5.45E-03** |
| **CCL18** | 17q12 | 0.607 | 2.29E-04 | **5.47E-03** |
| **FMO2** | 1q24.3 | 0.607 | 2.29E-04 | **5.47E-03** |
| **SLC17A5** | 6q13 | 0.607 | 2.29E-04 | **5.47E-03** |
| **SCML1** | Xp22.13 | 0.607 | 2.30E-04 | **5.48E-03** |
| **SSR3** | 3q25.31 | 0.607 | 2.30E-04 | **5.48E-03** |
| **SOCS3** | 17q25.3 | 0.607 | 2.31E-04 | **5.48E-03** |
| **DPP4** | 2q24.2 | 0.607 | 2.31E-04 | **5.48E-03** |
| **CCNY** | 10p11.21 | 0.607 | 2.31E-04 | **5.48E-03** |
| **CCSER2** | 10q23.1 | 0.607 | 2.32E-04 | **5.49E-03** |
| **SGCD** | 5q33.2-q33.3 | 0.607 | 2.33E-04 | **5.50E-03** |
| **IFT52** | 20q13.12 | 0.606 | 2.34E-04 | **5.53E-03** |
| **C5AR2** | 19q13.32 | 0.606 | 2.34E-04 | **5.53E-03** |
| **IL17RD** | 3p14.3 | 0.606 | 2.35E-04 | **5.53E-03** |
| **PRDM1** | 6q21 | 0.606 | 2.35E-04 | **5.53E-03** |
| **SGO2** | 2q33.1 | 0.606 | 2.35E-04 | **5.53E-03** |
| **CHMP2B** | 3p11.2 | 0.606 | 2.36E-04 | **5.53E-03** |
| **FMO3** | 1q24.3 | 0.606 | 2.37E-04 | **5.53E-03** |
| **PSMG1** | 21q22.2 | 0.606 | 2.37E-04 | **5.53E-03** |
| **UHRF1BP1L** | 12q23.1 | 0.606 | 2.37E-04 | **5.53E-03** |
| **MAP7D3** | Xq26.3 | 0.606 | 2.38E-04 | **5.56E-03** |
| **ZNF460** | 19q13.43 | 0.606 | 2.40E-04 | **5.60E-03** |
| **RAD18** | 3p25.3 | 0.605 | 2.41E-04 | **5.61E-03** |
| **RIF1** | 2q23.3 | 0.605 | 2.44E-04 | **5.66E-03** |
| **MARCO** | 2q14.2 | 0.605 | 2.44E-04 | **5.67E-03** |
| **CLOCK** | 4q12 | 0.605 | 2.45E-04 | **5.67E-03** |
| **GNG4** | 1q42.3 | 0.605 | 2.45E-04 | **5.67E-03** |
| **FMO1** | 1q24.3 | 0.605 | 2.46E-04 | **5.67E-03** |
| **CEP19** | 3q29 | 0.605 | 2.46E-04 | **5.67E-03** |
| **ZNF639** | 3q26.33 | 0.605 | 2.46E-04 | **5.67E-03** |
| **KIF2A** | 5q12.1 | 0.605 | 2.46E-04 | **5.67E-03** |
| **APPL1** | 3p14.3 | 0.605 | 2.48E-04 | **5.68E-03** |
| **CUL2** | 10p11.21 | 0.605 | 2.48E-04 | **5.68E-03** |
| **SRRM1** | 1p36.11 | 0.605 | 2.48E-04 | **5.68E-03** |
| **CCDC71L** | 7q22.3 | 0.604 | 2.48E-04 | **5.69E-03** |
| **LRIG1** | 3p14.1 | 0.604 | 2.51E-04 | **5.74E-03** |
| **NIPBL** | 5p13.2 | 0.604 | 2.52E-04 | **5.75E-03** |
| **KLF11** | 2p25.1 | 0.604 | 2.53E-04 | **5.77E-03** |
| **RPN2** | 20q11.23 | 0.604 | 2.53E-04 | **5.77E-03** |
| **CD177** | 19q13.31 | 0.604 | 2.55E-04 | **5.79E-03** |
| **LRRK2** | 12q12 | 0.604 | 2.55E-04 | **5.79E-03** |
| **PRUNE1** | 1q21.3 | 0.603 | 2.56E-04 | **5.81E-03** |
| **EXOC5** | 14q22.3 | 0.603 | 2.56E-04 | **5.81E-03** |
| **PCDHGA1** | 5q31.3 | 0.603 | 2.58E-04 | **5.84E-03** |
| **FGF2** | 4q28.1 | 0.603 | 2.58E-04 | **5.84E-03** |
| **FCGR3A** | 1q23.3 | 0.603 | 2.58E-04 | **5.84E-03** |
| **KIF1B** | 1p36.22 | 0.603 | 2.59E-04 | **5.85E-03** |
| **ERMN** | 2q24.1 | 0.603 | 2.60E-04 | **5.86E-03** |
| **C4ORF46** | 4q32.1 | 0.603 | 2.60E-04 | **5.86E-03** |
| **HBP1** | 7q22.3 | 0.603 | 2.60E-04 | **5.86E-03** |
| **BMP2K** | 4q21.21 | 0.603 | 2.63E-04 | **5.90E-03** |
| **DDX18** | 2q14.1 | 0.603 | 2.63E-04 | **5.90E-03** |
| **FANCB** | Xp22.2 | 0.602 | 2.64E-04 | **5.90E-03** |
| **GOLGA5** | 14q32.12 | 0.602 | 2.64E-04 | **5.90E-03** |
| **NCOA1** | 2p23.3 | 0.602 | 2.64E-04 | **5.90E-03** |
| **METAP2** | 12q22 | 0.602 | 2.64E-04 | **5.90E-03** |
| **UHMK1** | 1q23.3 | 0.602 | 2.64E-04 | **5.90E-03** |
| **GULP1** | 2q32.1-q32.2 | 0.602 | 2.65E-04 | **5.91E-03** |
| **ARRDC5** | 19p13.3 | 0.602 | 2.66E-04 | **5.92E-03** |
| **ASGR2** | 17p13.1 | 0.602 | 2.67E-04 | **5.93E-03** |
| **TNFRSF1B** | 1p36.22 | 0.602 | 2.67E-04 | **5.93E-03** |
| **RTN1** | 14q23.1 | 0.602 | 2.68E-04 | **5.94E-03** |
| **CCDC170** | 6q25.1 | 0.602 | 2.69E-04 | **5.94E-03** |
| **SLC39A10** | 2q32.3 | 0.602 | 2.69E-04 | **5.94E-03** |
| **RAB27A** | 15q21.3 | 0.601 | 2.72E-04 | **5.99E-03** |
| **ZYG11B** | 1p32.3 | 0.601 | 2.72E-04 | **5.99E-03** |
| **RPL7L1** | 6p21.1 | 0.601 | 2.73E-04 | **6.00E-03** |
| **CPEB4** | 5q35.2 | 0.601 | 2.73E-04 | **6.00E-03** |
| **CCN2** | 6q23.2 | 0.601 | 2.73E-04 | **6.00E-03** |
| **RCAN1** | 21q22.12 | 0.601 | 2.75E-04 | **6.03E-03** |
| **SEC31A** | 4q21.22 | 0.601 | 2.76E-04 | **6.06E-03** |
| **SLC6A16** | 19q13.33 | 0.6 | 2.80E-04 | **6.13E-03** |
| **PSMA7** | 20q13.33 | 0.6 | 2.81E-04 | **6.13E-03** |
| **PCYOX1** | 2p13.3 | 0.6 | 2.81E-04 | **6.13E-03** |
| **CXORF56** | Xq24 | 0.6 | 2.82E-04 | **6.15E-03** |
| **DIRC1** | 2q32.2 | 0.6 | 2.83E-04 | **6.17E-03** |
| **RSBN1L** | 7q11.23 | 0.6 | 2.83E-04 | **6.17E-03** |
| **DCUN1D1** | 3q26.33 | 0.6 | 2.84E-04 | **6.17E-03** |
| **DRP2** | Xq22.1 | 0.6 | 2.85E-04 | **6.18E-03** |
| **DPM1** | 20q13.13 | 0.6 | 2.85E-04 | **6.19E-03** |
| **CAPN6** | Xq23 | 0.6 | 2.86E-04 | **6.20E-03** |
| **CDKN1B** | 12p13.1 | 0.6 | 2.87E-04 | **6.21E-03** |

The pink background indicates overlapping genes between UTUC and UBUC.

**Suppl. Table 2. The top 586 genes positively correlated with SFRP2 (Spearman's correlation ≥ 0.6) in UBUC.**

| **Correlated Gene** | **Cytoband** | **Spearman's Correlation** | **p-Value** | **q-Value** |
| --- | --- | --- | --- | --- |
| **FNDC1** | 6q25.3 | 0.907 | 5.90E-154 | **1.18E-149** |
| **SFRP4** | 7p14.1 | 0.895 | 8.79E-144 | **8.80E-140** |
| **AEBP1** | 7p13 | 0.893 | 2.81E-142 | **1.88E-138** |
| **POSTN** | 13q13.3 | 0.889 | 3.78E-139 | **1.89E-135** |
| **ISLR** | 15q24.1 | 0.881 | 1.03E-133 | **4.14E-130** |
| **COL3A1** | 2q32.2 | 0.88 | 3.83E-133 | **1.28E-129** |
| **FIBIN** | 11p14.2 | 0.872 | 9.40E-128 | **2.69E-124** |
| **DCN** | 12q21.33 | 0.871 | 5.82E-127 | **1.29E-123** |
| **COL1A1** | 17q21.33 | 0.871 | 6.35E-127 | **1.29E-123** |
| **SULF1** | 8q13.2-q13.3 | 0.871 | 6.43E-127 | **1.29E-123** |
| **CPXM1** | 20p13 | 0.87 | 1.01E-126 | **1.83E-123** |
| **OMD** | 9q22.31 | 0.869 | 5.98E-126 | **9.98E-123** |
| **MFAP5** | 12p13.31 | 0.869 | 1.48E-125 | **2.29E-122** |
| **SGCD** | 5q33.2-q33.3 | 0.865 | 2.19E-123 | **3.13E-120** |
| **DPT** | 1q24.2 | 0.861 | 8.04E-121 | **1.07E-117** |
| **TGFB3** | 14q24.3 | 0.861 | 8.99E-121 | **1.13E-117** |
| **CTSK** | 1q21.3 | 0.858 | 2.03E-119 | **2.39E-116** |
| **LRRC15** | 3q29 | 0.857 | 6.70E-119 | **7.46E-116** |
| **COL10A1** | 6q22.1 | 0.854 | 3.26E-117 | **3.43E-114** |
| **PRRX1** | 1q24.2 | 0.854 | 4.89E-117 | **4.90E-114** |
| **CCDC80** | 3q13.2 | 0.854 | 7.71E-117 | **7.36E-114** |
| **GAS1** | 9q21.33 | 0.853 | 1.75E-116 | **1.59E-113** |
| **COL6A3** | 2q37.3 | 0.851 | 4.20E-115 | **3.65E-112** |
| **COL1A2** | 7q21.3 | 0.85 | 4.62E-115 | **3.85E-112** |
| **SSC5D** | 19q13.42 | 0.85 | 1.54E-114 | **1.24E-111** |
| **FAP** | 2q24.2 | 0.847 | 2.08E-113 | **1.60E-110** |
| **PDLIM3** | 4q35.1 | 0.845 | 2.60E-112 | **1.93E-109** |
| **COL5A1** | 9q34.3 | 0.845 | 3.61E-112 | **2.58E-109** |
| **EMILIN1** | 2p23.3 | 0.843 | 4.64E-111 | **3.20E-108** |
| **GREM1** | 15q13.3 | 0.842 | 1.87E-110 | **1.25E-107** |
| **P4HA3** | 11q13.4 | 0.841 | 3.85E-110 | **2.48E-107** |
| **KIAA1755** | 20q11.23 | 0.834 | 1.85E-106 | **1.15E-103** |
| **CTHRC1** | 8q22.3 | 0.832 | 7.75E-106 | **4.70E-103** |
| **ASPN** | 9q22.31 | 0.831 | 5.32E-105 | **3.14E-102** |
| **FMO1** | 1q24.3 | 0.829 | 2.04E-104 | **1.17E-101** |
| **HTRA3** | 4p16.1 | 0.829 | 2.14E-104 | **1.19E-101** |
| **CDH11** | 16q21 | 0.828 | 1.15E-103 | **6.24E-101** |
| **LUM** | 12q21.33 | 0.827 | 2.47E-103 | **1.30E-100** |
| **FMOD** | 1q32.1 | 0.826 | 4.82E-103 | **2.48E-100** |
| **PLN** | 6q22.31 | 0.826 | 8.35E-103 | **4.18E-100** |
| **MXRA8** | 1p36.33 | 0.825 | 2.69E-102 | **1.31E-99** |
| **FAM180A** | 7q33 | 0.825 | 3.08E-102 | **1.47E-99** |
| **GLT8D2** | 12q23.3 | 0.824 | 4.10E-102 | **1.91E-99** |
| **ZNF469** | 16q24.2 | 0.822 | 3.60E-101 | **1.64E-98** |
| **COL6A2** | 21q22.3 | 0.821 | 1.87E-100 | **8.34E-98** |
| **BNC2** | 9p22.3-p22.2 | 0.819 | 9.31E-100 | **4.05E-97** |
| **CALD1** | 7q33 | 0.818 | 3.68E-99 | **1.57E-96** |
| **TAGLN** | 11q23.3 | 0.818 | 4.21E-99 | **1.76E-96** |
| **HEPH** | Xq12 | 0.817 | 5.59E-99 | **2.29E-96** |
| **ZFPM2** | 8q23.1 | 0.813 | 2.71E-97 | **1.09E-94** |
| **ADAM12** | 10q26.2 | 0.813 | 4.38E-97 | **1.72E-94** |
| **GPC6** | 13q31.3-q32.1 | 0.812 | 8.38E-97 | **3.23E-94** |
| **COMP** | 19p13.11 | 0.811 | 3.40E-96 | **1.29E-93** |
| **COLEC12** | 18p11.32 | 0.808 | 4.52E-95 | **1.68E-92** |
| **SYNDIG1** | 20p11.21 | 0.807 | 1.77E-94 | **6.44E-92** |
| **OLFML3** | 1p13.2 | 0.806 | 2.08E-94 | **7.44E-92** |
| **COL16A1** | 1p35.2 | 0.806 | 2.62E-94 | **9.19E-92** |
| **PLPP4** | 10q26.12 | 0.805 | 7.42E-94 | **2.56E-91** |
| **FGF7** | 15q21.2 | 0.804 | 1.69E-93 | **5.75E-91** |
| **GFPT2** | 5q35.3 | 0.804 | 2.31E-93 | **7.70E-91** |
| **ITGA11** | 15q23 | 0.803 | 3.95E-93 | **1.30E-90** |
| **COL11A1** | 1p21.1 | 0.803 | 4.17E-93 | **1.35E-90** |
| **BGN** | Xq28 | 0.803 | 4.34E-93 | **1.38E-90** |
| **MSRB3** | 12q14.3 | 0.802 | 1.11E-92 | **3.47E-90** |
| **ADAMTS2** | 5q35.3 | 0.802 | 1.73E-92 | **5.32E-90** |
| **RSPO3** | 6q22.33 | 0.8 | 4.64E-92 | **1.41E-89** |
| **COL5A2** | 2q32.2 | 0.8 | 5.79E-92 | **1.73E-89** |
| **FBN1** | 15q21.1 | 0.799 | 1.53E-91 | **4.50E-89** |
| **NTM** | 11q25 | 0.799 | 2.53E-91 | **7.33E-89** |
| **KCNE4** | 2q36.1 | 0.796 | 2.73E-90 | **7.80E-88** |
| **TNFAIP6** | 2q23.3 | 0.794 | 1.65E-89 | **4.64E-87** |
| **PDGFRB** | 5q32 | 0.793 | 3.97E-89 | **1.10E-86** |
| **OLFML1** | 11p15.4 | 0.793 | 4.00E-89 | **1.10E-86** |
| **PLA2G5** | 1p36.13 | 0.79 | 3.14E-88 | **8.50E-86** |
| **VCAN** | 5q14.2-q14.3 | 0.789 | 1.23E-87 | **3.28E-85** |
| **CAMK2A** | 5q32 | 0.788 | 2.69E-87 | **7.09E-85** |
| **SPARC** | 5q33.1 | 0.788 | 3.52E-87 | **9.15E-85** |
| **CSDC2** | 22q13.2 | 0.787 | 7.47E-87 | **1.92E-84** |
| **CHRDL2** | 11q13.4 | 0.787 | 7.56E-87 | **1.92E-84** |
| **LAMP5** | 20p12.2 | 0.785 | 3.37E-86 | **8.43E-84** |
| **TIMP2** | 17q25.3 | 0.784 | 5.21E-86 | **1.29E-83** |
| **PCOLCE** | 7q22.1 | 0.784 | 8.86E-86 | **2.16E-83** |
| **GXYLT2** | 3p13 | 0.783 | 1.24E-85 | **2.99E-83** |
| **TENM3** | 4q34.3-q35.1 | 0.782 | 2.67E-85 | **6.37E-83** |
| **COL8A2** | 1p34.3 | 0.782 | 3.78E-85 | **8.90E-83** |
| **SPON1** | 11p15.2 | 0.782 | 4.40E-85 | **1.02E-82** |
| **RARRES2** | 7q36.1 | 0.779 | 2.73E-84 | **6.28E-82** |
| **THBS2** | 6q27 | 0.778 | 6.89E-84 | **1.57E-81** |
| **TWIST2** | 2q37.3 | 0.778 | 9.07E-84 | **2.04E-81** |
| **SCARF2** | 22q11.21 | 0.778 | 1.14E-83 | **2.53E-81** |
| **ACTA2** | 10q23.31 | 0.777 | 1.67E-83 | **3.67E-81** |
| **PALLD** | 4q32.3 | 0.777 | 2.32E-83 | **5.06E-81** |
| **ADAMTS16** | 5p15.32 | 0.776 | 3.00E-83 | **6.46E-81** |
| **OLFML2B** | 1q23.3 | 0.776 | 4.28E-83 | **9.12E-81** |
| **SPON2** | 4p16.3 | 0.775 | 1.44E-82 | **3.03E-80** |
| **CPXM2** | 10q26.13 | 0.774 | 1.52E-82 | **3.17E-80** |
| **DACT1** | 14q23.1 | 0.774 | 2.18E-82 | **4.51E-80** |
| **ST6GALNAC5** | 1p31.1 | 0.774 | 2.74E-82 | **5.60E-80** |
| **CILP** | 15q22.31 | 0.773 | 5.87E-82 | **1.19E-79** |
| **GALNT17** | 7q11.22 | 0.773 | 5.98E-82 | **1.20E-79** |
| **COL6A1** | 21q22.3 | 0.768 | 1.56E-80 | **3.08E-78** |
| **LMCD1** | 3p25.3 | 0.768 | 1.61E-80 | **3.16E-78** |
| **HSPB2** | 11q23.1 | 0.768 | 2.55E-80 | **4.96E-78** |
| **ARSI** | 5q32 | 0.768 | 3.03E-80 | **5.84E-78** |
| **ANXA6** | 5q33.1 | 0.767 | 4.65E-80 | **8.86E-78** |
| **PODNL1** | 19p13.12 | 0.767 | 6.50E-80 | **1.23E-77** |
| **ZNF521** | 18q11.2 | 0.765 | 2.08E-79 | **3.89E-77** |
| **CCN4** | 8q24.22 | 0.764 | 4.28E-79 | **7.94E-77** |
| **GAS7** | 17p13.1 | 0.763 | 1.04E-78 | **1.90E-76** |
| **PDZRN3** | 3p13 | 0.763 | 1.26E-78 | **2.29E-76** |
| **C1S** | 12p13.31 | 0.762 | 1.56E-78 | **2.82E-76** |
| **DIRC1** | 2q32.2 | 0.761 | 3.40E-78 | **6.09E-76** |
| **CNN1** | 19p13.2 | 0.761 | 4.82E-78 | **8.53E-76** |
| **NEGR1** | 1p31.1 | 0.76 | 9.20E-78 | **1.62E-75** |
| **DACT3** | 19q13.32 | 0.758 | 2.65E-77 | **4.62E-75** |
| **MYL9** | 20q11.23 | 0.758 | 4.52E-77 | **7.80E-75** |
| **APCDD1L** | 20q13.32 | 0.758 | 4.75E-77 | **8.13E-75** |
| **HIC1** | 17p13.3 | 0.757 | 8.11E-77 | **1.38E-74** |
| **GLI2** | 2q14.2 | 0.757 | 1.01E-76 | **1.71E-74** |
| **FAM20C** | 7p22.3 | 0.756 | 1.08E-76 | **1.81E-74** |
| **OGN** | 9q22.31 | 0.756 | 1.67E-76 | **2.76E-74** |
| **PLPP7** | 9q34.13 | 0.755 | 2.74E-76 | **4.50E-74** |
| **THY1** | 11q23.3 | 0.754 | 8.33E-76 | **1.36E-73** |
| **KLF17** | 1p34.1 | 0.754 | 8.68E-76 | **1.40E-73** |
| **MEOX2** | 7p21.2 | 0.753 | 1.05E-75 | **1.68E-73** |
| **CALHM5** | 6q22.1 | 0.753 | 1.24E-75 | **1.96E-73** |
| **SHISAL1** | 22q13.31 | 0.753 | 1.26E-75 | **1.98E-73** |
| **MYLK** | 3q21.1 | 0.752 | 2.11E-75 | **3.29E-73** |
| **NNMT** | 11q23.2 | 0.752 | 2.12E-75 | **3.29E-73** |
| **SERPINF1** | 17p13.3 | 0.752 | 2.79E-75 | **4.30E-73** |
| **TPM2** | 9p13.3 | 0.75 | 1.04E-74 | **1.59E-72** |
| **FN1** | 2q35 | 0.75 | 1.17E-74 | **1.77E-72** |
| **ECM2** | 9q22.31 | 0.75 | 1.47E-74 | **2.21E-72** |
| **MMP2** | 16q12.2 | 0.749 | 1.93E-74 | **2.88E-72** |
| **HSPB7** | 1p36.13 | 0.749 | 2.14E-74 | **3.18E-72** |
| **PXDN** | 2p25.3 | 0.748 | 4.43E-74 | **6.53E-72** |
| **NEXN** | 1p31.1 | 0.747 | 8.78E-74 | **1.28E-71** |
| **FMO2** | 1q24.3 | 0.747 | 9.85E-74 | **1.43E-71** |
| **ROR2** | 9q22.31 | 0.747 | 1.06E-73 | **1.52E-71** |
| **NTNG2** | 9q34.13 | 0.746 | 1.95E-73 | **2.79E-71** |
| **TNFSF4** | 1q25.1 | 0.745 | 4.26E-73 | **6.04E-71** |
| **GUCA1A** | 6p21.1 | 0.744 | 4.74E-73 | **6.68E-71** |
| **MSC** | 8q13.3 | 0.744 | 7.59E-73 | **1.06E-70** |
| **ANGPTL2** | 9q33.3 | 0.744 | 8.30E-73 | **1.15E-70** |
| **KCNJ8** | 12p12.1 | 0.743 | 1.11E-72 | **1.53E-70** |
| **MIR100HG** | 11q24.1 | 0.742 | 2.53E-72 | **3.47E-70** |
| **FAM20A** | 17q24.2 | 0.742 | 3.17E-72 | **4.32E-70** |
| **GPR1** | 2q33.3 | 0.741 | 3.65E-72 | **4.95E-70** |
| **MRGPRF** | 11q13.3 | 0.741 | 4.17E-72 | **5.60E-70** |
| **ADAMTS12** | 5p13.3-p13.2 | 0.74 | 1.12E-71 | **1.50E-69** |
| **PLXNA4** | 7q32.3 | 0.74 | 1.24E-71 | **1.64E-69** |
| **FLNC** | 7q32.1 | 0.739 | 1.38E-71 | **1.82E-69** |
| **ALPK2** | 18q21.31-q21.32 | 0.739 | 2.13E-71 | **2.79E-69** |
| **RGS4** | 1q23.3 | 0.737 | 5.19E-71 | **6.75E-69** |
| **WNT2** | 7q31.2 | 0.737 | 9.30E-71 | **1.20E-68** |
| **RFLNA** | 12q24.31 | 0.736 | 1.46E-70 | **1.87E-68** |
| **ALDH1L2** | 12q23.3 | 0.736 | 1.59E-70 | **2.02E-68** |
| **RAB3IL1** | 11q12.2-q12.3 | 0.736 | 1.70E-70 | **2.15E-68** |
| **FBLN2** | 3p25.1 | 0.735 | 1.89E-70 | **2.38E-68** |
| **PTGER3** | 1p31.1 | 0.735 | 1.95E-70 | **2.44E-68** |
| **COPZ2** | 17q21.32 | 0.735 | 2.11E-70 | **2.62E-68** |
| **RCN3** | 19q13.33 | 0.735 | 3.19E-70 | **3.94E-68** |
| **CRISPLD2** | 16q24.1 | 0.734 | 5.26E-70 | **6.46E-68** |
| **C1R** | 12p13.31 | 0.734 | 5.93E-70 | **7.24E-68** |
| **CCIN** | 9p13.3 | 0.733 | 7.26E-70 | **8.81E-68** |
| **CYS1** | 2p25.1 | 0.733 | 9.32E-70 | **1.12E-67** |
| **ANTXR1** | 2p13.3 | 0.733 | 1.13E-69 | **1.36E-67** |
| **GYPC** | 2q14.3 | 0.732 | 1.48E-69 | **1.76E-67** |
| **TWIST1** | 7p21.1 | 0.732 | 1.82E-69 | **2.15E-67** |
| **LOX** | 5q23.1 | 0.732 | 1.91E-69 | **2.25E-67** |
| **EDNRA** | 4q31.22-q31.23 | 0.732 | 2.34E-69 | **2.74E-67** |
| **MEDAG** | 13q12.3 | 0.731 | 3.62E-69 | **4.21E-67** |
| **SUGCT** | 7p14.1 | 0.731 | 3.64E-69 | **4.21E-67** |
| **EPYC** | 12q21.33 | 0.731 | 4.00E-69 | **4.61E-67** |
| **GLIS1** | 1p32.3 | 0.73 | 5.64E-69 | **6.46E-67** |
| **MN1** | 22q12.1 | 0.73 | 5.71E-69 | **6.50E-67** |
| **PODN** | 1p32.3 | 0.729 | 1.39E-68 | **1.57E-66** |
| **LRRC32** | 11q13.5 | 0.728 | 1.75E-68 | **1.97E-66** |
| **MMP11** | 22q11.23 | 0.728 | 2.17E-68 | **2.43E-66** |
| **CSMD2** | 1p35.1 | 0.727 | 4.23E-68 | **4.71E-66** |
| **ADAMTS14** | 10q22.1 | 0.726 | 5.69E-68 | **6.29E-66** |
| **RTL3** | Xq21.1 | 0.725 | 1.24E-67 | **1.37E-65** |
| **NID2** | 14q22.1 | 0.725 | 1.58E-67 | **1.73E-65** |
| **C1QTNF1** | 17q25.3 | 0.725 | 1.83E-67 | **2.00E-65** |
| **CLEC11A** | 19q13.33 | 0.724 | 3.10E-67 | **3.35E-65** |
| **DDR2** | 1q23.3 | 0.723 | 5.55E-67 | **5.98E-65** |
| **CCL21** | 9p13.3 | 0.723 | 5.70E-67 | **6.11E-65** |
| **NRP2** | 2q33.3 | 0.721 | 1.69E-66 | **1.80E-64** |
| **CCN2** | 6q23.2 | 0.721 | 1.79E-66 | **1.89E-64** |
| **BOC** | 3q13.2 | 0.72 | 2.97E-66 | **3.13E-64** |
| **CD248** | 11q13.2 | 0.72 | 3.94E-66 | **4.13E-64** |
| **CAVIN1** | 17q21.2 | 0.72 | 4.05E-66 | **4.23E-64** |
| **ITGBL1** | 13q33.1 | 0.719 | 4.59E-66 | **4.76E-64** |
| **ACTG2** | 2p13.1 | 0.718 | 9.25E-66 | **9.55E-64** |
| **TPM1** | 15q22.2 | 0.718 | 9.63E-66 | **9.89E-64** |
| **PRRX2** | 9q34.11 | 0.718 | 9.80E-66 | **1.00E-63** |
| **MRC2** | 17q23.2 | 0.718 | 1.01E-65 | **1.03E-63** |
| **ABCC9** | 12p12.1 | 0.718 | 1.19E-65 | **1.21E-63** |
| **LMOD1** | 1q32.1 | 0.718 | 1.24E-65 | **1.25E-63** |
| **CXCL12** | 10q11.21 | 0.718 | 1.30E-65 | **1.30E-63** |
| **MAP1A** | 15q15.3 | 0.717 | 2.10E-65 | **2.10E-63** |
| **P2RX1** | 17p13.2 | 0.716 | 2.59E-65 | **2.57E-63** |
| **ADGRA2** | 8p11.23 | 0.716 | 3.77E-65 | **3.72E-63** |
| **ACTC1** | 15q14 | 0.716 | 3.84E-65 | **3.77E-63** |
| **IFFO1** | 12p13.31 | 0.715 | 4.61E-65 | **4.50E-63** |
| **HS3ST3A1** | 17p12 | 0.714 | 8.31E-65 | **8.08E-63** |
| **IGF1** | 12q23.2 | 0.714 | 1.04E-64 | **1.01E-62** |
| **VIM** | 10p13 | 0.713 | 1.84E-64 | **1.77E-62** |
| **TENM3-AS1** | 4q34.3 | 0.713 | 1.97E-64 | **1.89E-62** |
| **PRELP** | 1q32.1 | 0.712 | 2.72E-64 | **2.59E-62** |
| **LEFTY2** | 1q42.12 | 0.711 | 5.73E-64 | **5.44E-62** |
| **DES** | 2q35 | 0.711 | 6.58E-64 | **6.22E-62** |
| **FBXL7** | 5p15.1 | 0.711 | 8.19E-64 | **7.70E-62** |
| **PTPRD** | 9p24.1-p23 | 0.71 | 1.24E-63 | **1.16E-61** |
| **ADAMTS10** | 19p13.2 | 0.71 | 1.26E-63 | **1.17E-61** |
| **CYBRD1** | 2q31.1 | 0.709 | 1.93E-63 | **1.79E-61** |
| **CCL11** | 17q12 | 0.709 | 2.42E-63 | **2.24E-61** |
| **SCRG1** | 4q34.1 | 0.708 | 4.38E-63 | **4.03E-61** |
| **STK32B** | 4p16.2 | 0.707 | 5.22E-63 | **4.77E-61** |
| **FILIP1L** | 3q12.1 | 0.707 | 6.35E-63 | **5.78E-61** |
| **KCNMB1** | 5q35.1 | 0.707 | 6.83E-63 | **6.19E-61** |
| **GPR68** | 14q32.11 | 0.707 | 7.46E-63 | **6.73E-61** |
| **HSPA12B** | 20p13 | 0.706 | 8.79E-63 | **7.89E-61** |
| **PTGIS** | 20q13.13 | 0.705 | 1.95E-62 | **1.74E-60** |
| **LY96** | 8q21.11 | 0.705 | 2.62E-62 | **2.33E-60** |
| **ATP10A** | 15q12 | 0.704 | 2.90E-62 | **2.57E-60** |
| **FXYD6** | 11q23.3 | 0.704 | 3.64E-62 | **3.22E-60** |
| **TCEAL7** | Xq22.2 | 0.704 | 4.43E-62 | **3.89E-60** |
| **HTRA1** | 10q26.13 | 0.704 | 4.63E-62 | **4.05E-60** |
| **ZEB2** | 2q22.3 | 0.703 | 5.37E-62 | **4.68E-60** |
| **CRYAB** | 11q23.1 | 0.702 | 1.06E-61 | **9.19E-60** |
| **GGT5** | 22q11.23 | 0.701 | 1.81E-61 | **1.57E-59** |
| **COL8A1** | 3q12.1 | 0.701 | 2.10E-61 | **1.80E-59** |
| **COL15A1** | 9q22.33 | 0.7 | 2.69E-61 | **2.31E-59** |
| **GEM** | 8q22.1 | 0.7 | 3.92E-61 | **3.34E-59** |
| **RUBCNL** | 13q14.13 | 0.699 | 4.54E-61 | **3.85E-59** |
| **HTR2A** | 13q14.2 | 0.699 | 5.38E-61 | **4.54E-59** |
| **CADM3** | 1q23.2 | 0.699 | 6.38E-61 | **5.37E-59** |
| **HAND2** | 4q34.1 | 0.699 | 7.47E-61 | **6.26E-59** |
| **TGFB1I1** | 16p11.2 | 0.697 | 1.75E-60 | **1.46E-58** |
| **EFEMP1** | 2p16.1 | 0.694 | 8.37E-60 | **6.95E-58** |
| **EVI2A** | 17q11.2 | 0.693 | 1.33E-59 | **1.10E-57** |
| **ADGRD1** | 12q24.33 | 0.693 | 1.52E-59 | **1.26E-57** |
| **C4A** | 6p21.33 | 0.693 | 1.65E-59 | **1.36E-57** |
| **MMP23B** | 1p36.33 | 0.693 | 1.82E-59 | **1.49E-57** |
| **KANK4** | 1p31.3 | 0.692 | 2.23E-59 | **1.81E-57** |
| **DPYSL3** | 5q32 | 0.692 | 2.27E-59 | **1.84E-57** |
| **VSTM4** | 10q11.23 | 0.692 | 2.44E-59 | **1.97E-57** |
| **GAS6** | 13q34 | 0.691 | 5.13E-59 | **4.12E-57** |
| **MFRP** | 11q23.3 | 0.69 | 6.70E-59 | **5.36E-57** |
| **TMEM119** | 12q23.3 | 0.69 | 9.30E-59 | **7.42E-57** |
| **ITGA5** | 12q13.13 | 0.689 | 1.51E-58 | **1.20E-56** |
| **MRVI1** | 11p15.4 | 0.688 | 2.25E-58 | **1.78E-56** |
| **CHRD** | 3q27.1 | 0.688 | 2.33E-58 | **1.84E-56** |
| **CAVIN3** | 11p15.4 | 0.688 | 2.55E-58 | **2.00E-56** |
| **HSD17B6** | 12q13.3 | 0.687 | 4.65E-58 | **3.64E-56** |
| **SGIP1** | 1p31.3 | 0.687 | 4.98E-58 | **3.88E-56** |
| **SMOC2** | 6q27 | 0.687 | 5.13E-58 | **3.98E-56** |
| **C5ORF46** | 5q32 | 0.685 | 1.00E-57 | **7.77E-56** |
| **RAB23** | 6p12.1-p11.2 | 0.685 | 1.09E-57 | **8.42E-56** |
| **DOK5** | 20q13.2 | 0.685 | 1.14E-57 | **8.75E-56** |
| **ZCCHC24** | 10q22.3 | 0.684 | 1.59E-57 | **1.21E-55** |
| **FGFR1** | 8p11.23 | 0.684 | 1.91E-57 | **1.46E-55** |
| **NAP1L3** | Xq21.32 | 0.683 | 3.23E-57 | **2.45E-55** |
| **WIPF1** | 2q31.1 | 0.683 | 3.67E-57 | **2.78E-55** |
| **MSR1** | 8p22 | 0.683 | 3.89E-57 | **2.93E-55** |
| **SCN1B** | 19q13.11 | 0.682 | 4.58E-57 | **3.44E-55** |
| **MOXD1** | 6q23.2 | 0.682 | 4.74E-57 | **3.54E-55** |
| **HSPB6** | 19q13.12 | 0.68 | 1.29E-56 | **9.57E-55** |
| **EMP3** | 19q13.33 | 0.679 | 2.10E-56 | **1.56E-54** |
| **RPLP0P2** | 11q12.2 | 0.679 | 2.94E-56 | **2.17E-54** |
| **ANGPTL1** | 1q25.2 | 0.678 | 3.76E-56 | **2.77E-54** |
| **COL5A3** | 19p13.2 | 0.678 | 4.57E-56 | **3.35E-54** |
| **PTGDR** | 14q22.1 | 0.677 | 5.86E-56 | **4.29E-54** |
| **CLIP3** | 19q13.12 | 0.677 | 6.34E-56 | **4.61E-54** |
| **CNRIP1** | 2p14 | 0.676 | 9.49E-56 | **6.88E-54** |
| **THBS1** | 15q14 | 0.676 | 1.18E-55 | **8.53E-54** |
| **OSCAR** | 19q13.42 | 0.676 | 1.29E-55 | **9.29E-54** |
| **FBLN5** | 14q32.12 | 0.676 | 1.42E-55 | **1.02E-53** |
| **TAFA5** | 22q13.32 | 0.676 | 1.45E-55 | **1.04E-53** |
| **KCNJ12** | 17p11.2 | 0.676 | 1.45E-55 | **1.04E-53** |
| **CHI3L1** | 1q32.1 | 0.675 | 1.82E-55 | **1.29E-53** |
| **JAM3** | 11q25 | 0.675 | 2.04E-55 | **1.45E-53** |
| **HAND2-AS1** | 4q34.1 | 0.674 | 2.52E-55 | **1.78E-53** |
| **CD163** | 12p13.31 | 0.674 | 3.71E-55 | **2.61E-53** |
| **AXL** | 19q13.2 | 0.673 | 4.84E-55 | **3.39E-53** |
| **SOD3** | 4p15.2 | 0.673 | 5.76E-55 | **4.02E-53** |
| **PDZRN4** | 12q12 | 0.673 | 6.07E-55 | **4.22E-53** |
| **C5AR1** | 19q13.32 | 0.672 | 6.80E-55 | **4.72E-53** |
| **PDPN** | 1p36.21 | 0.672 | 7.41E-55 | **5.11E-53** |
| **PTGS1** | 9q33.2 | 0.672 | 9.99E-55 | **6.88E-53** |
| **PNMA8B** | 19q13.32 | 0.671 | 1.28E-54 | **8.77E-53** |
| **GLIS2** | 16p13.3 | 0.67 | 2.47E-54 | **1.69E-52** |
| **PRUNE2** | 9q21.2 | 0.669 | 3.78E-54 | **2.57E-52** |
| **SPHK1** | 17q25.1 | 0.669 | 4.69E-54 | **3.18E-52** |
| **PRICKLE1** | 12q12 | 0.668 | 5.78E-54 | **3.91E-52** |
| **FLNA** | Xq28 | 0.667 | 9.99E-54 | **6.73E-52** |
| **RAMP1** | 2q37.3 | 0.667 | 1.20E-53 | **8.09E-52** |
| **GSTM5** | 1p13.3 | 0.667 | 1.21E-53 | **8.11E-52** |
| **RNASE6** | 14q11.2 | 0.666 | 1.56E-53 | **1.04E-51** |
| **FCGR1CP** | 1q21.1 | 0.666 | 1.75E-53 | **1.16E-51** |
| **MS4A4A** | 11q12.2 | 0.666 | 1.88E-53 | **1.25E-51** |
| **FPR1** | 19q13.41 | 0.666 | 2.02E-53 | **1.33E-51** |
| **CDX1** | 5q32 | 0.665 | 2.25E-53 | **1.48E-51** |
| **MFAP4** | 17p11.2 | 0.665 | 2.40E-53 | **1.58E-51** |
| **CCDC8** | 19q13.32 | 0.665 | 2.55E-53 | **1.67E-51** |
| **VSIG4** | Xq12 | 0.665 | 2.60E-53 | **1.70E-51** |
| **FCGR2B** | 1q23.3 | 0.665 | 3.24E-53 | **2.11E-51** |
| **MFGE8** | 15q26.1 | 0.665 | 3.25E-53 | **2.11E-51** |
| **LHFPL6** | 13q13.3-q14.11 | 0.664 | 3.47E-53 | **2.24E-51** |
| **JPH2** | 20q13.12 | 0.664 | 3.74E-53 | **2.41E-51** |
| **LILRB3** | 19q13.42 | 0.664 | 3.86E-53 | **2.48E-51** |
| **PTGFR** | 1p31.1 | 0.664 | 4.16E-53 | **2.66E-51** |
| **XPNPEP2** | Xq26.1 | 0.664 | 4.27E-53 | **2.72E-51** |
| **FCGR2A** | 1q23.3 | 0.664 | 4.39E-53 | **2.79E-51** |
| **GPX8** | 5q11.2 | 0.664 | 5.53E-53 | **3.50E-51** |
| **IGSF21** | 1p36.13 | 0.663 | 6.14E-53 | **3.88E-51** |
| **TNN** | 1q25.1 | 0.663 | 6.89E-53 | **4.34E-51** |
| **RAB31** | 18p11.22 | 0.663 | 7.18E-53 | **4.51E-51** |
| **FCGR1A** | 1q21.2 | 0.663 | 7.22E-53 | **4.52E-51** |
| **SRPX** | Xp11.4 | 0.662 | 9.14E-53 | **5.70E-51** |
| **AOX1** | 2q33.1 | 0.662 | 1.32E-52 | **8.19E-51** |
| **NOX4** | 11q14.3 | 0.662 | 1.35E-52 | **8.39E-51** |
| **CDO1** | 5q22.3 | 0.662 | 1.42E-52 | **8.80E-51** |
| **KCNMA1** | 10q22.3 | 0.662 | 1.45E-52 | **8.91E-51** |
| **ADAM19** | 5q33.3 | 0.661 | 1.88E-52 | **1.16E-50** |
| **LIMS2** | 2q14.3 | 0.66 | 3.01E-52 | **1.84E-50** |
| **LRRC25** | 19p13.11 | 0.66 | 3.04E-52 | **1.86E-50** |
| **TNS1** | 2q35 | 0.66 | 3.07E-52 | **1.87E-50** |
| **PILRA** | 7q22.1 | 0.66 | 3.28E-52 | **1.99E-50** |
| **SCN2B** | 11q23.3 | 0.66 | 3.37E-52 | **2.04E-50** |
| **VENTX** | 10q26.3 | 0.659 | 4.13E-52 | **2.49E-50** |
| **TNFAIP8L3** | 15q21.2 | 0.659 | 4.68E-52 | **2.81E-50** |
| **SLCO2B1** | 11q13.4 | 0.659 | 4.75E-52 | **2.85E-50** |
| **TMEM176B** | 7q36.1 | 0.659 | 4.91E-52 | **2.93E-50** |
| **TNC** | 9q33.1 | 0.659 | 5.05E-52 | **3.01E-50** |
| **TM6SF2** | 19p13.11 | 0.658 | 7.02E-52 | **4.17E-50** |
| **MRO** | 18q21.2 | 0.658 | 8.53E-52 | **5.05E-50** |
| **SLAMF8** | 1q23.2 | 0.658 | 9.42E-52 | **5.57E-50** |
| **INHBA** | 7p14.1 | 0.657 | 1.01E-51 | **5.92E-50** |
| **FSTL1** | 3q13.33 | 0.657 | 1.06E-51 | **6.24E-50** |
| **TMEM176A** | 7q36.1 | 0.657 | 1.22E-51 | **7.15E-50** |
| **CHSY3** | 5q23.3 | 0.657 | 1.40E-51 | **8.19E-50** |
| **HGF** | 7q21.11 | 0.656 | 1.63E-51 | **9.52E-50** |
| **GALNT15** | 3p25.1 | 0.656 | 1.78E-51 | **1.04E-49** |
| **DNAJB5** | 9p13.3 | 0.656 | 1.91E-51 | **1.10E-49** |
| **CD14** | 5q31.3 | 0.656 | 1.92E-51 | **1.11E-49** |
| **ADAMTSL1** | 9p22.2-p22.1 | 0.655 | 2.68E-51 | **1.54E-49** |
| **PLEKHO2** | 15q22.31 | 0.655 | 2.76E-51 | **1.58E-49** |
| **RUNX1T1** | 8q21.3 | 0.655 | 3.22E-51 | **1.84E-49** |
| **GLIPR2** | 9p13.3 | 0.654 | 4.45E-51 | **2.54E-49** |
| **SIGLEC7** | 19q13.41 | 0.654 | 4.51E-51 | **2.56E-49** |
| **THEMIS2** | 1p35.3 | 0.654 | 4.67E-51 | **2.65E-49** |
| **ADRB3** | 8p11.23 | 0.654 | 4.87E-51 | **2.75E-49** |
| **XIRP1** | 3p22.2 | 0.653 | 6.34E-51 | **3.57E-49** |
| **C3AR1** | 12p13.31 | 0.653 | 7.90E-51 | **4.44E-49** |
| **TPSAB1** | 16p13.3 | 0.653 | 7.98E-51 | **4.48E-49** |
| **EGR2** | 10q21.3 | 0.653 | 8.62E-51 | **4.82E-49** |
| **FCGR1B** | 1p11.2 | 0.652 | 1.05E-50 | **5.85E-49** |
| **ITGAX** | 16p11.2 | 0.652 | 1.38E-50 | **7.66E-49** |
| **SFRP1** | 8p11.21 | 0.652 | 1.38E-50 | **7.66E-49** |
| **HPSE2** | 10q24.2 | 0.652 | 1.43E-50 | **7.93E-49** |
| **KERA** | 12q21.33 | 0.651 | 2.22E-50 | **1.22E-48** |
| **COL18A1** | 21q22.3 | 0.65 | 3.16E-50 | **1.74E-48** |
| **RASL12** | 15q22.31 | 0.649 | 4.32E-50 | **2.37E-48** |
| **BTK** | Xq22.1 | 0.649 | 4.48E-50 | **2.45E-48** |
| **PDLIM2** | 8p21.3 | 0.649 | 5.11E-50 | **2.79E-48** |
| **RECK** | 9p13.3 | 0.649 | 5.22E-50 | **2.84E-48** |
| **NCAM2** | 21q21.1 | 0.649 | 5.34E-50 | **2.90E-48** |
| **TYROBP** | 19q13.12 | 0.649 | 5.37E-50 | **2.91E-48** |
| **CARMN** | 5q32 | 0.649 | 5.48E-50 | **2.96E-48** |
| **SLIT2** | 4p15.31 | 0.648 | 6.76E-50 | **3.64E-48** |
| **CERCAM** | 9q34.11 | 0.648 | 6.78E-50 | **3.64E-48** |
| **FERMT2** | 14q22.1 | 0.648 | 7.28E-50 | **3.90E-48** |
| **CSF1R** | 5q32 | 0.648 | 8.25E-50 | **4.41E-48** |
| **SULF2** | 20q13.12 | 0.648 | 8.36E-50 | **4.45E-48** |
| **SERPING1** | 11q12.1 | 0.648 | 8.65E-50 | **4.59E-48** |
| **LGI2** | 4p15.2 | 0.648 | 8.73E-50 | **4.63E-48** |
| **TMEM240** | 1p36.33 | 0.648 | 8.78E-50 | **4.64E-48** |
| **PRR16** | 5q23.1 | 0.647 | 1.01E-49 | **5.35E-48** |
| **DIPK1C** | 18q22.3 | 0.647 | 1.12E-49 | **5.90E-48** |
| **RGS2** | 1q31.2 | 0.647 | 1.19E-49 | **6.24E-48** |
| **MS4A6A** | 11q12.2 | 0.647 | 1.30E-49 | **6.79E-48** |
| **FCGR3A** | 1q23.3 | 0.647 | 1.39E-49 | **7.26E-48** |
| **LY86** | 6p25.1 | 0.647 | 1.46E-49 | **7.60E-48** |
| **C1ORF162** | 1p13.2 | 0.646 | 1.77E-49 | **9.19E-48** |
| **COL12A1** | 6q13-q14.1 | 0.646 | 2.31E-49 | **1.20E-47** |
| **CPEB1** | 15q25.2 | 0.644 | 3.85E-49 | **1.99E-47** |
| **RSPO2** | 8q23.1 | 0.644 | 5.07E-49 | **2.61E-47** |
| **RIMBP2** | 12q24.33 | 0.642 | 1.16E-48 | **5.95E-47** |
| **KLHL4** | Xq21.31 | 0.642 | 1.23E-48 | **6.28E-47** |
| **PDGFRL** | 8p22 | 0.642 | 1.42E-48 | **7.24E-47** |
| **SH3RF3** | 2q13 | 0.641 | 1.86E-48 | **9.50E-47** |
| **NAV3** | 12q21.2 | 0.641 | 1.89E-48 | **9.63E-47** |
| **COL14A1** | 8q24.12 | 0.641 | 2.06E-48 | **1.04E-46** |
| **LAMA4** | 6q21 | 0.641 | 2.14E-48 | **1.08E-46** |
| **NBL1** | 1p36.13 | 0.641 | 2.17E-48 | **1.10E-46** |
| **COX7A1** | 19q13.12 | 0.64 | 2.33E-48 | **1.17E-46** |
| **GLIPR1** | 12q21.2 | 0.64 | 2.38E-48 | **1.20E-46** |
| **CPA3** | 3q24 | 0.64 | 2.48E-48 | **1.24E-46** |
| **PDE1A** | 2q32.1 | 0.64 | 2.69E-48 | **1.34E-46** |
| **CCN1** | 1p22.3 | 0.64 | 2.84E-48 | **1.42E-46** |
| **SELENOM** | 22q12.2 | 0.64 | 2.89E-48 | **1.43E-46** |
| **RNASE2** | 14q11.2 | 0.64 | 3.42E-48 | **1.69E-46** |
| **ZEB1** | 10p11.22 | 0.639 | 3.50E-48 | **1.73E-46** |
| **TNFRSF8** | 1p36.22 | 0.639 | 3.53E-48 | **1.74E-46** |
| **CD300LB** | 17q25.1 | 0.639 | 3.96E-48 | **1.95E-46** |
| **TGFBI** | 5q31.1 | 0.639 | 4.02E-48 | **1.97E-46** |
| **PTPRN** | 2q35 | 0.639 | 4.11E-48 | **2.01E-46** |
| **LILRB4** | 19q13.42 | 0.639 | 4.35E-48 | **2.12E-46** |
| **MMP9** | 20q13.12 | 0.639 | 4.41E-48 | **2.15E-46** |
| **PKDCC** | 2p21 | 0.639 | 5.09E-48 | **2.48E-46** |
| **RASGRP4** | 19q13.2 | 0.639 | 5.16E-48 | **2.50E-46** |
| **ADAMTS4** | 1q23.3 | 0.638 | 5.52E-48 | **2.67E-46** |
| **SPI1** | 11p11.2 | 0.638 | 5.63E-48 | **2.72E-46** |
| **CCL26** | 7q11.23 | 0.638 | 6.01E-48 | **2.89E-46** |
| **ADAMTS6** | 5q12.3 | 0.638 | 6.05E-48 | **2.90E-46** |
| **DIXDC1** | 11q23.1 | 0.638 | 7.57E-48 | **3.63E-46** |
| **UBE2QL1** | 5p15.31 | 0.638 | 7.75E-48 | **3.70E-46** |
| **SLC11A1** | 2q35 | 0.638 | 7.94E-48 | **3.79E-46** |
| **PRND** | 20p13 | 0.637 | 1.05E-47 | **4.99E-46** |
| **HSD11B1** | 1q32.2 | 0.637 | 1.07E-47 | **5.08E-46** |
| **SYT11** | 1q22 | 0.637 | 1.24E-47 | **5.87E-46** |
| **ADORA1** | 1q32.1 | 0.637 | 1.26E-47 | **5.95E-46** |
| **ASB5** | 4q34.2 | 0.636 | 1.32E-47 | **6.22E-46** |
| **CCL19** | 9p13.3 | 0.636 | 1.37E-47 | **6.43E-46** |
| **ACTN1** | 14q24.1\|14q22-q24 | 0.636 | 1.47E-47 | **6.91E-46** |
| **IL10** | 1q32.1 | 0.636 | 1.82E-47 | **8.50E-46** |
| **PTGIR** | 19q13.32 | 0.635 | 2.35E-47 | **1.10E-45** |
| **DTX1** | 12q24.13 | 0.635 | 2.64E-47 | **1.23E-45** |
| **PSD** | 10q24.32 | 0.634 | 3.16E-47 | **1.47E-45** |
| **FGF1** | 5q31.3 | 0.634 | 3.22E-47 | **1.49E-45** |
| **ITGB2** | 21q22.3 | 0.634 | 3.54E-47 | **1.64E-45** |
| **ALOX5AP** | 13q12.3 | 0.634 | 3.66E-47 | **1.69E-45** |
| **FPR3** | 19q13.41 | 0.634 | 3.73E-47 | **1.72E-45** |
| **AMPH** | 7p14.1 | 0.634 | 3.78E-47 | **1.73E-45** |
| **CD33** | 19q13.41 | 0.634 | 3.92E-47 | **1.80E-45** |
| **BACH2** | 6q15 | 0.634 | 3.95E-47 | **1.81E-45** |
| **TREM2** | 6p21.1 | 0.634 | 4.30E-47 | **1.96E-45** |
| **ISLR2** | 15q24.1 | 0.634 | 4.64E-47 | **2.11E-45** |
| **AIF1** | 6p21.33 | 0.633 | 4.79E-47 | **2.17E-45** |
| **TSPAN4** | 11p15.5 | 0.633 | 5.29E-47 | **2.40E-45** |
| **PPEF1** | Xp22.13 | 0.633 | 5.41E-47 | **2.44E-45** |
| **PIK3R6** | 17p13.1 | 0.633 | 6.28E-47 | **2.83E-45** |
| **GPR183** | 13q32.3 | 0.633 | 6.61E-47 | **2.97E-45** |
| **MIR99AHG** | 21q21.1 | 0.632 | 7.55E-47 | **3.39E-45** |
| **TPST1** | 7q11.21 | 0.632 | 7.86E-47 | **3.52E-45** |
| **SIGLEC9** | 19q13.41 | 0.632 | 8.32E-47 | **3.72E-45** |
| **ITGAM** | 16p11.2 | 0.632 | 9.28E-47 | **4.13E-45** |
| **ENTPD1** | 10q24.1 | 0.632 | 9.29E-47 | **4.13E-45** |
| **LRRC17** | 7q22.1 | 0.632 | 1.03E-46 | **4.56E-45** |
| **THPO** | 3q27.1 | 0.632 | 1.08E-46 | **4.79E-45** |
| **LAIR1** | 19q13.42 | 0.631 | 1.17E-46 | **5.18E-45** |
| **FLRT2** | 14q31.3 | 0.631 | 1.56E-46 | **6.87E-45** |
| **NCKAP1L** | 12q13.13-q13.2 | 0.631 | 1.61E-46 | **7.08E-45** |
| **PENK** | 8q12.1 | 0.631 | 1.67E-46 | **7.35E-45** |
| **ARHGAP20** | 11q22.3-q23.1 | 0.63 | 1.91E-46 | **8.36E-45** |
| **ADAM33** | 20p13 | 0.63 | 1.97E-46 | **8.61E-45** |
| **TPSB2** | 16p13.3 | 0.63 | 2.01E-46 | **8.75E-45** |
| **MPP1** | Xq28 | 0.63 | 2.07E-46 | **9.01E-45** |
| **CMKLR1** | 12q23.3 | 0.63 | 2.14E-46 | **9.28E-45** |
| **VCAM1** | 1p21.2 | 0.63 | 2.37E-46 | **1.03E-44** |
| **CR1** | 1q32.2 | 0.629 | 2.62E-46 | **1.14E-44** |
| **GATA6** | 18q11.2 | 0.629 | 2.73E-46 | **1.18E-44** |
| **NRXN2** | 11q13.1 | 0.629 | 3.70E-46 | **1.59E-44** |
| **SLC7A7** | 14q11.2 | 0.628 | 4.56E-46 | **1.96E-44** |
| **FAIM2** | 12q13.12 | 0.628 | 5.59E-46 | **2.40E-44** |
| **CDH2** | 18q12.1 | 0.628 | 5.68E-46 | **2.43E-44** |
| **HAS1** | 19q13.41 | 0.627 | 6.41E-46 | **2.74E-44** |
| **TMEM200A** | 6q23.1 | 0.627 | 6.51E-46 | **2.78E-44** |
| **GRIA3** | Xq25 | 0.627 | 6.88E-46 | **2.93E-44** |
| **MKX** | 10p12.1 | 0.627 | 7.02E-46 | **2.98E-44** |
| **GPR34** | Xp11.4 | 0.627 | 7.57E-46 | **3.21E-44** |
| **IGFL2** | 19q13.32 | 0.627 | 8.54E-46 | **3.61E-44** |
| **GDF10** | 10q11.22 | 0.627 | 8.66E-46 | **3.65E-44** |
| **CNTN1** | 12q12 | 0.627 | 8.69E-46 | **3.65E-44** |
| **LILRA2** | 19q13.42 | 0.626 | 9.48E-46 | **3.98E-44** |
| **TSHZ3** | 19q12 | 0.626 | 1.08E-45 | **4.54E-44** |
| **CLMP** | 11q24.1 | 0.626 | 1.22E-45 | **5.10E-44** |
| **SORCS2** | 4p16.1 | 0.626 | 1.32E-45 | **5.51E-44** |
| **FCER1G** | 1q23.3 | 0.625 | 1.60E-45 | **6.68E-44** |
| **ALDH1B1** | 9p13.1 | 0.625 | 1.82E-45 | **7.56E-44** |
| **CYTH4** | 22q13.1 | 0.625 | 1.83E-45 | **7.58E-44** |
| **MCHR1** | 22q13.2 | 0.625 | 1.85E-45 | **7.65E-44** |
| **TLR8** | Xp22.2 | 0.625 | 2.06E-45 | **8.50E-44** |
| **LIX1L** | 1q21.1 | 0.624 | 2.17E-45 | **8.93E-44** |
| **SELPLG** | 12q24.11 | 0.624 | 2.35E-45 | **9.66E-44** |
| **LRIG1** | 3p14.1 | 0.624 | 2.37E-45 | **9.71E-44** |
| **PPFIA2** | 12q21.31 | 0.624 | 2.52E-45 | **1.03E-43** |
| **MT1L** | 16q13 | 0.624 | 2.85E-45 | **1.16E-43** |
| **MGP** | 12p12.3 | 0.623 | 3.43E-45 | **1.40E-43** |
| **TMEM158** | 3p21.31 | 0.623 | 3.59E-45 | **1.46E-43** |
| **MPEG1** | 11q12.1 | 0.623 | 3.61E-45 | **1.47E-43** |
| **RPSAP52** | 12q14.3 | 0.623 | 3.79E-45 | **1.54E-43** |
| **PDCD1LG2** | 9p24.1 | 0.623 | 3.95E-45 | **1.60E-43** |
| **LOXL3** | 2p13.1 | 0.623 | 4.02E-45 | **1.62E-43** |
| **MRC1** | 10p12.33 | 0.623 | 4.15E-45 | **1.67E-43** |
| **CYBB** | Xp21.1-p11.4 | 0.622 | 5.15E-45 | **2.07E-43** |
| **CD37** | 19q13.33 | 0.622 | 5.41E-45 | **2.17E-43** |
| **PTGDS** | 9q34.3 | 0.622 | 7.04E-45 | **2.82E-43** |
| **DRP2** | Xq22.1 | 0.622 | 7.20E-45 | **2.88E-43** |
| **PDLIM7** | 5q35.3 | 0.621 | 7.79E-45 | **3.11E-43** |
| **ATP8B2** | 1q21.3 | 0.621 | 9.49E-45 | **3.78E-43** |
| **PDGFC** | 4q32.1 | 0.621 | 1.06E-44 | **4.21E-43** |
| **NCAM1** | 11q23.2 | 0.62 | 1.22E-44 | **4.84E-43** |
| **CLIC4** | 1p36.11 | 0.62 | 1.47E-44 | **5.83E-43** |
| **SDS** | 12q24.13 | 0.619 | 1.74E-44 | **6.86E-43** |
| **PCBP3** | 21q22.3 | 0.619 | 2.14E-44 | **8.44E-43** |
| **LILRB2** | 19q13.42 | 0.619 | 2.25E-44 | **8.84E-43** |
| **SMIM3** | 5q33.1 | 0.619 | 2.34E-44 | **9.19E-43** |
| **PNMA8A** | 19q13.32 | 0.619 | 2.44E-44 | **9.56E-43** |
| **KLHL38** | 8q24.13 | 0.618 | 2.75E-44 | **1.08E-42** |
| **UNC5A** | 5q35.2 | 0.618 | 2.91E-44 | **1.14E-42** |
| **JAZF1** | 7p15.2-p15.1 | 0.618 | 3.30E-44 | **1.29E-42** |
| **CASQ2** | 1p13.1 | 0.618 | 3.49E-44 | **1.36E-42** |
| **C1QC** | 1p36.12 | 0.618 | 3.65E-44 | **1.42E-42** |
| **ZFHX4-AS1** | 8q21.13 | 0.617 | 3.97E-44 | **1.54E-42** |
| **PCDHGA12** | 5q31.3 | 0.617 | 4.12E-44 | **1.59E-42** |
| **AOC3** | 17q21.31 | 0.617 | 4.23E-44 | **1.63E-42** |
| **LGALS1** | 22q13.1 | 0.617 | 4.42E-44 | **1.70E-42** |
| **GUCY1A1** | 4q32.1 | 0.617 | 4.61E-44 | **1.77E-42** |
| **CD53** | 1p13.3 | 0.617 | 5.27E-44 | **2.02E-42** |
| **C1QA** | 1p36.12 | 0.617 | 5.30E-44 | **2.03E-42** |
| **ANOS1** | Xp22.31 | 0.616 | 5.93E-44 | **2.27E-42** |
| **LTBP2** | 14q24.3 | 0.616 | 6.31E-44 | **2.41E-42** |
| **KIF26B** | 1q44 | 0.616 | 6.46E-44 | **2.46E-42** |
| **NXPH3** | 17q21.33 | 0.616 | 8.05E-44 | **3.06E-42** |
| **BAG2** | 6p12.1 | 0.615 | 8.58E-44 | **3.25E-42** |
| **FGF14** | 13q33.1 | 0.615 | 9.47E-44 | **3.59E-42** |
| **FCGR2C** | 1q23.3 | 0.614 | 1.32E-43 | **5.00E-42** |
| **ARL4C** | 2q37.1 | 0.614 | 1.37E-43 | **5.15E-42** |
| **SCG2** | 2q36.1 | 0.614 | 1.73E-43 | **6.51E-42** |
| **FBXO32** | 8q24.13 | 0.613 | 1.91E-43 | **7.19E-42** |
| **HEG1** | 3q21.2 | 0.613 | 2.33E-43 | **8.76E-42** |
| **ARHGAP31** | 3q13.32-q13.33 | 0.613 | 2.34E-43 | **8.77E-42** |
| **HCK** | 20q11.21 | 0.613 | 2.37E-43 | **8.87E-42** |
| **CD180** | 5q12.3 | 0.613 | 2.78E-43 | **1.04E-41** |
| **LAMA2** | 6q22.33 | 0.612 | 2.96E-43 | **1.10E-41** |
| **SLC2A5** | 1p36.23 | 0.612 | 3.11E-43 | **1.15E-41** |
| **EVI2B** | 17q11.2 | 0.611 | 4.93E-43 | **1.83E-41** |
| **F13A1** | 6p25.1 | 0.611 | 5.22E-43 | **1.93E-41** |
| **CLEC4E** | 12p13.31 | 0.611 | 5.74E-43 | **2.12E-41** |
| **ATP8B4** | 15q21.2 | 0.611 | 5.83E-43 | **2.15E-41** |
| **SIGLEC10** | 19q13.41 | 0.61 | 6.39E-43 | **2.35E-41** |
| **SLC24A3** | 20p11.23 | 0.61 | 6.52E-43 | **2.40E-41** |
| **ANK2** | 4q25-q26 | 0.61 | 8.04E-43 | **2.95E-41** |
| **LILRA6** | 19q13.42 | 0.61 | 8.26E-43 | **3.02E-41** |
| **ANXA13** | 8q24.13 | 0.61 | 8.85E-43 | **3.23E-41** |
| **RASGRF2** | 5q14.1 | 0.61 | 9.07E-43 | **3.31E-41** |
| **DDIT4L** | 4q24 | 0.61 | 9.26E-43 | **3.37E-41** |
| **C1QB** | 1p36.12 | 0.609 | 1.10E-42 | **3.99E-41** |
| **ISM1** | 20p12.1 | 0.609 | 1.21E-42 | **4.40E-41** |
| **SCT** | 11p15.5 | 0.608 | 1.52E-42 | **5.49E-41** |
| **HAVCR2** | 5q33.3 | 0.608 | 1.59E-42 | **5.77E-41** |
| **GGTA1P** | 9q33.2 | 0.608 | 1.70E-42 | **6.14E-41** |
| **DKK2** | 4q25 | 0.608 | 1.78E-42 | **6.40E-41** |
| **SLC16A2** | Xq13.2 | 0.608 | 1.79E-42 | **6.43E-41** |
| **ANKRD29** | 18q11.2 | 0.607 | 2.66E-42 | **9.56E-41** |
| **CREB3L1** | 11p11.2 | 0.607 | 3.00E-42 | **1.08E-40** |
| **AP1S2** | Xp22.2 | 0.606 | 3.03E-42 | **1.08E-40** |
| **KLF9** | 9q21.12 | 0.606 | 3.03E-42 | **1.08E-40** |
| **LAT2** | 7q11.23 | 0.606 | 3.75E-42 | **1.34E-40** |
| **ABCB5** | 7p21.1 | 0.606 | 3.79E-42 | **1.35E-40** |
| **DIO2** | 14q31.1 | 0.606 | 4.33E-42 | **1.54E-40** |
| **LRRK2** | 12q12 | 0.606 | 4.39E-42 | **1.56E-40** |
| **STAB1** | 3p21.1 | 0.605 | 4.84E-42 | **1.71E-40** |
| **JAM2** | 21q21.3 | 0.605 | 4.99E-42 | **1.76E-40** |
| **CD300C** | 17q25.1 | 0.605 | 5.05E-42 | **1.78E-40** |
| **FSTL3** | 19p13.3 | 0.605 | 5.25E-42 | **1.85E-40** |
| **DOCK2** | 5q35.1 | 0.605 | 5.86E-42 | **2.06E-40** |
| **MXRA7** | 17q25.1 | 0.605 | 6.08E-42 | **2.13E-40** |
| **SPEG** | 2q35 | 0.605 | 6.36E-42 | **2.23E-40** |
| **MAOB** | Xp11.3 | 0.604 | 6.86E-42 | **2.40E-40** |
| **CCN5** | 20q13.12 | 0.603 | 9.71E-42 | **3.39E-40** |
| **HHIPL1** | 14q32.2 | 0.603 | 1.02E-41 | **3.55E-40** |
| **SV2B** | 15q26.1 | 0.603 | 1.22E-41 | **4.25E-40** |
| **ABI3BP** | 3q12.2 | 0.603 | 1.23E-41 | **4.26E-40** |
| **ETV1** | 7p21.2 | 0.603 | 1.27E-41 | **4.39E-40** |
| **LRP1** | 12q13.3 | 0.602 | 1.72E-41 | **5.94E-40** |
| **PCSK1** | 5q15 | 0.602 | 1.83E-41 | **6.32E-40** |
| **RGS1** | 1q31.2 | 0.602 | 1.89E-41 | **6.51E-40** |
| **CXORF21** | Xp21.2 | 0.601 | 2.39E-41 | **8.21E-40** |
| **SNED1** | 2q37.3 | 0.601 | 2.59E-41 | **8.88E-40** |
| **HK3** | 5q35.2 | 0.601 | 2.61E-41 | **8.95E-40** |
| **DCLK1** | 13q13.3 | 0.601 | 2.93E-41 | **1.00E-39** |
| **PIP4K2A** | 10p12.2 | 0.6 | 4.36E-41 | **1.49E-39** |

The pink background indicates overlapping genes between UTUC and UBUC.
