# Supplementary figures and images for: High Stromal SFRP2 Expression in Urothelial Carcinoma Confers an Unfavorable Prognosis
Source: Front Oncol. 2022 Mar 16;12:834249. doi: 10.3389/fonc.2022.834249 (PMC8965759; doi:10.3389/fonc.2022.834249)

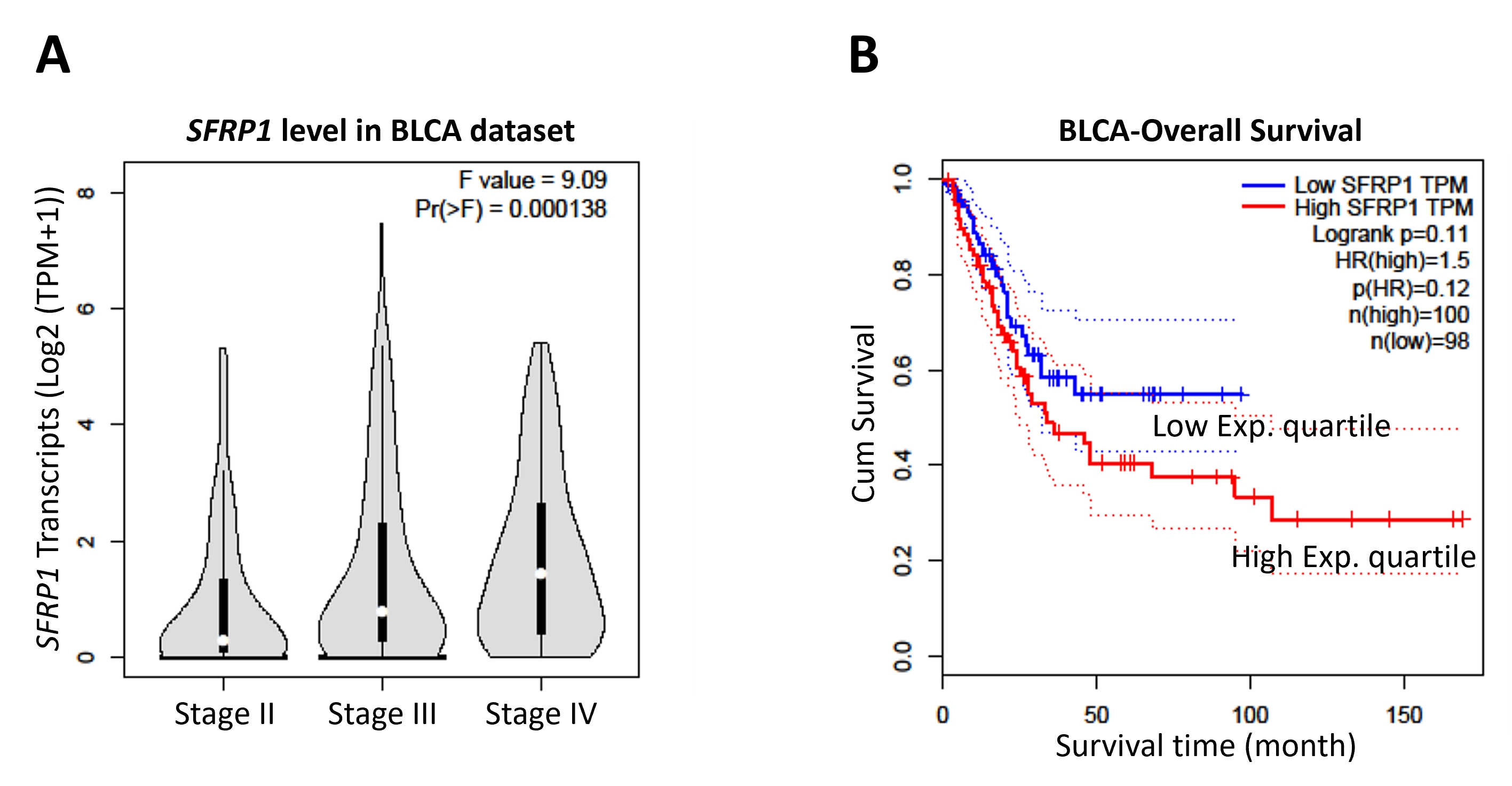

Supplement: Supplementary Figure 1 — High SFRP1 mRNA level is correlated with advanced stage disease but not with inferior overall survival. (A) The correlations between the mRNA levels of SFRP1 and bladder cancer progression. (B) The impact of SFRP1 mRNA levels on overall survival in bladder cancer. These data were acquired from the GEPIA database. BLCA: bladder urothelial carcinoma. [file Image_1.tif]

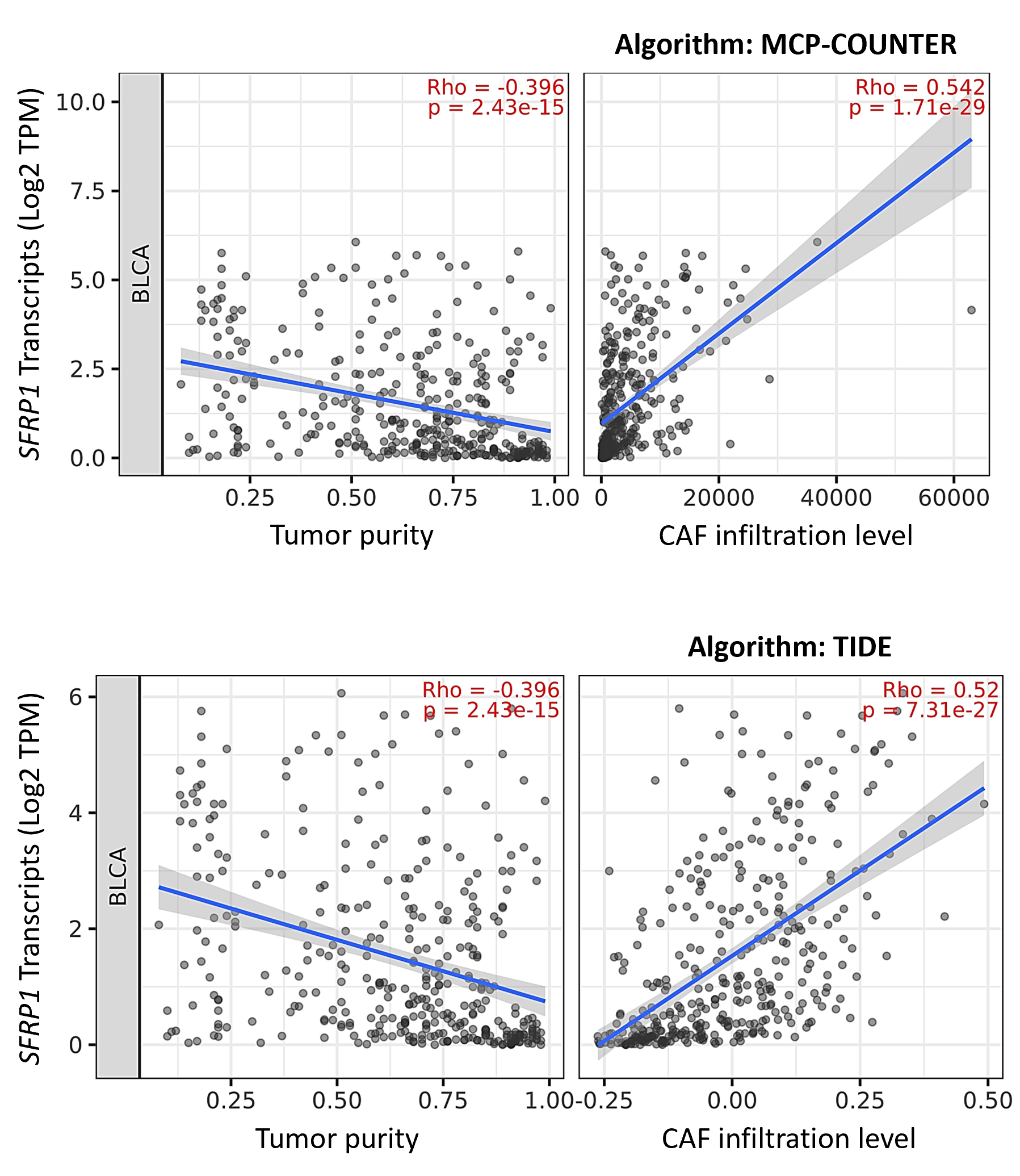

Supplement: Supplementary Figure 2 — SFRP1 transcripts are moderately negatively correlated with tumor purity and positively correlated with CAF infiltration. The correlations among the mRNA levels of SFRP1, tumor purity, and CAF infiltration in bladder cancer. These data were estimated using the MCP-COUNTER and TIDE algorithms from the TIMER2.0 database. BLCA, bladder urothelial carcinoma. [file Image_2.tif]

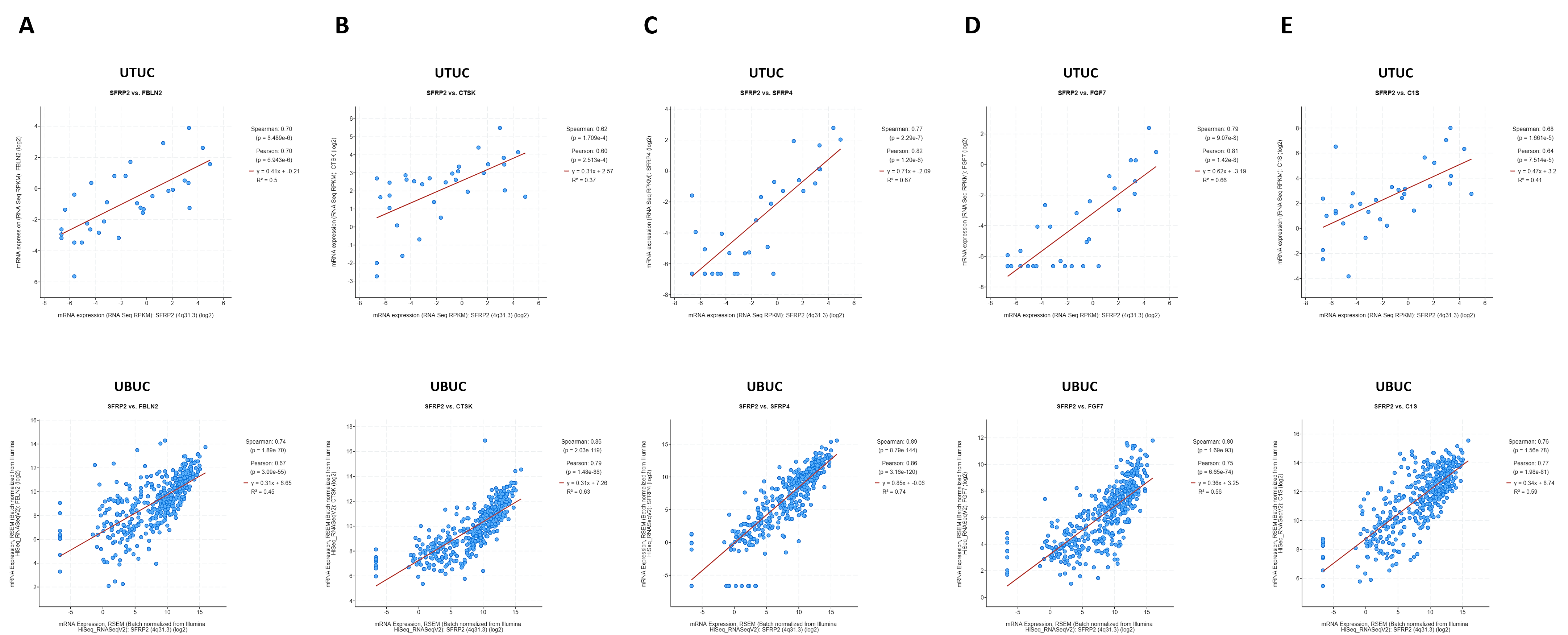

Supplement: Supplementary Figure 3 — Correlations between the expression levels of SFRP2 and its co-upregulated genes. (A–E) Utilizing the cBioPortal web platform, these data were obtained from the TCGA database (n = 411). [file Image_3.tif]

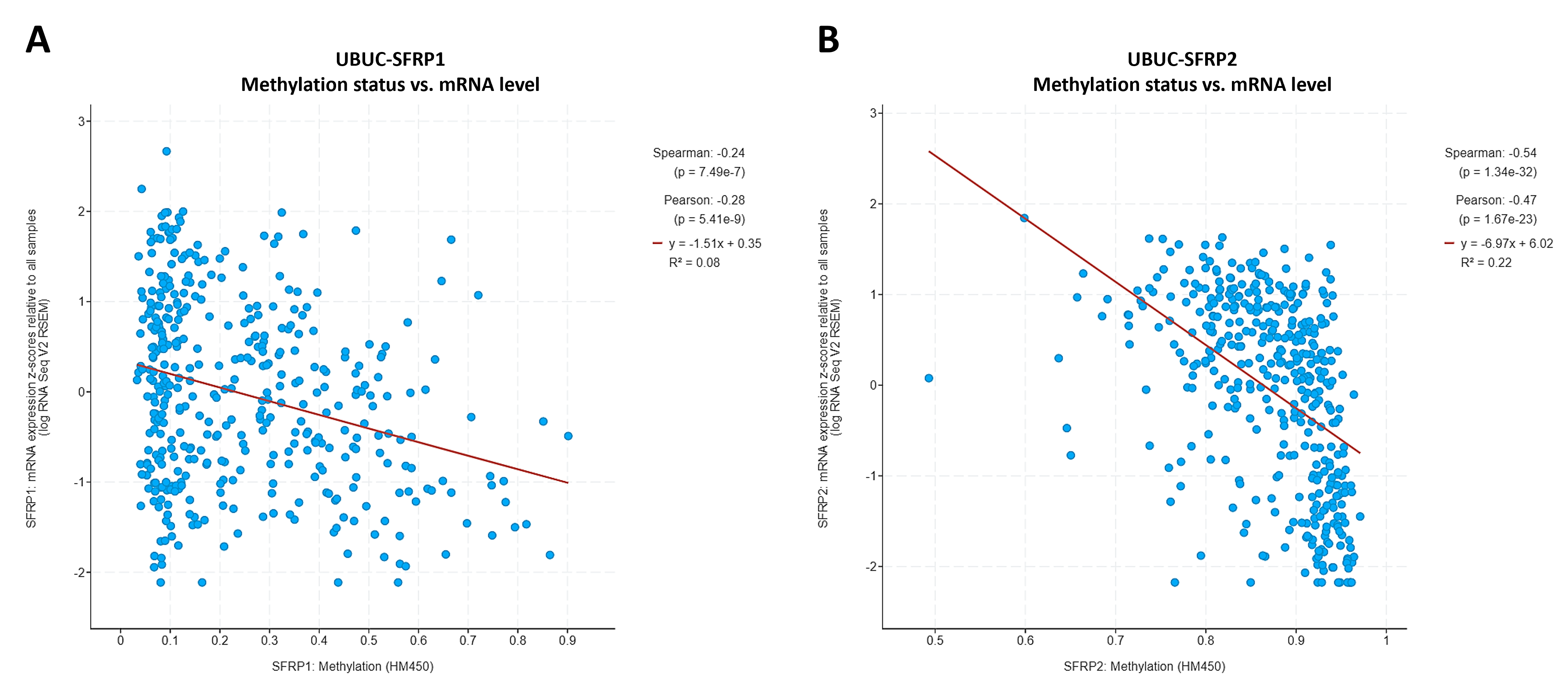

Supplement: Supplementary Figure 4 — Both the mRNA levels of SFRP1 and SFRP2 are not negatively correlated with their methylation status. (A) The correlations between the mRNA levels of SFRP1 and their methylation status. (B) The correlations between the mRNA levels of SFRP2 and their methylation status. Utilizing the cBioPortal web platform, these data were obtained from the TCGA database (n = 413). [file Image_4.tif]

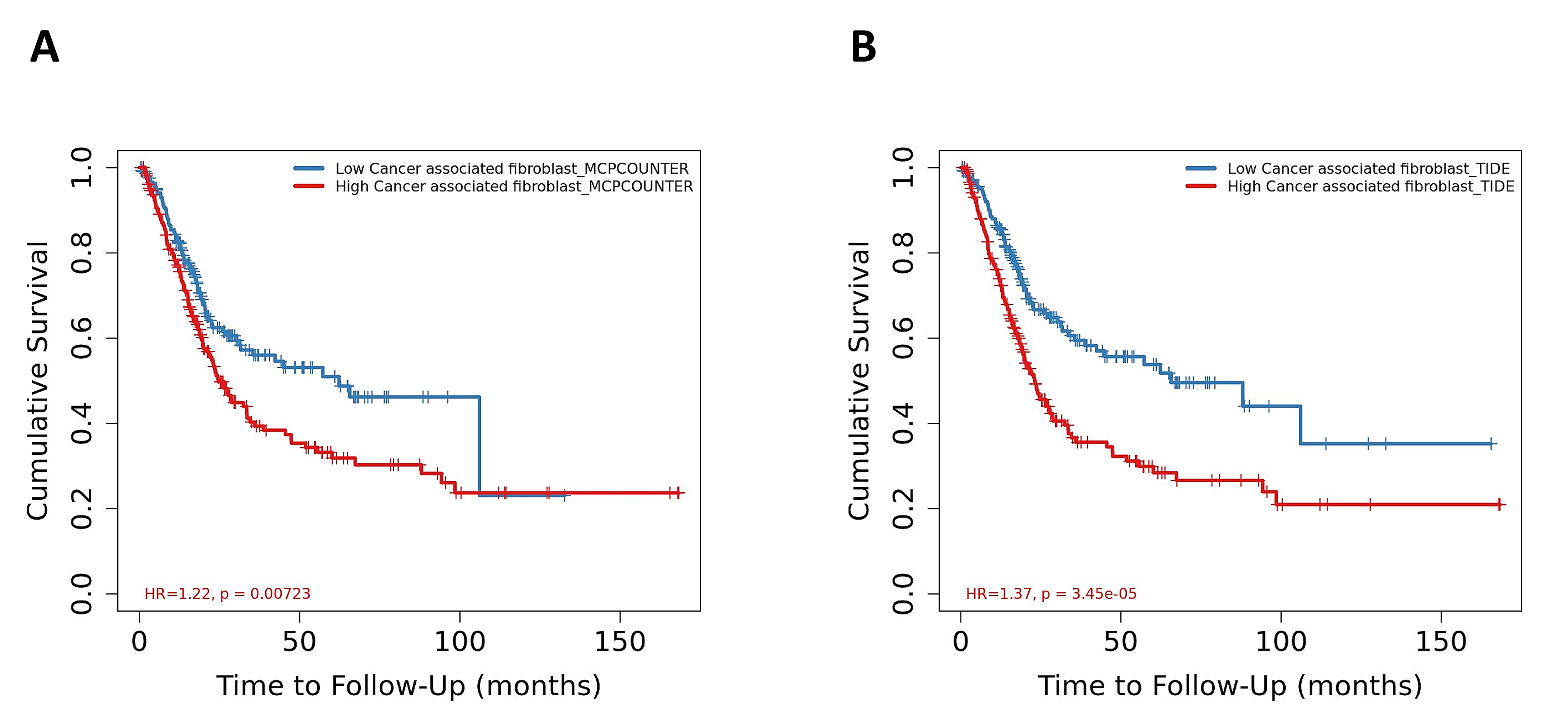

Supplement: Supplementary Figure 5 — High CAF infiltration is significantly correlated with poor cumulative survival. (A, B) The impact of CAF infiltration on cumulative survival in bladder cancer. These data were estimated using the MCP-COUNTER and TIDE algorithms from the TIMER2.0 database. [file Image_5.tif]

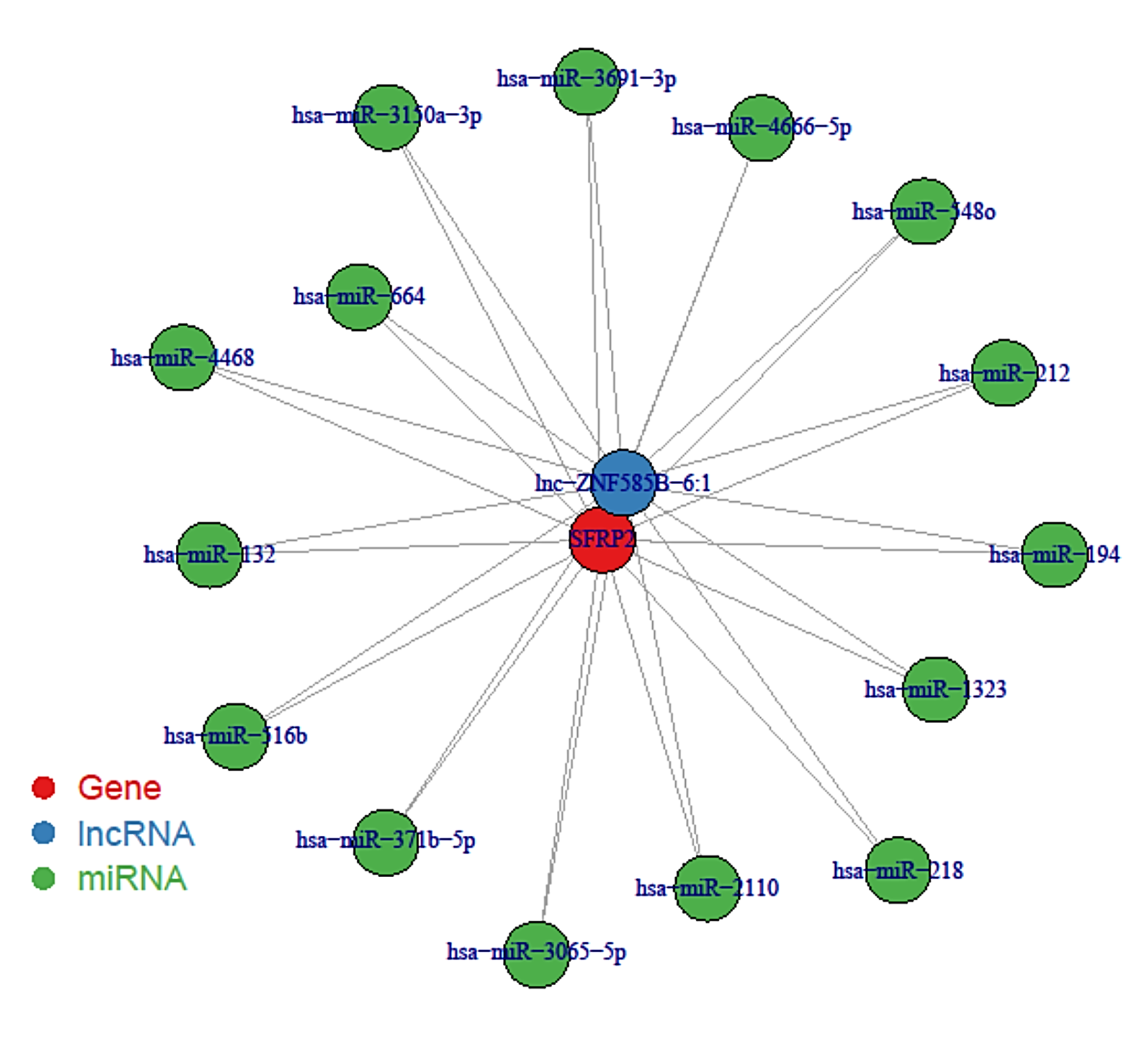

Supplement: Supplementary Figure 6 — Regulatory networks among ZNF585B-6:1, miRNAs, and SFRP2. These data were obtained from the miRTarBase database. [file Image_6.tif]

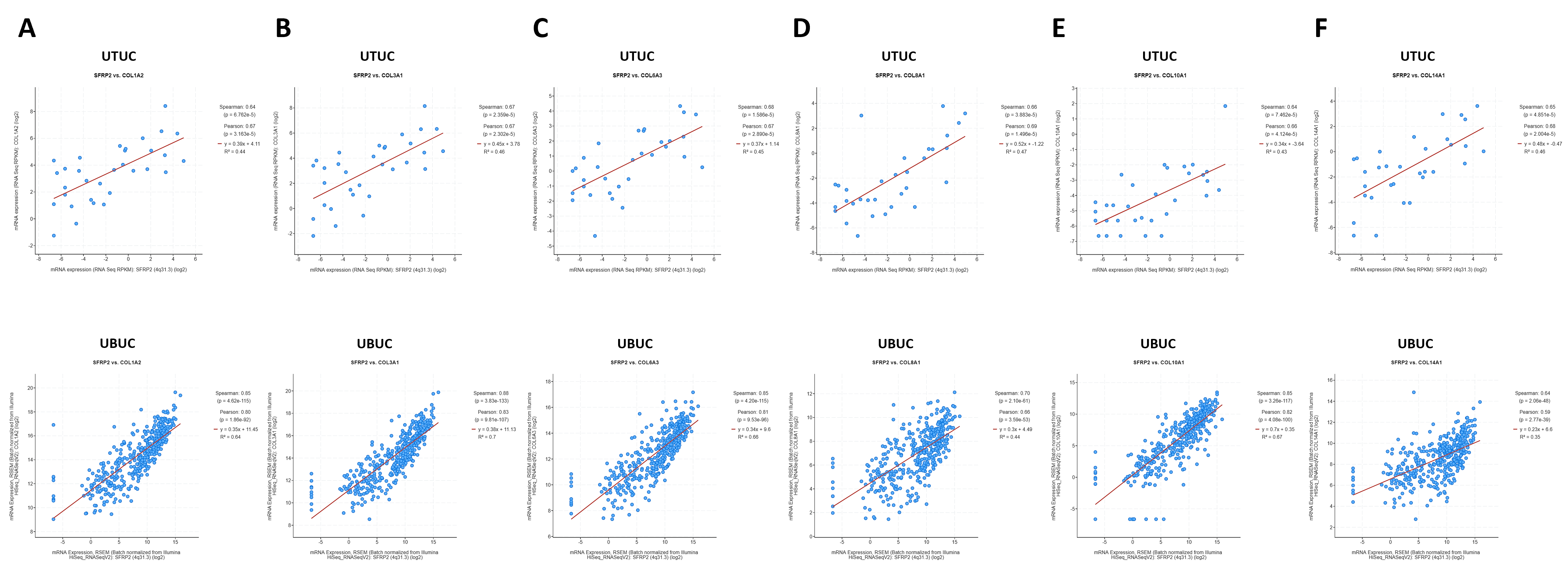

Supplement: Supplementary Figure 7 — Correlations between the expression levels of SFRP2 and collagen family genes. (A–F) Utilizing the cBioPortal web platform, these data were obtained from the TCGA database (n = 411). [file Image_7.tif]

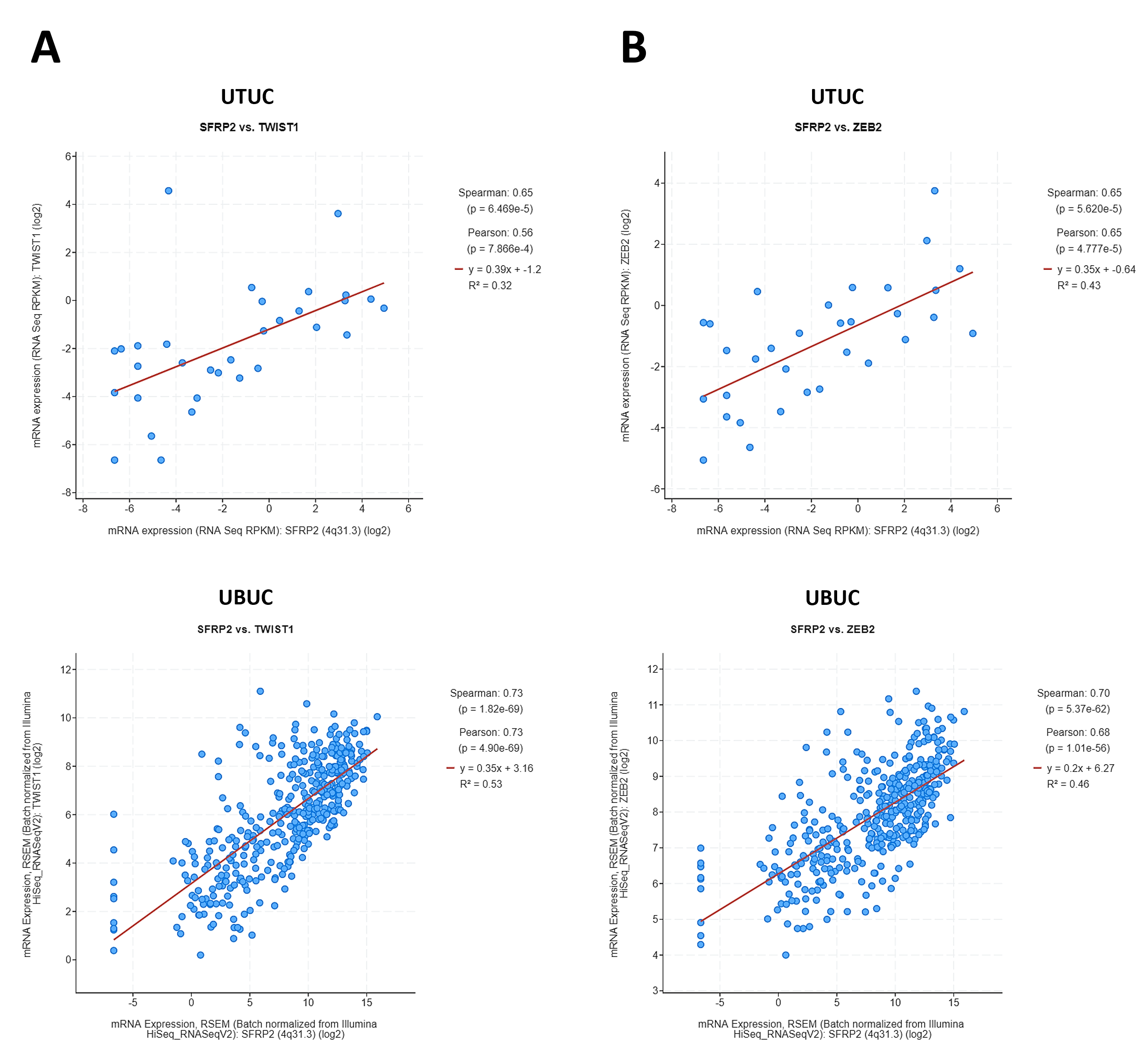

Supplement: Supplementary Figure 8 — Correlations between the expression levels of SFRP2 and EMT markers. (A, B) Utilizing the cBioPortal web platform, these data were obtained from the TCGA database (n = 411). [file Image_8.tif]

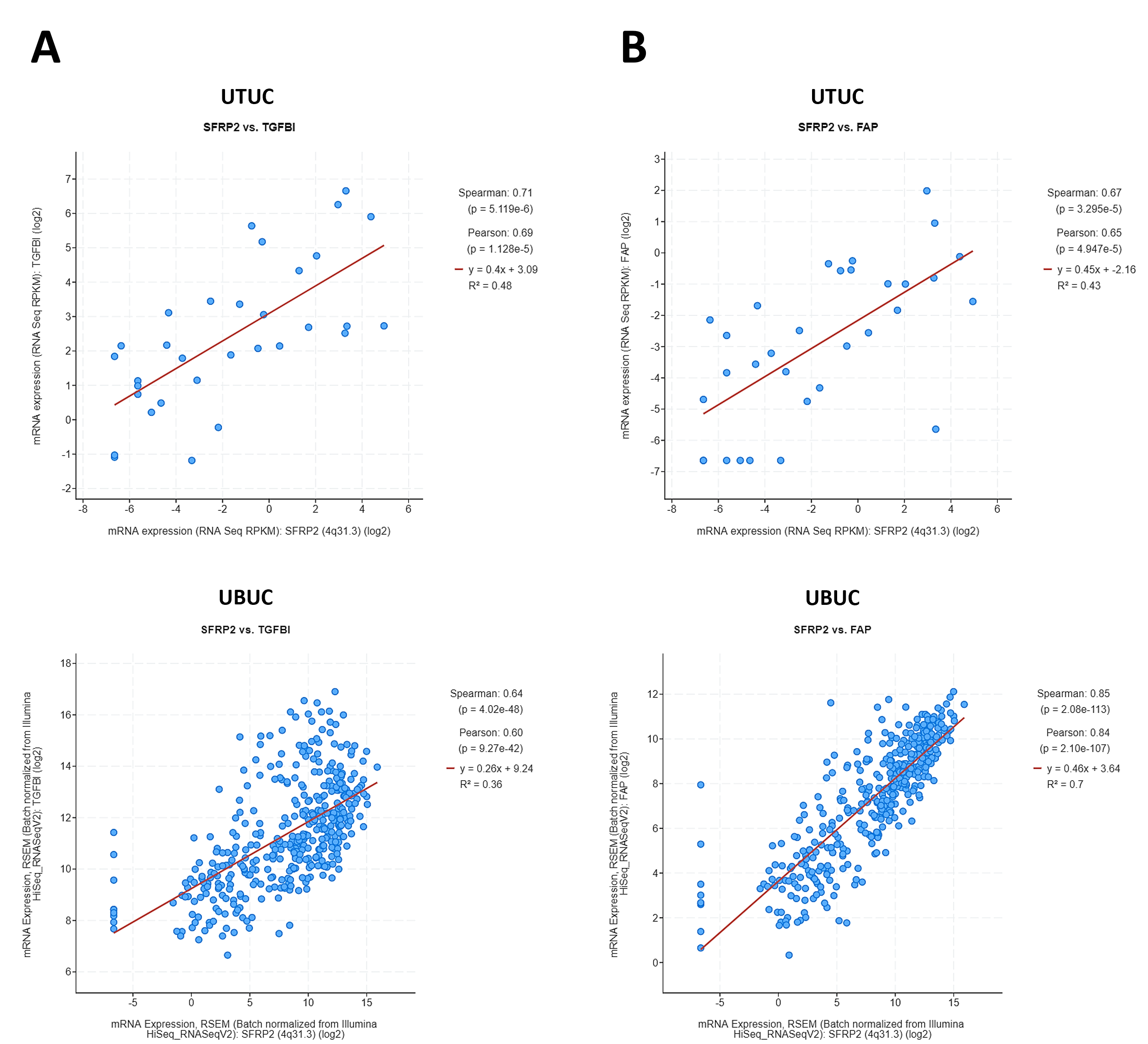

Supplement: Supplementary Figure 9 — Correlations between the expression levels of SFRP2 and TGFB1 and CAF marker. (A, B) Utilizing the cBioPortal web platform, these data were obtained from the TCGA database (n = 411). [file Image_9.tif]
